# Supplementary material for: Conjugates Containing Two and Three Trithiolato-Bridged Dinuclear Ruthenium(II)-Arene Units as In Vitro Antiparasitic and Anticancer Agents
Source: Pharmaceuticals (Basel). 2020 Dec 16;13(12):471. doi: 10.3390/ph13120471 (PMC7767221; doi:10.3390/ph13120471)
Supplement: Supplementary file 1 [file pharmaceuticals-13-00471-s001.pdf]

pharmaceuticals

Supporting information

Conjugates Containing Two and Three Trithiolato-Bridged Dinuclear Ruthenium(II)-Arene Units as *in vitro* Antiparasitic and Anticancer Agents

Valentin Studer[a⊥], Nicoleta Anghel[b⊥], Oksana Desiatkina[a], Timo Felder[a], Ghalia Boubaker[b], Yosra Amdouni[b,c], Jessica Ramseier[b], Martin Hungerbühler[d,e], Christoph Kempf[d,e], Johannes Thomas Heverhagen[d,e], Andrew Hemphill[b,*], Nico Ruprecht[d,e,*], Julien Furrer[a,*] and Emilia Păunescu[a,*]

[a]Department of Chemistry and Biochemistry, University of Bern, Freiestrasse 3, CH-3012 Bern, Switzerland.
[b]Institute of Parasitology, Vetsuisse Faculty, University of Bern, Länggassstrasse 122, CH-3012 Bern, Switzerland.
[c]Laboratoire de Parasitologie, Univ. Manouba, Institution de la Recherche et de l'Enseignement Supérieur Agricoles, École Nationale de Médecine Vétérinaire de Sidi Thabet, 2020 Sidi Thabet, Tunisia.
[d]Department of BioMedical Research, Experimental Radiology, University of Bern, CH-3008 Bern, Switzerland.
[e]Department of Diagnostic, Interventional and Pediatric Radiology, Bern University Hospital, University of Bern, 3010 Bern, Switzerland.

Contents of supporting information

	Page
**Figure S1.** General structure of the dicationic and tricationic conjugates.	S3
**Figure S2.** Structure of acetyl ester **25** and amide **26** derivatives.	S4
**Table S1.** Primary efficacy/cytotoxicity screening of compounds in non-infected HFF cultures and *T. gondii* β-gal tachyzoites cultured in HFF.	S4
**Figure S3.** Dose response curves for compounds **6**, **9**, **10**, **12**, **13**, **15**, **16**, **24** and **25** as inhibitors of *T. gondii* tachyzoites proliferation.	S5
**Table S2.** IC_{50} values (µM) determined after 72 h exposure of HEK293, A2780, A2780cisR, A24 and (D-)A24cisPt8.0 cells to cisplatin (cisPt) and selected trithiolato-bridged dinuclear ruthenium(II)-*p*-cymene compounds.	S6
**Materials and methods - Chemistry**	S7
**1. General**	S7
**2. Abbreviations**	S7
**3. Dithiolato-bridged ruthenium(II)-*p*-cymene intermediates**	S7
**4. Compounds with one trithiolato-bridged ruthenium(II)-*p*-cymene unit**	S9
**5. Tripodal core intermediates**	S13
**6. Compounds with two trithiolato-bridged ruthenium(II)-*p*-cymene units and alkyl diester linkers**	S14
**7. Compounds with two trithiolato-bridged ruthenium(II)-*p*-cymene units and alkyl diamide linkers**	S19
**8. Compounds with two trithiolato-bridged ruthenium(II)-*p*-cymene units and *meta* and *para* substituted diester and diamide linkers**	S24
**9. Compound with three trithiolato-bridged ruthenium(II)-*p*-cymene units with linear distribution**	S28
**10. Compounds with three trithiolato-bridged ruthenium(II)-*p*-cymene units in a star-shape arrangement**	S30
**11. Stability in DMSO-*d*$_6$**	S33
**Figure S4.** ^{1}H-NMR Spectra of **3**-**5**, **25** and **26** recorded in DMSO-d$_6$ at 25°C; (A) recorded 5 min after sample preparation, and (B) sample after >30 days storage at 0-5°C in the dark.	S34
**Figure S5.** ^{1}H-NMR spectra of **6-10** recorded in DMSO-d$_6$ at 25°C; (A) recorded 5 min after sample preparation, and (B) sample after >30 days storage at 0-5°C in the dark.	S35
**Figure S6.** ^{1}H-NMR spectra of **11-14** recorded in DMSO-d$_6$ at 25°C; (A) recorded 5 min after sample preparation, and (B) sample after >30 days storage at 0-5°C in the dark.	S36
**Figure S7.** ^{1}H-NMR spectra of **15-18** recorded in DMSO-d$_6$ at 25°C; (A) recorded 5 min after sample preparation, and (B) sample after >30 days storage at 0-5°C in the dark.	S37
**Figure S8.** ^{1}H-NMR spectra of **23** and **24** recorded in DMSO-d$_6$ at 25°C; (A) recorded 5 min after sample preparation, and (B) sample after > 30 days storage at 0-5°C in the dark.	S38
**References**	S38

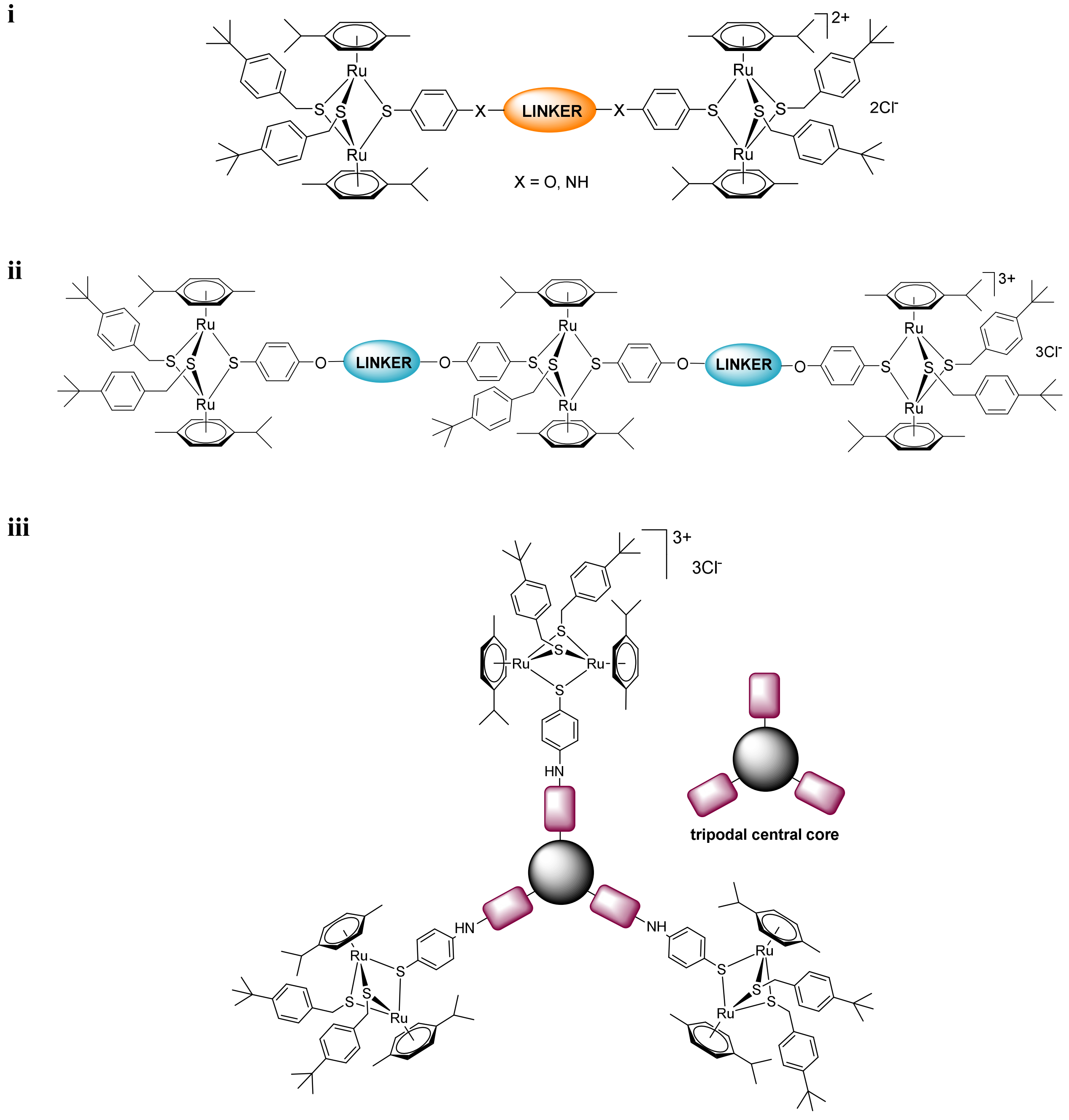

Figure S1. General structure of the dicationic conjugates containing two thiolato-bridged di-ruthenium units (i), and of the tricationic conjugates with three thiolato binuclear ruthenium units disposed in a linear 'beads-on-a-string' (ii) or in a tri-geminal 'star-shape' arrangement (iii).

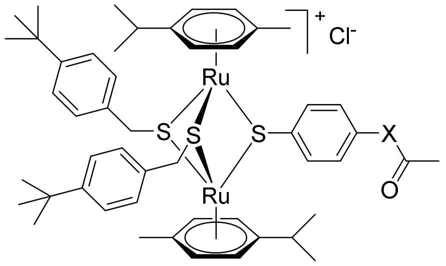

25, X = O
26, X = NH

Figure S2. Structure of acetyl ester **25** and amide **26** derivatives.[1].

Table S1. Primary efficacy/cytotoxicity screening of compounds in non-infected HFF cultures and *T. gondii* β-gal tachyzoites cultured in HFF. The compounds selected for determination of IC_{50} values against *T. gondii* β-gal are tagged with *. [a]Data for compounds **3**, **4** and **5** were previously reported[2]. [b]Data for **25** and **26** are part of another study[1].

Compound	HFF viability (%)		*T. gondii* β-gal growth (%)	
	0.1 µM	1 µM	0.1 µM	1 µM
Compounds with one trithiolato-bridged ruthenium(II)-p-cymene unit				
**3***,[a]	76 ± 6	46 ± 6	66 ± 14	2 ± 0
**4***,[a]	74 ± 2	48 ± 1	57 ± 1	2 ± 0
**5***,[a]	62 ± 8	56 ± 7	3 ± 1	2 ± 0
**25***,[b]	74 ± 2	52 ± 3	5 ± 0	2 ± 1
**26**[b]	62 ± 7	27 ± 1	4 ± 0	3 ± 1
Compounds with two trithiolato-bridged ruthenium(II)-p-cymene units and alkyl diester linkers				
**6***	82 ± 4	54 ± 3	2 ± 0	1 ± 0
**7**	76 ± 2	25 ± 4	68 ± 2	0 ± 6
**8**	79 ± 4	24 ± 9	2 ± 0	1 ± 0
**9***	90 ± 14	73 ± 1	4 ± 2	1 ± 0
**10***	77 ± 10	54 ± 3	42 ± 16	1 ± 0
Compounds with two trithiolato-bridged ruthenium(II)-p-cymene units and alkyl diamide linkers				
**11**	99 ± 7	98 ± 5	132 ± 12	128 ± 17
**12***	93 ± 8	78 ± 0	130 ± 15	2 ± 0
**13***	75 ± 2	97 ± 10	112 ± 8	2 ± 0
**14**	85 ± 7	87 ± 5	76 ± 13	18 ± 5
Compounds with two trithiolato-bridged ruthenium(II)-p-cymene units and meta and para substituted diester and diamide linkers				
**15***	103 ± 5	83 ± 3	5 ± 2	2 ± 0
**16***	90 ± 2	74 ± 1	4 ± 11	1 ± 0
**17**	102 ± 2	106 ± 7	117 ± 7	109 ± 6
**18**	94 ± 8	96 ± 5	97 ± 12	111 ± 3
Compound with three trithiolato-bridged ruthenium(II)-p-cymene units with linear distribution				
**20**	79 ± 6	25 ± 2	2 ± 0	0 ± 1
Compounds with three trithiolato-bridged ruthenium(II)-p-cymene units in a star-shape arrangement				
**23**	87 ± 6	90 ± 3	106 ± 7	92 ± 0
**24***	97 ± 17	70 ± 1	88 ± 7	2 ± 0

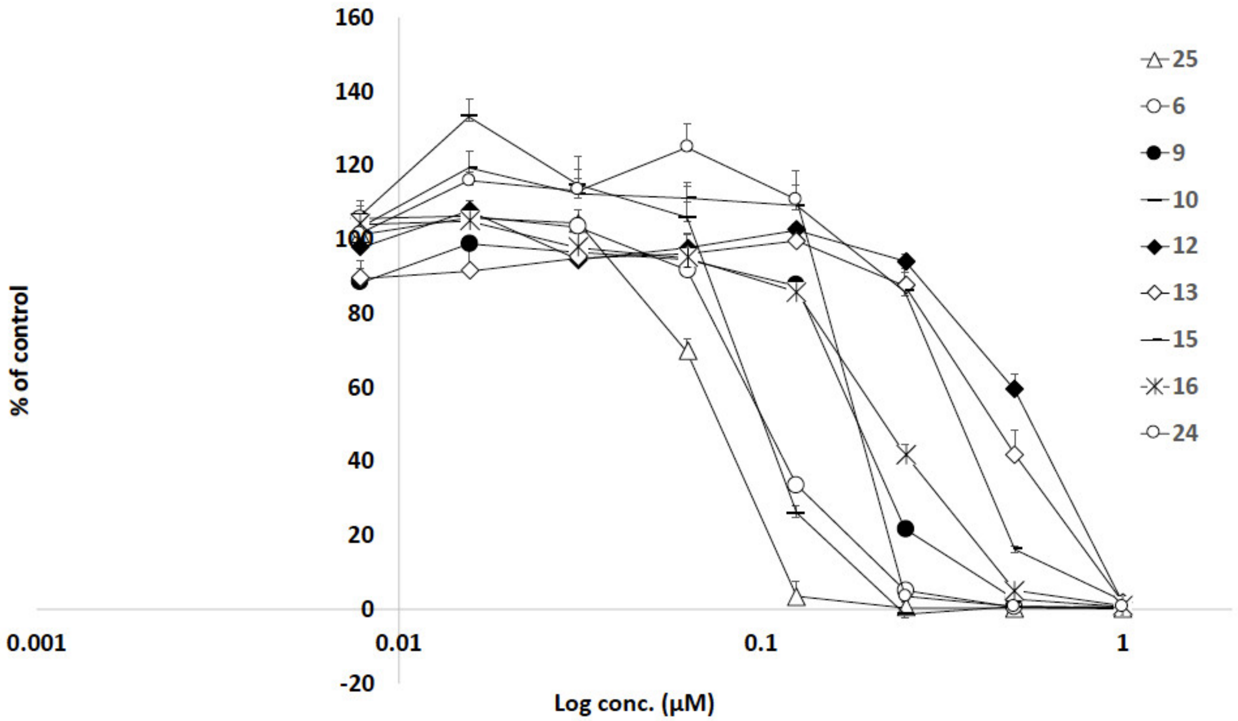

Figure S3. Dose response curves for compounds **6**, **9**, **10**, **12**, **13**, **15**, **16**, **24** and **25** as inhibitors of *T. gondii* tachyzoites proliferation. Bars represent standard deviation for each tested concentration. Eight concentrations ranged between 1 and 0.007 μM were tested, each in six replicates/experiment. Data for pyrimethamine and compounds **3**, **4** and **5** were previously reported[2].

Table S2. IC_{50} values (μM) determined after 72 h exposure of human embryonic kidney HEK293 cells, human ovarian A2780 and A2780cisR cisplatin sensitive and resistant cancer cells, and human lung adenocarcinoma cells wild type A24 and de-induced (D-)A24cisPt8.0 subline, to cisplatin (cisPt) and the trithiolato-bridged dinuclear ruthenium(II)-*p*-cymene compounds considered for this study.*.

**Compound**	**IC_{50} (μM)**				
	**HEK293**	**A2780**	**A2780cisR**	**A24**	**(D-)A24cisPt8.0**
**cisPt**	0.515 ± 0.064	0.210 ± 0.119	4.085 ± 0.210	0.333 ± 0.075	8.889 ± 0.446
Compounds with one trithiolato-bridged ruthenium(II)-p-cymene unit					
**3**	0.231 ± 0.036	0.101 ± 0.009	0.084 ± 0.012	0.106 ± 0.020	0.090 ± 0.005
**4**	0.185 ± 0.034	0.098 ± 0.012	0.086 ± 0.007	0.107 ± 0.014	0.097 ± 0.006
**5**	0.144 ± 0.035	0.046 ± 0.008	0.036 ± 0.003	0.041 ± 0.024	0.023 ± 0.002
Compounds with two trithiolato-bridged ruthenium(II)-p-cymene units and alkyl diester linkers					
**6**	0.092 ± 0.024	0.062 ± 0.012	0.046 ± 0.007	0.059 ± 0.023	0.049 ± 0.004
**7**	0.085 ± 0.026	0.097 ± 0.041	0.072 ± 0.008	0.033 ± 0.002	0.053 ± 0.007
**9**	0.096 ± 0.044	0.089 ± 0.018	0.131 ± 0.009	0.041 ± 0.007	0.058 ± 0.009
Compounds with two trithiolato-bridged ruthenium(II)-p-cymene units and alkyl diamide linkers					
**11**	0.192 ± 0.027	0.085 ± 0.014	0.109 ± 0.019	0.056 ± 0.007	0.099 ± 0.009
**12**	0.174 ± 0.029	0.128 ± 0.043	0.300 ± 0.031	0.121 ± 0.009	0.214 ± 0.013
**13**	0.148 ± 0.024	0.124 ± 0.040	0.329 ± 0.017	0.142 ± 0.043	0.271 ± 0.052
Compounds with two trithiolato-bridged ruthenium(II)-p-cymene units and meta and para substituted diester and diamide linkers					
**15**	0.043 ± 0.019	0.067 ± 0.001	0.094 ± 0.023	0.058 ± 0.011	0.036 ± 0.003
**17**	0.447 ± 0.043	0.416 ± 0.106	0.644 ± 0.080	0.240 ± 0.016	0.090 ± 0.003
**18**	0.274 ± 0.103	0.316 ± 0.012	0.650 ± 0.089	0.275 ± 0.054	0.228 ± 0.048
Compounds with three trithiolato-bridged ruthenium(II)-p-cymene units with linear distribution					
**20**	0.074 ± 0.019	0.065 ± 0.017	0.033 ± 0.003	0.044 ± 0.012	0.039 ± 0.008
Compounds with three trithiolato-bridged ruthenium(II)-p-cymene units in a star-shape arrangement					
**24**	0.193 ± 0.005	0.188 ± 0.030	0.277 ± 0.029	0.232 ± 0.049	0.167 ± 0.018

*Values are given as the mean ± SD.

Materials and methods – Chemistry

1. General

$RuCl_3 \cdot 3H_2O$ was obtained from Fluorochem, and all other chemicals were purchased from Aldrich, AlfaAesar, Acros, ABCR, or TCI Chemicals and used without further purification. Reactions were performed under an inert atmosphere of N_2 using Schlenk techniques with dry solvents preserved on molecular sieves dried (Across Organics). The dimer [Ru(η^6-*p*-cymene)Cl]$_2$Cl$_2$ was prepared and purified according to literature procedures[3]. ^{1}H (400.13 MHz) and ^{13}C (100.62 MHz) NMR spectra were recorded on a Bruker Avance II 400 spectrometer at 298 K. ^{19}F (282.40 MHz) spectra were recorded on a Bruker Avance III 300 spectrometer at 298 K. The chemical shifts are reported in parts per million (ppm) and referenced to residual solvent peaks[4] ($CDCl_3$, ^{1}H δ 7.26, ^{13}C{^{1}H} δ 77.16 ppm; MeOD-d$_4$, ^{1}H δ 3.31, ^{13}C{^{1}H} δ 49.00 ppm, DMSO-d$_6$, ^{1}H δ 2.50, ^{13}C{^{1}H} δ 39.52 ppm) and coupling constants (*J*) are reported in hertz (Hz). High resolution electrospray ionization mass spectra (HRESI-MS) were carried out by the Mass Spectrometry and Protein Analyses Services at DCB and were obtained on a LTQ Orbitrap XL ESI (Thermo) operated in positive ion mode. Thermal elemental analyses were carried out by the Mass Spectrometry and Protein Analyses Services at DCB and were obtained on a Flash 2000 Organic Elemental Analyzer (Thermo Scientific). Reactions were monitored by TLC using Macherey-Nagel TLC silica gel coated aluminium sheets Alugram® Xtra SIL G/UV$_{254}$ and visualized with UV at 254 nm. Preparative TLC purifications were performed using Macherey-Nagel TLC silica gel glass pre-coated TCL plates SIL G-25 UV$_{254}$, and silica extracts were filtered on Macherey-Nagel disposable syringe filters Chromafil® Xtra PTFE-20-25 (pore size 0.20 µm). Compounds were purified by column flash chromatography on silica gel Sigma-Aldrich (60 Å, 230-400 mesh) using the elution systems indicated.

2. Abbreviations

DIPEA - *N,N*-Diisopropylethylamine
DMAP - 4-(Dimethylamino)-pyridine
EDCI - *N*-(3-Dimethylaminopropyl)-*N'*-ethylcarbodiimide hydrochloride
HOBt·H$_2$O - 1-Hydroxybenzotriazole hydrate
TEA - Triethylamine

3. Dithiolato-bridged ruthenium(II)-*p*-cymene intermediates

Synthesis of ([(η^6-p-MeC$_6$H$_4$Pr^i)$_2$Ru$_2$(μ_2-SCH$_2$C$_6$H$_4$-p-Bu^t)Cl$_2$]) (1)

The dithiolato precursor **1** was prepared and purified according to literature procedure[5]. To a solution of the dimer [Ru(η^6-*p*-MeC$_6$H$_4$Pr^i)Cl]$_2$Cl$_2$ (3.00 g, 4.899 mmol, 1 equiv.) in EtOH (300 mL) at 0°C under inert atmosphere (N$_2$) was added dropwise a solution of 4-*tert*-butylbenzenemethanethiol (1.767 g, 9.797 mmol, 2 equiv.) in EtOH (10 mL). The reaction mixture was stirred at 0°C for further 4 h and then concentrated at 40°C under reduced pressure to 3 mL. The product was precipitated with Et$_2$O (100 mL) and the obtained suspension was stored at 0°C overnight. The precipitate was isolated by filtration, washed with Et$_2$O (3 x 50 mL) and dried to afford **1** as an orange solid (4.03 g, 4.475 mmol, yield 91%).

^{1}H-NMR (CDCl$_3$) δ_H, ppm: 7.49 (4H, d, 4xS-CH$_2$-(*Ar*)C-CH-C$\underline{H}$-C-C(CH$_3$)$_3$, $^3J_{H,H}$ = 8.1 Hz), 7.32 (4H, d, 4xS-CH$_2$-(*Ar*)C-C$\underline{H}$-CH-C-C(CH$_3$)$_3$, $^3J_{H,H}$ = 8.1 Hz), 4.93 (2H, m, 2xCH$_3$-(*Ar*)C-CH-C$\underline{H}$-C), 4.80 (4H, m, 2xCH$_3$-(*Ar*)C-CH-C$\underline{H}$-C, 2xCH$_3$-(*Ar*)C-C$\underline{H}$-CH-C), 4.11 (2H, d, 2xS-C$\underline{H}_2$-(*Ar*)C-CH-CH-C-C(CH$_3$)$_3$, $^2J_{H,H}$ = 11.3 Hz), 3.88 (2H, m, 2xCH$_3$-(*Ar*)C-C$\underline{H}$-CH-C), 3.32 (2H, d, 2xS-C$\underline{H}_2$-(*Ar*)C-CH-CH-C-C(CH$_3$)$_3$, $^2J_{H,H}$ = 11.3 Hz), 2.82 (2H, sept, 2x(*Ar*)C-CH-CH-C-C$\underline{H}$(CH$_3$)$_2$, $^3J_{H,H}$ = 6.9 Hz), 1.90 (6H, s, 2xC$\underline{H}_3$-(*Ar*)C-CH-CH-C), 1.33 (18H, s, 2xS-CH$_2$-(*Ar*)C-CH-CH-C-C(C$\underline{H}_3$)$_3$), 1.11-1.25 (12H, m, 2x(*Ar*)C-CH-CH-C-CH(C$\underline{H}_3$)$_2$).

^{13}C-NMR (CDCl$_3$) δ_C, ppm: 150.1 (2C, 2xS-CH$_2$-(*Ar*)C-CH-CH-$\underline{C}$-C(CH$_3$)$_3$), 137.9 (2C, 2xS-CH$_2$-(*Ar*)$\underline{C}$-CH-CH-C-C(CH$_3$)$_3$), 130.3 (4C, 4xS-CH$_2$-(*Ar*)C-$\underline{C}$H-CH-C-C(CH$_3$)$_3$), 124.6 (4C, 4xS-CH$_2$-(*Ar*)C-CH-$\underline{C}$H-C-C(CH$_3$)$_3$), 105.8 (2C, 2xCH$_3$-(*Ar*)C-CH-CH-$\underline{C}$), 97.3 (2C, 2xCH$_3$-(*Ar*)$\underline{C}$-CH-CH-C), 85.4 (2C, 2xCH$_3$-(*Ar*)C-CH-$\underline{C}$H-C), 84.1 (2C, 2xCH$_3$-(*Ar*)C-CH-$\underline{C}$H-C), 83.0 (2C, 2xCH$_3$-(*Ar*)C-$\underline{C}$H-CH-C), 79.5 (2C, 2xCH$_3$-(*Ar*)C-$\underline{C}$H-CH-C), 36.6 (2C, 2xS-$\underline{C}$H$_2$-(*Ar*)C-CH-CH-C-C(CH$_3$)$_3$), 34.7 (2C, 2xS-CH$_2$-(*Ar*)C-CH-CH-C-$\underline{C}$(CH$_3$)$_3$), 31.6 (6C, 2xS-CH$_2$-(*Ar*)C-CH-CH-C-C($\underline{C}$H$_3$)$_3$), 30.0 (2C, 2x(*Ar*)CH-CH-C-$\underline{C}$H(CH$_3$)$_2$), 23.4 (2C, (*Ar*)CH-CH-C-CH($\underline{C}$H$_3$)$_2$), 21.6 (2C, (*Ar*)CH-CH-C-CH($\underline{C}$H$_3$)$_2$), 18.8 (2C, 2x$\underline{C}$H$_3$-(*Ar*)C-CH-CH).

ESI-MS(+): *m/z* found 865.1779 [M-Cl]$^+$, calcd. for C$_{42}$H$_{58}$ClRu$_2$S$_2^+$ 865.1750, the isotopic pattern corresponds well to the calculated one.

Synthesis of ([(η^6-p-MeC$_6$H$_4$Pr^i)$_2$Ru$_2$(μ_2-SC$_6$H$_4$-p-OH)Cl$_2$]) (2)

The dithiolato precursor **2** was prepared and purified according to literature procedure[2]. To a solution of the dimer [Ru(η^6-*p*-MeC$_6$H$_4$Pr^i)Cl]$_2$Cl$_2$ (0.816 g, 1.332 mmol, 1 equiv.) in EtOH (130 mL) at 0°C under inert atmosphere (N$_2$) was added dropwise a solution of 4-hydroxy-benzenethiol (0.340 g, 2.664 mmol, 2 equiv.) in EtOH (15 mL). The reaction mixture was stirred at 0°C for further 4 h and then concentrated at 40°C under reduced pressure. Purification by column chromatography using CH$_2$Cl$_2$/CH$_3$OH (9:1, v/v) mixture afforded **2** as an orange solid (0.960 g, 1.212 mmol, yield 91%).

^{1}H-NMR ($CDCl_3$) δ_H, ppm: 7.74-7.91 (4H, m, 4xS-(*Ar*)C-C*H*-CH-C-OH), 6.91-7.03 (4H, m, 4xS-(*Ar*)C-CH-C*H*-C-OH), 5.11-5.24 (4H, m, 2xCH_3-(*Ar*)C-CH-C*H*-C, 2xCH_3-(*Ar*)C-C*H*-CH-C), 4.00-5.11 (2H, m, 2xCH_3-(*Ar*)C-C*H*-CH-C), 4.86-5.00 (2H, m, 2xCH_3-(*Ar*)C-C*H*-CH-C), 2.37 (2H, sept, 2x(*Ar*)C-CH-CH-C-C*H*(CH_3)$_2$, $^3J_{H,H}$ = 6.7 Hz), 1.15 (6H, s, 2xC*H$_3$*-(*Ar*)C-CH-CH-C), 1.00-1.15 (12H, m, 2x(*Ar*)C-CH-CH-C-CH(C*H$_3$*)$_2$).

^{13}C-NMR ($CDCl_3$) δ_C, ppm: 158.9 (2C, 2xS-(*Ar*)C-CH-CH-*C*-OH), 133.7 (2C, 2xS-(*Ar*)*C*-CH-CH-C-OH), 133.1 (4C, 4xS-(*Ar*)C-*C*H-CH-C-OH), 116.8 (4C, 4xS-(*Ar*)C-CH-*C*H-C-OH), 106.1 (2C, 2xCH_3-(*Ar*)C-CH-CH-*C*), 97.1 (2C, 2xCH_3-(*Ar*)*C*-CH-CH-C), 84.2 (4C, 4CH_3-(*Ar*)C-CH-*C*H-C), 80.6 (4C, 4CH_3-(*Ar*)C-*C*H-CH-C), 29.8 (2C, 2x(*Ar*)CH-CH-C-*C*H(CH_3)$_2$), 22.3 (4C, 2x(*Ar*)CH-CH-C-CH(*C*H$_3$)$_2$), 7.82 (2C, 2x*C*H$_3$-(*Ar*)C-CH-CH).

ESI-MS(+): *m/z* found 757.0077 $[M-Cl]^+$, calcd. for $C_{32}H_{38}ClO_2Ru2S_2^+$ 757.0083, the isotopic pattern corresponds well to the calculated one.

4. Compounds with one trithiolato-bridged ruthenium(II)-*p*-cymene unit

Synthesis of [(η^6-p-MeC$_6$H$_4$Pr^i)$_2$Ru$_2$(μ_2-SCH$_2$C$_6$H$_4$-p-Bu^t)$_2$(μ_2-SC$_6$H$_4$-p-OH)]Cl (3)

The mixed hydroxy compound **3** was prepared and purified by adapting literature procedures[6]. To a solution of dithiolato precursor **1** (1.498g, 1.666 mmol, 1 equiv.) in EtOH (150 mL) was added dropwise at reflux under inert atmosphere (N_2) a solution of 4-hydroxy-benzenethiol (0.618 g, 4.904 mmol, 3 equiv.) in EtOH (15 mL). The reaction mixture was further refluxed overnight and the reaction evolution was verified by TLC and ^{1}H-NMR. The reaction mixture was cooled to r.t. and concentrated. Purification by column chromatography using CH_2Cl_2/CH_3OH (10:1, v/v) afforded **3** as an orange solid (1.35 g, 1.364 mmol, yield 82%).

^{1}H-NMR ($CDCl_3$) δ_H, ppm: 10.36 (1H, s, OH), 7.38-7.48 (10H, m, 2xS-(*Ar*)C-C*H*-CH-C-OH, 4xS-CH_2-(*Ar*)C-C*H*-CH-C-C(CH_3)$_3$, 4xS-CH_2-(*Ar*)C-CH-C*H*-C-C(CH_3)$_3$), 7.24 (2H, d, 2xS-(*Ar*)C-CH-C*H*-C-OH, $^3J_{H,H}$ = 8.7 Hz), 4.98 (2H, d, 2xCH_3-(*Ar*)C-CH-C*H*-C, $^3J_{H,H}$ = 5.8 Hz), 4.88 (2H, d, 2xCH_3-(*Ar*)C-C*H*-CH-C, $^3J_{H,H}$ = 5.8 Hz), 4.70 (2H, d, 2xCH_3-(*Ar*)C-CH-C*H*-C, $^3J_{H,H}$ = 5.8 Hz), 4.58 (2H, d, 2xCH_3-(*Ar*)C-C*H*-CH-C, $^3J_{H,H}$ = 5.8 Hz), 3.54 (2H, s, S-C*H$_2$*-(*Ar*)C-CH-CH-C-C(CH_3)$_3$), 3.36 (2H, s, S-C*H$_2$*-(*Ar*)C-CH-CH-C-C(CH_3)$_3$), 1.97 (2H, sept, 2x(*Ar*)C-CH-CH-C-C*H*(CH_3)$_2$, $^3J_{H,H}$ = 6.9 Hz), 1.69 (6H, s, 2xC*H$_3$*-(*Ar*)C-CH-CH-C), 1.36 (9H, s, S-CH_2-(*Ar*)C-CH-CH-C-C(C*H$_3$*)$_3$), 1.34 (9H, s, S-CH_2-(*Ar*)C-CH-CH-C-C(C*H$_3$*)$_3$), 0.97 (6H, d, 2x(*Ar*)C-CH-CH-C-CH(C*H$_3$*)$_2$, $^3J_{H,H}$ = 6.9 Hz), 0.92 (6H, d, 2x(*Ar*)C-CH-CH-C-CH(C*H$_3$*)$_2$, $^3J_{H,H}$ = 6.9 Hz).

^{13}C-NMR ($CDCl_3$) δ_C, ppm: 160.1 (1C, S-(*Ar*)C-CH-CH-*C*-OH), 151.9, 151.8 (2C, 2xS-CH_2-(*Ar*)C-CH-CH-*C*-C(CH_3)$_3$), 137.0, 136.7 (2C, 2xS-CH_2-(*Ar*)*C*-CH-CH-C-C(CH_3)$_3$), 133.4 (2C, 2xS-(*Ar*)C-*C*H-CH-C-OH), 129.3, 129.1 (4C, 4xS-CH_2-(*Ar*)C-*C*H-CH-C-

C$(CH_3)_3$), 125.7, 125.6 (4C, 4xS-CH_2-(*Ar*)C-CH-$\underline{C}$H-C-C$(CH_3)_3$), 124.1 (1C, S-(*Ar*)$\underline{C}$-CH-CH-C-OH), 117.3 (2C, 2xS-(*Ar*)C-CH-$\underline{C}$H-C-OH), 107.5 (2C, 2xCH_3-(*Ar*)C-CH-CH-$\underline{C}$), 100.2 (2C, 2xCH_3-(*Ar*)$\underline{C}$-CH-CH-C), 84.1 (2C, 2xCH_3-(*Ar*)C-$\underline{C}$H-CH-C), 83.6 (2C, 2xCH_3-(*Ar*)C-CH-$\underline{C}$H-C), 83.5 (2C, 2xCH_3-(*Ar*)C-CH-$\underline{C}$H-C), 82.2 (2C, 2xCH_3-(*Ar*)C-$\underline{C}$H-CH-C), 39.9 (1C, S-$\underline{C}H_2$-(*Ar*)C-CH-CH-C-C$(CH_3)_3$), 39.2 (1C, S-$\underline{C}H_2$-(*Ar*)C-CH-CH-C-C$(CH_3)_3$), 34.91 (1C, S-CH_2-(*Ar*)C-CH-CH-C-$\underline{C}(CH_3)_3$), 34.88 (1C, S-CH_2-(*Ar*)C-CH-CH-C-$\underline{C}(CH_3)_3$), 31.55 (3C, S-CH_2-(*Ar*)C-CH-CH-C-C$(\underline{C}H_3)_3$), 31.54 (3C, S-CH_2-(*Ar*)C-CH-CH-C-C$(\underline{C}H_3)_3$), 31.0 (2C, 2x(*Ar*)CH-CH-C-$\underline{C}H(CH_3)_2$), 23.2 (2C, (*Ar*)CH-CH-C-CH$(\underline{C}H_3)_2$), 22.9 (2C, (*Ar*)CH-CH-C-CH$(\underline{C}H_3)_2$), 18.1 (2C, 2x$\underline{C}H_3$-(*Ar*)C-CH-CH).

ESI-MS(+): *m/z* found 955.2102 $[M-Cl]^+$, calcd. for $C_{48}H_{63}ORu_2S_3^+$ 955.2123, the isotopic pattern corresponds well to the calculated one.

R$_f$ (CH_2Cl_2/CH_3OH 10:1 (v/v)) = 0.47.

Elemental analysis (%): calcd. for $C_{48}H_{63}ClORu_2S_3\cdot 2CH_3OH$: C 56.98, H 6.79; found C 56.91, H 6.78.

Synthesis of $[(\eta^6\text{-}p\text{-}MeC_6H_4Pr^i)_2Ru_2(\mu_2\text{-}SCH_2C_6H_4\text{-}p\text{-}Bu^t)_2(\mu_2\text{-}SC_6H_4\text{-}p\text{-}NH_2)]Cl$ (4)

The mixed amino compound **4** was prepared and purified by adapting literature procedures[6]. To a solution of dithiolato precursor **1** (1.998 g, 2.221 mmol, 1 equiv.) in EtOH (140 mL) was added dropwise at reflux under inert atmosphere (N_2) a solution of 4-amino-benzenethiol (0.417 g, 3.393 mmol, 1.5 equiv.) in EtOH (30 mL). The reaction mixture was further refluxed overnight and the reaction evolution was verified by TLC. The reaction mixture was cooled to r.t. and concentrated. Purification by column chromatography using CH_2Cl_2/CH_3OH (9:1, v/v) afforded **4** as an orange solid (1.85 g, 1.871 mmol, yield 84%).

^{1}H-NMR ($CDCl_3$) δ_H, ppm: 7.39-7.52 (10H, m, 2xS-(*Ar*)C-C$\underline{H}$-CH-C-NH_2, 4xS-CH_2-(*Ar*)C-C$\underline{H}$-CH-C-C$(CH_3)_3$, 4xS-CH_2-(*Ar*)C-CH-C$\underline{H}$-C-C$(CH_3)_3$), 6.75 (2H, d, 2xS-(*Ar*)C-CH-C$\underline{H}$-C-NH_2, $^3J_{H,H}$ = 8.5 Hz), 5.01 (2H, d, 2xCH_3-(*Ar*)C-CH-C$\underline{H}$-C, $^3J_{H,H}$ = 5.7 Hz), 4.90 (2H, d, 2xCH_3-(*Ar*)C-CH-C$\underline{H}$-C, $^3J_{H,H}$ = 5.7 Hz), 4.74 (2H, d, 2xCH_3-(*Ar*)C-C$\underline{H}$-CH-C, $^3J_{H,H}$ = 5.7 Hz), 4.58 (2H, d, 2xCH_3-(*Ar*)C-C$\underline{H}$-CH-C, $^3J_{H,H}$ = 5.7 Hz), 3.54 (2H, s, S-C$\underline{H_2}$-(*Ar*)C-CH-CH-C-C$(CH_3)_3$), 3.37 (2H, s, S-C$\underline{H_2}$-(*Ar*)C-CH-CH-C-C$(CH_3)_3$), 1.97 (2H, sept, 2x(*Ar*)C-CH-CH-C-C$\underline{H}(CH_3)_2$, $^3J_{H,H}$ = 6.9 Hz), 1.71 (6H, s, 2xC$\underline{H_3}$-(*Ar*)C-CH-CH-C), 1.36 (9H, s, S-CH_2-(*Ar*)C-CH-CH-C-C$(C\underline{H_3})_3$), 1.34 (9H, s, S-CH_2-(*Ar*)C-CH-CH-C-C$(C\underline{H_3})_3$), 0.98 (6H, d, 2x(*Ar*)C-CH-CH-C-CH$(C\underline{H_3})_2$, $^3J_{H,H}$ = 6.9 Hz), 0.93 (6H, d, 2x(*Ar*)C-CH-CH-C-CH$(C\underline{H_3})_2$, $^3J_{H,H}$ = 6.9 Hz).

^{13}C-NMR ($CDCl_3$) δ_C, ppm: 151.85, 151.77 (2C, 2xS-CH_2-(*Ar*)C-CH-CH-$\underline{C}$-C$(CH_3)_3$), 148.8 (1C, S-(*Ar*)C-CH-CH-$\underline{C}$-NH_2), 137.0, 136.8 (2C, 2xS-CH_2-(*Ar*)$\underline{C}$-CH-CH-C-C$(CH_3)_3$), 133.6 (2C, 2xS-(*Ar*)C-$\underline{C}$H-CH-C-NH_2), 129.3, 129.1 (4C, 4xS-CH_2-(*Ar*)C-$\underline{C}$H-CH-C-C$(CH_3)_3$), 125.7, 125.5 (4C, 4xS-CH_2-(*Ar*)C-CH-$\underline{C}$H-C-C$(CH_3)_3$), 122.9 (1C, S-

(*Ar*)$\underline{C}$-CH-CH-C-NH_2), 115.6 (2C, 2xS-(*Ar*)C-CH-$\underline{C}$H-C-NH_2), 107.3 (2C, 2xCH_3-(*Ar*)C-CH-CH-$\underline{C}$), 100.2 (2C, 2xCH_3-(*Ar*)$\underline{C}$-CH-CH-C), 84.1 (2C, 2xCH_3-(*Ar*)C-CH-$\underline{C}$H-C), 83.64 (2C, 2xCH_3-(*Ar*)C-$\underline{C}$H-CH-C), 83.56 (2C, 2xCH_3-(*Ar*)C-CH-$\underline{C}$H-C), 82.2 (2C, 2xCH_3-(*Ar*)C-$\underline{C}$H-CH-C), 39.9 (1C, S-$\underline{C}H_2$-(*Ar*)C-CH-CH-C-C$(CH_3)_3$), 39.2 (1C, S-$\underline{C}H_2$-(*Ar*)C-CH-CH-C-C$(CH_3)_3$), 34.91 (1C, S-CH_2-(*Ar*)C-CH-CH-C-$\underline{C}(CH_3)_3$), 34.88 (1C, S-CH_2-(*Ar*)C-CH-CH-C-$\underline{C}(CH_3)_3$), 31.6 (6C, 2xS-CH_2-(*Ar*)C-CH-CH-C-C$(\underline{C}H_3)_3$), 31.0 (2C, 2x(*Ar*)CH-CH-C-$\underline{C}H(CH_3)_2$), 23.2 (2C, (*Ar*)CH-CH-C-CH$(\underline{C}H_3)_2$), 22.9 (2C, (*Ar*)CH-CH-C-CH$(\underline{C}H_3)_2$), 18.2 (2C, 2x$\underline{C}H_3$-(*Ar*)C-CH-CH).

ESI-MS(+): *m/z* found 954.2291 $[M\text{-}Cl]^+$, calcd. for $C_{48}H_{64}NRu_2S_3^+$ 954.2282, the isotopic pattern corresponds well to the calculated one.

R$_f$ (CH_2Cl_2/CH_3OH 10:1 (v/v)) = 0.52.

Elemental analysis (%): calcd. for $C_{48}H_{64}ClNRu_2S_3\cdot 3CH_3OH$: C 56.46, H 7.06, N 1.29; found C 56.39, H 7.09, N 1.62.

Synthesis of $[(\eta^6\text{-}p\text{-}MeC_6H_4Pr^i)_2Ru_2(\mu_2\text{-}SCH_2C_6H_4\text{-}p\text{-}Bu^t)(\mu_2\text{-}SC_6H_4\text{-}p\text{-}OH)_2]Cl$ (5)

The mixed di-hydroxy compound **5** was prepared and purified according to literature procedure[2]. To a solution of dihydroxy dithiolato precursor **2** (2.00 g, 2.22 mmol, 1 equiv.) in EtOH (140 mL) was added dropwise at reflux under inert atmosphere (N_2) a solution of 4-*tert*-butylbenzenemethanethiol (0.809 g, 6.66 mmol, 3 equiv.) in EtOH (10 mL). The reaction mixture was further refluxed overnight and the reaction evolution was verified by TLC. The reaction mixture was cooled to r.t. and concentrated under reduced pressure. Purification by column chromatography using CH_2Cl_2/CH_3OH (9:1, v/v) afforded **5** as an orange solid (2.10 g, 2.132 mmol, yield 96%).

^{1}H-NMR ($CDCl_3$) δ_H, ppm: 9.68 (2H, s br, 2xO$\underline{H}$), 7.65 (2H, d, 2xS-(*Ar*)C-C$\underline{H}$-CH-C-OH, $^3J_{H,H}$ = 8.6 Hz), 7.50 (2H, d, 2xS-(*Ar*)C-C$\underline{H}$-CH-C-OH, $^3J_{H,H}$ = 8.6 Hz), 7.41-7.46 (4H, m, 2xS-CH_2-(*Ar*)C-C$\underline{H}$-CH-C-C$(CH_3)_3$, 2xS-CH_2-(*Ar*)C-CH-C$\underline{H}$-C-C$(CH_3)_3$, $^3J_{H,H}$ = 8.7 Hz), 7.20 (2H, d, 2xS-(*Ar*)C-CH-C$\underline{H}$-C-OH, $^3J_{H,H}$ = 8.6 Hz), 7.15 (2H, d, 2xS-(*Ar*)C-CH-C$\underline{H}$-C-OH, $^3J_{H,H}$ = 8.6 Hz), 4.98 (2H, d, 2xCH_3-(*Ar*)C-CH-C$\underline{H}$-C, $^3J_{H,H}$ = 5.8 Hz), 4.89 (2H, d, 2xCH_3-(*Ar*)C-CH-C$\underline{H}$-C, $^3J_{H,H}$ = 5.5 Hz), 4.88 (2H, d, 2xCH_3-(*Ar*)C-C$\underline{H}$-CH-C, $^3J_{H,H}$ = 5.5 Hz), 4.81 (2H, d, 2xCH_3-(*Ar*)C-C$\underline{H}$-CH-C, $^3J_{H,H}$ = 5.8 Hz), 3.51 (2H, s, S-C$\underline{H_2}$-(*Ar*)C-CH-CH-C-C$(CH_3)_3$), 1.95 (2H, sept, 2x(*Ar*)C-CH-CH-C-C$\underline{H}(CH_3)_2$, $^3J_{H,H}$ = 6.9 Hz), 1.64 (6H, s, 2xC$\underline{H_3}$-(*Ar*)C-CH-CH-C), 1.34 (9H, s, S-CH_2-(*Ar*)C-CH-CH-C-C$(C\underline{H_3})_3$), 0.92 (12H, d, 2x(*Ar*)C-CH-CH-C-CH$(C\underline{H_3})_2$, $^3J_{H,H}$ = 6.9 Hz).

^{13}C-NMR ($CDCl_3$) δ_C, ppm: 159.4 (1C, S-(*Ar*)C-CH-CH-$\underline{C}$-OH), 159.0 (1C, S-(*Ar*)C-CH-CH-$\underline{C}$-OH), 151.7 (1C, S-CH_2-(*Ar*)C-CH-CH-$\underline{C}$-C$(CH_3)_3$), 137.0 (1C, S-CH_2-(*Ar*)$\underline{C}$-CH-CH-C-C$(CH_3)_3$), 133.6 (2C, 2xS-(*Ar*)C-$\underline{C}$H-CH-C-OH), 133.5 (2C, 2xS-(*Ar*)C-$\underline{C}$H-CH-C-OH), 129.4 (2C, 2xS-CH_2-(*Ar*)C-$\underline{C}$H-CH-C-C$(CH_3)_3$), 126.4 (2C, 2xS-(*Ar*)$\underline{C}$-CH-CH-C-

OH), 125.6 (2C, 2xS-CH$_2$-(*Ar*)C-CH-$\underline{C}$H-C-C(CH$_3$)$_3$), 117.1 (2C, 2xS-(*Ar*)C-CH-$\underline{C}$H-C-OH), 116.9 (2C, 2xS-(*Ar*)C-CH-$\underline{C}$H-C-OH), 107.4 (2C, 2xCH$_3$-(*Ar*)C-CH-CH-$\underline{C}$), 99.6 (2C, 2xCH$_3$-(*Ar*)$\underline{C}$-CH-CH-C), 84.1 (2C, 2xCH$_3$-(*Ar*)C-CH-$\underline{C}$H-C), 83.6 (2C, 2xCH$_3$-(*Ar*)C-$\underline{C}$H-CH-C), 83.5 (2C, 2xCH$_3$-(*Ar*)C-CH-$\underline{C}$H-C), 82.2 (2C, 2xCH$_3$-(*Ar*)C-$\underline{C}$H-CH-C), 38.4 (1C, S-$\underline{C}$H$_2$-(*Ar*)C-CH-CH-C-C(CH$_3$)$_3$), 34.9 (1C, S-CH$_2$-(*Ar*)C-CH-CH-C-$\underline{C}$(CH$_3$)$_3$), 31.6 (3C, S-CH$_2$-(*Ar*)C-CH-CH-C-C($\underline{C}$H$_3$)$_3$), 29.8 (2C, 2x(*Ar*)CH-CH-C-$\underline{C}$H(CH$_3$)$_2$), 22.9 (2C, (*Ar*)CH-CH-C-CH($\underline{C}$H$_3$)$_2$), 22.7 (2C, (*Ar*)CH-CH-C-CH($\underline{C}$H$_3$)$_2$), 18.0 (2C, 2x$\underline{C}$H$_3$-(*Ar*)C-CH-CH).

ESI-MS(+): *m/z* found 901.1282 [M-Cl]$^+$, calcd. for $C_{43}H_{53}O_2Ru_2S_3^+$ 901.1289, the isotopic pattern corresponds well to the calculated one.

R$_f$ (CH_2Cl_2/CH_3OH 10:1 (v/v)) = 0.42.

Elemental analysis (%): calcd. for $C_{43}H_{53}ClO_2Ru_2S_3\cdot 2CH_3OH$ C 54.06, H 6.15; found C 54.17, H 6.54.

Synthesis of [(η^6-p-MeC$_6$H$_4$Pr^i)$_2$Ru$_2$(μ$_2$-SCH$_2$C$_6$H$_4$-p-Bu^t)$_2$((μ$_2$-SC$_6$H$_4$-p-O)-(CO)(CH$_2$)$_3$(CO)-OH)]Cl (19)

To a solution of **3** (0.201 g, 0.203 mmol, 1 equiv.) in 15 mL dry CH_2Cl_2 at 0°C under inert atmosphere (N_2) were successively added a solution of glutaric anhydride (0.038 g, 0.317 mmol,1.57 equiv.) and TEA (0.15 mL, 1.015 mmol, 5 equiv.). The reaction mixture was then allowed to warm to r.t. and further stirred overnight. The reaction evolution was verified by ^{1}H-NMR and TLC and then the mixture was concentrated under reduced pressure. Purification by column chromatography using CH_2Cl_2/CH_3OH (9:1, v/v)) allowed the isolation of **19** as an orange solid (0.051 g, 0.052 mmol, yield 26%).

^{1}H-NMR (CDCl$_3$) (δ$_H$, ppm): 7.75 (2H, d, 2xS-(*Ar*)C-C$\underline{H}$-CH-C-O, $^3J_{H,H}$ = 8.2 Hz), 7.32-7.50 (8H, m, 4xS-CH$_2$-(*Ar*)C-C$\underline{H}$-CH-C-C(CH$_3$)$_3$, 4xS-CH$_2$-(*Ar*)C-CH-C$\underline{H}$-C-C(CH$_3$)$_3$), 7.11 (2H, d, 2xS-(*Ar*)C-CH-C$\underline{H}$-C-O, $^3J_{H,H}$ = 8.2 Hz), 5.07 (2H, d, 2xCH$_3$-(*Ar*)C-CH-C$\underline{H}$-C, $^3J_{H,H}$ = 5.3 Hz), 4.93 (2H, d, 2xCH$_3$-(*Ar*)C-CH-C$\underline{H}$-C, $^3J_{H,H}$ = 5.6 Hz), 4.87 (2H, d, 2xCH$_3$-(*Ar*)C-C$\underline{H}$-CH-C, $^3J_{H,H}$ = 5.5 Hz), 4.58 (2H, d, 2xCH$_3$-(*Ar*)C-C$\underline{H}$-CH-C, $^3J_{H,H}$ = 5.6 Hz), 3.56 (2H, s, 2xS-C$\underline{H_2}$-(*Ar*)C-CH-CH-C-C(CH$_3$)$_3$), 3.37 (2H, s, 2xS-C$\underline{H_2}$-(*Ar*)C-CH-CH-C-C(CH$_3$)$_3$), 2.70 (2H, t, S-(*Ar*)C-CH-CH-C-O-(C=O)-C$\underline{H_2}$, $^3J_{H,H}$ = 7.1 Hz), 2.56 (2H, t, S-(*Ar*)C-CH-CH-C-O-(C=O)-(CH$_2$)$_2$-C$\underline{H_2}$, $^3J_{H,H}$ = 6.9 Hz), 2.07 (2H, quint, S-(*Ar*)C-CH-CH-C-O-(C=O)-CH$_2$-C$\underline{H_2}$, $^3J_{H,H}$ = 7.2 Hz), 1.89 (2H, sept, 2x(*Ar*)C-CH-CH-C-C$\underline{H}$(CH$_3$)$_2$, $^3J_{H,H}$ = 6.8 Hz), 1.72 (6H, s, 2xC$\underline{H_3}$-(*Ar*)C-CH-CH-C), 1.33 (9H, s, S-CH$_2$-(*Ar*)C-CH-CH-C-C(C$\underline{H_3}$)$_3$), 1.29 (9H, s, S-CH$_2$-(*Ar*)C-CH-CH-C-C(C$\underline{H_3}$)$_3$), 0.92 (6H, d, (*Ar*)C-CH-CH-C-CH(C$\underline{H_3}$)$_2$, $^3J_{H,H}$ = 6.8 Hz), 0.87 (6H, d, (*Ar*)C-CH-CH-C-CH(C$\underline{H_3}$)$_2$, $^3J_{H,H}$ = 6.8 Hz).

ESI-MS(+): *m/z* found 1069.2434 [M-Cl]$^+$, calcd. for $C_{53}H_{69}O_4Ru_2S_3^+$ 1069.2439, the isotopic pattern corresponds well to the calculated one.

5. Tripodal core intermediates

Synthesis of (nitrile-tris(ethane-2,1-diyl)-tris(4-hydroxybutanoate)) (21)

To a solution of triethanolamine (0.300 g, 2.010 mmol, 1 equiv.) in dry CH_2Cl_2 (50 mL) under inert atmosphere (N_2) was added glutaric anhydride (0.825 g, 2.039 mmol, 3.6 equiv.), then the reaction mixture was cooled to 0°C and TEA (1.1 mL, 8.04mmol, 4 equiv.) was added dropwise. The reaction mixture was allowed to warm to r.t. and was further stirred for further 19 h. The reaction mixture was concentrated, the crude was washed with Et_2O (2x20 mL) and hexane (20 mL). The product was resolubilized in CH_2Cl_2 (50 mL) and concentrated under reduced pressure to afford **21** as a colourless viscous oil in quantitative yield. Note that the consistency of **21** (oil) precluded obtaining elemental analysis data.

^{1}H-NMR ($CDCl_3$) δ_H, ppm: 4.12 (6H, t, 3xN-CH_2-C$\underline{H_2}$, $^3J_{H,H}$ = 5.7 Hz), 2.83 (6H, t, 3xN-C$\underline{H_2}$, $^3J_{H,H}$ = 5.7 Hz), 2.38 (6H, m, 3xCH_2-O-(C=O)-C$\underline{H_2}$, $^3J_{H,H}$ = 7.3 Hz), 2.33 (6H, m, 3xCH_2-O-(C=O)-$(CH_2)_2$-C$\underline{H_2}$, $^3J_{H,H}$ = 7.2 Hz), 1.91-1.96 (6H, m, 3xCH_2-O-(C=O)-CH_2-C$\underline{H_2}$, $^3J_{H,H}$ = 7.2 Hz).

^{13}C-NMR ($CDCl_3$) δ_C, ppm: 177.7 (3C, 3xCH_2-O-(C=O)-$(CH_2)_3$-($\underline{C}$=O)-OH), 173.3 (3C, 3xCH_2-O-($\underline{C}$=O)-$(CH_2)_3$), 62.7 (3C, 3xN-CH_2-$\underline{C}H_2$), 53.5 (3C, 3xN-$\underline{C}H_2$), 34.5 (3C, 3xCH_2-O-(C=O)-$\underline{C}H_2$), 33.7 (3C, 3xCH_2-O-(C=O)-$(CH_2)_2$-$\underline{C}H_2$), 20.9 (3C, 3xCH_2-O-(C=O)-CH_2-$\underline{C}H_2$).

ESI-MS(-): *m/z* found 490.1927 $[M-H]^-$, calcd. for $C_{24}H_{30}O_{12}$ 491.2003.

Synthesis of (4,4',4''-((benzene-1,3,5-triyl-tris(methylene))tris(oxy))tris(4-oxobutanoic acid)) (22)

To a solution of benzene-1,3,5-triyltrimethanol (0.300 g, 1.783 mmol, 1 equiv.) in dry CH_2Cl_2 (50 mL) under inert atmosphere (N_2) was added glutaric anhydride (0.732 g, 6.421 mmol, 3.6 equiv.) and then the reaction mixture was cooled to 0°C and TEA (1 mL, 7.1 mmol, 4 equiv.) was added dropwise. The reaction mixture was allowed to warm to r.t. and was stirred for further 22 h. The reaction mixture was concentrated, the crude was washed with Et_2O (20 mL). The product was resolubilized in CH_2Cl_2 (50 mL) and concentrated under reduced pressure to afford **22** as a colourless viscous oil in quantitative yield. Note that the consistency of **22** (oil) precluded obtaining elemental analysis data.

^{1}H-NMR ($CDCl_3$) δ_H, ppm: 7.26 (3H, s, 3x(*Ar*)C-C$\underline{H}$), 5.09 (6H, s, 3x(*Ar*)C-C$\underline{H_2}$-O-(C=O)), 2.42 (6H, t, 3xCH_2-O-(C=O)-C$\underline{H_2}$, $^3J_{H,H}$ = 7.4 Hz), 2.30 (6H, t, 3xCH_2-O-(C=O)-$(CH_2)_2$-C$\underline{H_2}$, $^3J_{H,H}$ = 7.2 Hz), 1.93 (6H, m, 3xCH_2-O-(C=O)-CH_2-C$\underline{H_2}$, $^3J_{H,H}$ = 7.4 Hz).

^{13}C-NMR ($CDCl_3$) δ_C, ppm: 177.6 (3C, 3xCH_2-($\underline{C}$=O)-OH), 173.2 (3C, 3xCH_2-O-($\underline{C}$=O)-CH_2), 137.1 (3C, 3x(*Ar*)$\underline{C}$-CH_2-O), 127.3 (3C, 3x(*Ar*)C-$\underline{C}$H), 65.7 (6C, 3x$\underline{C}H_2$-O-(C=O)),

34.6 (6C, $3xCH_2$-O-(C=O)-$\underline{C}H_2$), 33.8 (6C, $3xCH_2$-O-(C=O)-$(CH_2)_2$-$\underline{C}H_2$), 20.9 (6C, $3xCH_2$-O-(C=O)-CH_2-$\underline{C}H_2$).

ESI-MS(+): *m/z* found 533.1619 $[M+Na]^+$, calcd. for $C_{24}H_{30}O_{12}$ 510.1737.

6. Compounds with two trithiolato-bridged ruthenium(II)-*p*-cymene units and alkyl diester linkers

Synthesis of $[(\eta^6\text{-}p\text{-}MeC_6H_4Pr^i)_2Ru_2(\mu_2\text{-}SCH_2C_6H_4\text{-}p\text{-}Bu^t)_2(\mu_2\text{-}SC_6H_4\text{-}p\text{-}O)\text{-}(CO)(CH_2)_2(CO)\text{-}(p\text{-}O\text{-}C_6H_4\text{-}\mu_2S)Ru_2(\mu_2\text{-}SCH_2C_6H_4\text{-}p\text{-}Bu^t)_2(\eta^6\text{-}p\text{-}MeC_6H_4Pr^i)_2]Cl_2$ (6)

To a solution of **3** (0.200 g, 0.202 mmol, 1 equiv.) in 20 mL dry CH_2Cl_2 at 0°C under inert atmosphere (N_2) were added successively a solution of succinyl dichloride (0.016 g, 0.101 mmol, 0.5 equiv.) in dry CH_2Cl_2 (5.8 mL) and TEA (0.1 mL, 0.727 mmol, 3.6 equiv). The reaction mixture was allowed to warm to r.t. and was further stirred overnight. The reaction evolution was verified by ^1H-NMR and TLC and the reaction mixture was concentrated under reduced pressure. Purification by column chromatography using CH_2Cl_2/CH_3OH (9:1, v/v) allowed the isolation of **6** as an orange solid (0.048 g, 0.024 mmol, yield 23%).

^{1}H-NMR ($CDCl_3$) (δ_H, ppm): 7.84 (4H, d, 4xS-(*Ar*)C-C$\underline{H}$-CH-C-O, $^3J_{H,H}$ = 8.6 Hz), 7.40-7.49 (16H, m, 8xS-CH_2-(*Ar*)C-C$\underline{H}$-CH-C-$C(CH_3)_3$, 8xS-CH_2-(*Ar*)C-CH-C$\underline{H}$-C-$C(CH_3)_3$), 7.12 (4H, d, 4xS-(*Ar*)C-CH-C$\underline{H}$-C-O, $^3J_{H,H}$ = 8.6 Hz), 5.17 (4H, d, 4xCH_3-(*Ar*)C-CH-C$\underline{H}$-C, $^3J_{H,H}$ = 5.7 Hz), 5.02 (4H, d, 4xCH_3-(*Ar*)C-CH-C$\underline{H}$-C, $^3J_{H,H}$ = 5.8 Hz), 4.96 (4H, d, 4xCH_3-(*Ar*)C-C$\underline{H}$-CH-C, $^3J_{H,H}$ = 5.7 Hz), 4.62 (4H, d, 4xCH_3-(*Ar*)C-C$\underline{H}$-CH-C, $^3J_{H,H}$ = 5.8 Hz), 3.60 (4H, s, 2xS-C$\underline{H_2}$-(*Ar*)C-CH-CH-C-$C(CH_3)_3$), 3.43 (4H, s, 2xS-C$\underline{H_2}$-(*Ar*)C-CH-CH-C-$C(CH_3)_3$), 3.04 (4H, s, 2xS-(*Ar*)C-CH-CH-C-O-(C=O)-C$\underline{H_2}$), 1.91 (4H, sept, 4x(*Ar*)C-CH-CH-C-C$\underline{H}$$(CH_3)_2$, $^3J_{H,H}$ = 6.9 Hz), 1.77 (12H, s, 4xC$\underline{H_3}$-(*Ar*)C-CH-CH-C), 1.36 (18H, s, 2xS-CH_2-(*Ar*)C-CH-CH-C-C(C$\underline{H_3}$)$_3$), 1.32 (18H, s, 2xS-CH_2-(*Ar*)C-CH-CH-C-C(C$\underline{H_3}$)$_3$), 0.95 (12H, d, 2x(*Ar*)C-CH-CH-C-CH(C$\underline{H_3}$)$_2$, $^3J_{H,H}$ = 6.9 Hz), 0.90 (12H, d, 2x(*Ar*)C-CH-CH-C-CH(C$\underline{H_3}$)$_2$, $^3J_{H,H}$ = 6.9 Hz).

^{13}C-NMR ($CDCl_3$) (δ_C, ppm): 170.6 (2C, 2xS-(*Ar*)C-CH-CH-C-O-($\underline{C}$=O)), 151.8, 151.9 (4C, 4xS-CH_2-(*Ar*)C-CH-CH-$\underline{C}$-$C(CH_3)_3$), 151.1 (2C, 2xS-(*Ar*)C-CH-CH-$\underline{C}$-O-(C=O)), 136.8, 136.9 (4C, 4xS-CH_2-(*Ar*)$\underline{C}$-CH-CH-C-$C(CH_3)_3$), 135.3 (2C, 2xS-(*Ar*)$\underline{C}$-CH-CH-C-O-(C=O)), 133.9 (4C, 4xS-(*Ar*)C-$\underline{C}$H-CH-C-O-(C=O)), 129.3, 129.4 (8C, 8xS-CH_2-(*Ar*)C-$\underline{C}$H-CH-C), 125.5, 125.7 (8C, 8xS-CH_2-(*Ar*)C-CH-$\underline{C}$H-C), 122.4 (4C, 4xS-(*Ar*)C-CH-$\underline{C}$H-C-O-(C=O)), 107.1 (4C, 4xCH_3-(*Ar*)C-CH-CH-$\underline{C}$), 100.7 (4C, 4xCH_3-(*Ar*)$\underline{C}$-CH-CH-C), 84.5 (4C, 4xCH_3-(*Ar*)C-CH-$\underline{C}$H-C), 83.83 (4C, 4xCH_3-(*Ar*)C-$\underline{C}$H-CH-C), 83.79 (4C, 4xCH_3-(*Ar*)C-CH-$\underline{C}$H-C), 82.7 (4C, 4xCH_3-(*Ar*)C-$\underline{C}$H-CH-C), 40.1 (2C, 2xS-$\underline{C}H_2$-(*Ar*)C-CH-CH-C-$C(CH_3)_3$), 39.7 (2C, 2xS-$\underline{C}H_2$-(*Ar*)C-CH-CH-C-$C(CH_3)_3$), 34.94 (2C, 2xS-CH_2-(*Ar*)C-CH-CH-C-$\underline{C}(CH_3)_3$), 34.89 (2C, 2xS-CH_2-(*Ar*)C-CH-CH-C-$\underline{C}(CH_3)_3$), 31.6 (12C, 4xS-CH_2-(*Ar*)C-CH-CH-C-C($\underline{C}H_3$)$_3$), 31.0 (4C, 4x(*Ar*)CH-CH-C-$\underline{C}H(CH_3)_2$), 29.5 (2C, 2xS-CH_2-

(*Ar*)C-CH-CH-C-O-(C=O)-$\underline{C}H_2$), 23.3 (4C, 2x(*Ar*)CH-CH-C-CH($\underline{C}H_3$)$_2$), 22.8 (4C, 2x(*Ar*)CH-CH-C-CH($\underline{C}H_3$)$_2$), 18.3 (4C, 4x$\underline{C}H_3$-(*Ar*)C-CH-CH-C).

ESI-MS(+): *m/z* 995.7163 $[M\text{-}2Cl]^{2+}$, calcd. for $C_{100}H_{128}O_4Ru_4S_6^{2+}$ 995.3795, the isotopic pattern corresponds well to the calculated one.

R$_f$ (CH_2Cl_2/CH_3OH 9:1 (v/v)) = 0.39.

Elemental analysis (%): calcd. for $C_{100}H_{128}Cl_2O_4Ru_4S_6\cdot 2CH_3OH\cdot H_2O$: C 57.15, H 6.49; found: C 57.10, H 6.48.

Synthesis of [(η^6-p-MeC$_6$H$_4$Pr^i)$_2$Ru$_2$(μ_2-SCH$_2$C$_6$H$_4$-p-Bu^t)$_2$(μ_2-SC$_6$H$_4$-p-O)-(CO)(CH$_2$)$_3$(CO)-(p-O-C$_6$H$_4$-μ_2S)Ru$_2$(μ_2-SCH$_2$C$_6$H$_4$-p-Bu^t)$_2$(η^6-p-MeC$_6$H$_4$Pr^i)$_2$]Cl$_2$ (7)

To a solution of **3** (0.201 g, 0.203 mmol, 1 equiv.) in 20 mL dry CH_2Cl_2 at 0°C under inert atmosphere (N_2) were added successively a solution of glutaroyl dichloride (0.018 g, 0.102 mmol, 0.51 equiv.) in dry CH_2Cl_2 (5 mL) and TEA (0.1 mL, 0.727 mmol, 3.6 equiv.). The reaction mixture was then allowed to warm to r.t. and further stirred overnight. The reaction evolution was verified by ^{1}H-NMR and TLC, and then concentrated under reduced pressure. Purification by column chromatography using CH_2Cl_2/CH_3OH (9:1, v/v) allowed the isolation of **7** as an orange solid (0.106 g, 0.051 mmol, yield 50%).

^{1}H-NMR (CDCl$_3$) (δ_H, ppm): 7.83 (4H, d, 4xS-(*Ar*)C-C*$\underline{H}$*-CH-C-O, $^3J_{H,H}$ = 8.6 Hz), 7.40-7.51 (16H, m, 8xS-CH_2-(*Ar*)C-C*$\underline{H}$*-CH-C-C(CH_3)$_3$), 8xS-CH_2-(*Ar*)C-CH-C*$\underline{H}$*-C-C(CH_3)$_3$), 7.13 (4H, d, 4xS-(*Ar*)C-CH-C*$\underline{H}$*-C-O, $^3J_{H,H}$ = 8.6 Hz), 5.15 (4H, d, 4xCH_3-(*Ar*)C-CH-C*$\underline{H}$*-C, $^3J_{H,H}$ = 5.8 Hz), 5.01 (4H, d, 4xCH_3-(*Ar*)C-CH-C*$\underline{H}$*-C, $^3J_{H,H}$ = 5.9 Hz), 4.96 (4H, d, 4xCH_3-(*Ar*)C-C*$\underline{H}$*-CH-C, $^3J_{H,H}$ = 5.8 Hz), 4.62 (4H, d, 4xCH_3-(*Ar*)C-C*$\underline{H}$*-CH-C, $^3J_{H,H}$ = 5.8 Hz), 3.61 (4H, s, 2xS-C*$\underline{H_2}$*-(*Ar*)C-CH-CH-C-C(CH_3)$_3$), 3.43 (4H, s, 2xS-C*$\underline{H_2}$*-(*Ar*)C-CH-CH-C-C(CH_3)$_3$), 2.78 (4H, t, 2xS-(*Ar*)C-CH-CH-C-O-(C=O)-C*$\underline{H_2}$*, $^3J_{H,H}$ = 7.2 Hz), 2.22 (2H, quint, S-(*Ar*)C-CH-CH-C-O-(C=O)-CH_2-C*$\underline{H_2}$*, $^3J_{H,H}$ = 7.2 Hz), 1.92 (4H, sept, 4x(*Ar*)C-CH-CH-C-C*$\underline{H}$*(CH_3)$_2$, $^3J_{H,H}$ = 6.9 Hz), 1.77 (12H, s, 4xC*$\underline{H_3}$*-(*Ar*)C-CH-CH-C), 1.37 (18H, s, 2xS-CH_2-(*Ar*)C-CH-CH-C-C(C*$\underline{H_3}$*)$_3$), 1.33 (18H, s, 2xS-CH_2-(*Ar*)C-CH-CH-C-C(C*$\underline{H_3}$*)$_3$), 0.95 (12H, d, 2x(*Ar*)C-CH-CH-C-CH(C*$\underline{H_3}$*)$_2$, $^3J_{H,H}$ = 6.9 Hz), 0.90 (12H, d, 2x(*Ar*)C-CH-CH-C-CH(C*$\underline{H_3}$*)$_2$, $^3J_{H,H}$ = 6.9 Hz).

^{13}C-NMR (CDCl$_3$) (δ_C, ppm): 171.2 (2C, 2xS-(*Ar*)C-CH-CH-C-O-($\underline{C}$=O)), 151.9, 152.0 (4C, 4xS-CH_2-(*Ar*)C-CH-CH-$\underline{C}$-C(CH_3)$_3$), 151.1 (2C, 2xS-(*Ar*)C-CH-CH-$\underline{C}$-O-(C=O)), 136.8 (4C, 4xS-CH_2-(*Ar*)$\underline{C}$-CH-CH-C-C(CH_3)$_3$), 135.1 (2C, 2xS-(*Ar*)$\underline{C}$-CH-CH-C-O-(C=O)), 133.8 (4C, 4xS-(*Ar*)C-$\underline{C}$H-CH-C-O-(C=O)), 129.3, 129.4 (8C, 8xS-CH_2-(*Ar*)C-$\underline{C}$H-CH-C), 125.6, 125.8 (8C, 8xS-CH_2-(*Ar*)C-CH-$\underline{C}$H-C), 122.6 (4C, 4xS-(*Ar*)C-CH-$\underline{C}$H-C-O-(C=O)), 107.2 (4C, 4xCH_3-(*Ar*)C-CH-CH-$\underline{C}$), 100.7 (4C, 4xCH_3-(*Ar*)$\underline{C}$-CH-CH-C), 84.4 (4C, 4xCH_3-(*Ar*)C-CH-$\underline{C}$H-C), 83.84 (4C, 4xCH_3-(*Ar*)C-$\underline{C}$H-CH-C), 83.81 (4C, 4xCH_3-

(*Ar*)C-CH-$\underline{C}$H-C), 82.7 (4C, 4xCH_3-(*Ar*)C-$\underline{C}$H-CH-C), 40.1 (2C, 2xS-$\underline{C}H_2$-(*Ar*)C-CH-CH-C-C(CH_3)$_3$), 39.7 (2C, 2xS-$\underline{C}H_2$-(*Ar*)C-CH-CH-C-C(CH_3)$_3$), 34.95 (2C, 2xS-CH_2-(*Ar*)C-CH-CH-C-$\underline{C}$(CH_3)$_3$), 34.91 (2C, 2xS-CH_2-(*Ar*)C-CH-CH-C-$\underline{C}$(CH_3)$_3$), 33.3 (2C, 2xS-CH_2-(*Ar*)C-CH-CH-C-O-(C=O)-$\underline{C}H_2$), 31.6 (12C, 4xS-CH_2-(*Ar*)C-CH-CH-C-C($\underline{C}H_3$)$_3$), 31.0 (4C, 4x(*Ar*)CH-CH-C-$\underline{C}$H(CH_3)$_2$), 23.3 (4C, 2x(*Ar*)CH-CH-C-CH($\underline{C}H_3$)$_2$), 22.8 (4C, 2x(*Ar*)CH-CH-C-CH($\underline{C}H_3$)$_2$), 20.1 (1C, S-(*Ar*)C-CH-CH-C-O-(C=O)-CH_2-$\underline{C}H_2$), 18.4 (4C, 4x$\underline{C}H_3$-(*Ar*)C-CH-CH-C).

ESI-MS(+): *m/z* found 1002.7290 [M-2Cl]$^{2+}$, calcd. for $C_{101}H_{130}O_4Ru_4S_6^{2+}$ 1002.3930, the isotopic pattern corresponds well to the calculated one.

R$_f$ (CH_2Cl_2/CH_3OH 9:1 (v/v)) = 0.49.

Elemental analysis (%): calcd. for $C_{101}H_{130}Cl_2O_4Ru_4S_6\cdot 0.25CH_2Cl_2\cdot 2CH_3OH$: C 57.39, H 6.46; found: C 57.38, H 6.53.

Synthesis of [(η^6-p-MeC_6H_4Pr^i)$_2$Ru$_2$(μ_2-S$CH_2C_6H_4$-p-Bu^t)$_2$(μ_2-SC_6H_4-p-O)-(CO)(CH_2)$_4$(CO)-(p-O-C_6H_4-μ_2S)Ru$_2$(μ_2-S$CH_2C_6H_4$-p-Bu^t)$_2$(η^6-p-MeC_6H_4Pr^i)$_2$]Cl$_2$ (8)

To a solution of **3** (0.202 g, 0.204 mmol, 1 equiv.) dry CH_2Cl_2 at 0°C under inert atmosphere (N_2) were added successively a solution of adipoyl dichloride (0.019 g, 0.104 mmol, 0.52 equiv.) in dry CH_2Cl_2 (1.4 mL) and TEA (0.1 mL, 0.727 mmol, 3.6 equiv.). The reaction mixture was then allowed to warm to r.t. and further stirred overnight. The reaction evolution was verified by ^{1}H-NMR and TLC and then the mixture was concentrated under reduced pressure. Purification by column chromatography using CH_2Cl_2/CH_3OH (9:1, v/v) allowed the isolation of **8** as an orange solid (0.016 g, 0.008 mmol, yield 8%).

^{1}H-NMR ($CDCl_3$) (δ_H, ppm): 7.83 (4H, d, 4xS-(*Ar*)C-C$\underline{H}$-CH-C-O, $^3J_{H,H}$ = 8.6 Hz), 7.41-7.52 (16H, m, 8xS-CH_2-(*Ar*)C-C$\underline{H}$-CH-C-C(CH_3)$_3$, 8xS-CH_2-(*Ar*)C-CH-C$\underline{H}$-C-C(CH_3)$_3$), 7.10 (4H, d, 4xS-(*Ar*)C-CH-C$\underline{H}$-C-O, $^3J_{H,H}$ = 8.6 Hz), 5.16 (4H, d, 4xCH_3-(*Ar*)C-CH-C$\underline{H}$-C, $^3J_{H,H}$ = 5.7 Hz), 5.01 (4H, d, 4xCH_3-(*Ar*)C-CH-C$\underline{H}$-C, $^3J_{H,H}$ = 5.9 Hz), 4.95 (4H, d, 4xCH_3-(*Ar*)C-C$\underline{H}$-CH-C, $^3J_{H,H}$ = 5.9 Hz), 4.62 (4H, d, 4xCH_3-(*Ar*)C-C$\underline{H}$-CH-C, $^3J_{H,H}$ = 5.9 Hz), 3.61 (4H, s, 2xS-C$\underline{H_2}$-(*Ar*)C-CH-CH-C-C(CH_3)$_3$), 3.43 (4H, s, 2xS-C$\underline{H_2}$-(*Ar*)C-CH-CH-C-C(CH_3)$_3$), 2.66-2.72 (4H, m, 2xS-(*Ar*)C-CH-CH-C-O-(C=O)-C$\underline{H_2}$, $^3J_{H,H}$ = 6.2 Hz), 1.88-1.95 (8H, m, 4x(*Ar*)C-CH-CH-C-C$\underline{H}$(CH_3)$_2$, 2xS-(*Ar*)C-CH-CH-C-O-(C=O)-CH_2-C$\underline{H_2}$, $^3J_{H,H}$ = 6.9 Hz, $^3J_{H,H}$ = 6.2 Hz), 1.77 (12H, s, 4xC$\underline{H_3}$-(*Ar*)C-CH-CH-C), 1.37 (18H, s, 2xS-CH_2-(*Ar*)C-CH-CH-C-C(C$\underline{H_3}$)$_3$), 1.33 (18H, s, 2xS-CH_2-(*Ar*)C-CH-CH-C-C(C$\underline{H_3}$)$_3$), 0.95 (12H, d, 2x(*Ar*)C-CH-CH-C-CH(C$\underline{H_3}$)$_2$, $^3J_{H,H}$ = 6.9 Hz), 0.90 (12H, d, 2x(*Ar*)C-CH-CH-C-CH(C$\underline{H_3}$)$_2$, $^3J_{H,H}$ = 6.9 Hz).

^{13}C-NMR ($CDCl_3$) (δ_C, ppm): 171.6 (2C, 2xS-(*Ar*)C-CH-CH-C-O-($\underline{C}$=O)), 151.8, 152.0 (4C, 4xS-CH_2-(*Ar*)C-CH-CH-$\underline{C}$-C(CH_3)$_3$), 151.2 (2C, 2xS-(*Ar*)C-CH-CH-$\underline{C}$-O-(C=O)), 136.8 (4C, 4xS-CH_2-(*Ar*)$\underline{C}$-CH-CH-C-C(CH_3)$_3$), 135.1 (2C, 2xS-(*Ar*)$\underline{C}$-CH-CH-C-O-

(C=O)), 133.8 (4C, 4xS-(*Ar*)C-$\underline{C}$H-CH-C-O-(C=O)), 129.3, 129.4 (8C, 8xS-CH_2-(*Ar*)C-$\underline{C}$H-CH-C), 125.6, 125.8 (8C, 8xS-CH_2-(*Ar*)C-CH-$\underline{C}$H-C), 122.6 (4C, 4xS-(*Ar*)C-CH-$\underline{C}$H-C-O-(C=O)), 107.2 (4C, 4xCH_3-(*Ar*)C-CH-CH-$\underline{C}$), 100.7 (4C, 4xCH_3-(*Ar*)$\underline{C}$-CH-CH-C), 84.4 (4C, 4xCH_3-(*Ar*)C-CH-$\underline{C}$H-C), 83.8 (8C, 4xCH_3-(*Ar*)C-CH-$\underline{C}$H-C, 4xCH_3-(*Ar*)C-$\underline{C}$H-CH-C), 82.7 (4C, 4xCH_3-(*Ar*)C-$\underline{C}$H-CH-C), 40.1 (2C, 2xS-$\underline{C}H_2$-(*Ar*)C-CH-CH-C-C$(CH_3)_3$), 39.7 (2C, 2xS-$\underline{C}H_2$-(*Ar*)C-CH-CH-C-C$(CH_3)_3$), 34.95 (2C, 2xS-CH_2-(*Ar*)C-CH-CH-C-$\underline{C}(CH_3)_3$), 34.91 (2C, 2xS-CH_2-(*Ar*)C-CH-CH-C-$\underline{C}(CH_3)_3$), 33.2 (2C, 2xS-CH_2-(*Ar*)C-CH-CH-C-O-(C=O)-$\underline{C}H_2$), 31.6 (12C, 4xS-CH_2-(*Ar*)C-CH-CH-C-C$(\underline{C}H_3)_3$), 31.0 (4C, 4x(*Ar*)CH-CH-C-$\underline{C}H(CH_3)_2$), 24.4 (2C, 2xS-(*Ar*)C-CH-CH-C-O-(C=O)-CH_2-$\underline{C}H_2$), 23.3 (4C, 2x(*Ar*)CH-CH-C-CH$(\underline{C}H_3)_2$), 22.8 (4C, 2x(*Ar*)CH-CH-C-CH$(\underline{C}H_3)_2$), 18.3 (4C, 4x$\underline{C}H_3$-(*Ar*)C-CH-CH-C).
ESI-MS(+): *m/z* 1009.7334 $[M-2Cl]^{2+}$, calcd. for $C_{102}H_{132}O_4Ru_4S_6^{2+}$ 1009.4065, the isotopic pattern corresponds well to the calculated one.
R$_f$ (CH_2Cl_2/CH_3OH 9:1 (v/v)) = 0.47.
Elemental analysis (%): calcd. for $C_{102}H_{132}Cl_2O_4Ru_4S_6\cdot 3CH_3OH$: C 57.70, H 6.64; found: C 57.70; H 7.51.

Synthesis of [(η^6-p-MeC$_6$H$_4$Pr^i)$_2$Ru$_2$(μ_2-SCH$_2$C$_6$H$_4$-p-Bu^t)$_2$(μ_2-SC$_6$H$_4$-p-O)-(CO)(CH$_2$)$_5$(CO)-(p-O-C$_6$H$_4$-μ_2S)Ru$_2$(μ_2-SCH$_2$C$_6$H$_4$-p-Bu^t)$_2$(η^6-p-MeC$_6$H$_4$Pr^i)$_2$]Cl$_2$ (9)

To a solution of **3** (0.201 g, 0.203 mmol, 1 equiv.) in 20 mL dry CH_2Cl_2 at 0°C under inert atmosphere (N_2) were added successively a solution of heptanedioyl dichloride (0.020 g, 0.100 mmol, 0.49 equiv.) in dry CH_2Cl_2 (0.83 mL) and TEA (0.1 mL, 0.727 mmol, 3.6 equiv.). The reaction mixture was then allowed to warm to r.t. and further stirred overnight. The reaction evolution was verified by ^{1}H-NMR and TLC and then the mixture was concentrated under reduced pressure. Purification by column chromatography using CH_2Cl_2/CH_3OH (9:1, v/v) allowed the isolation of **9** as an orange solid (0.034 g, 0.016 mmol, yield 16%).
^{1}H-NMR ($CDCl_3$) (δ_H, ppm): 7.80 (4H, d, 4xS-(*Ar*)C-C$\underline{H}$-CH-C-O, $^3J_{H,H}$ = 8.6 Hz), 7.40-7.50 (16H, m, 8xS-CH_2-(*Ar*)C-C$\underline{H}$-CH-C-C$(CH_3)_3$, 8xS-CH_2-(*Ar*)C-CH-C$\underline{H}$-C-C$(CH_3)_3$), 7.08 (4H, d, 4xS-(*Ar*)C-CH-C$\underline{H}$-C-O, $^3J_{H,H}$ = 8.6 Hz), 5.14 (4H, d, 4xCH_3-(*Ar*)C-CH-C$\underline{H}$-C, $^3J_{H,H}$ = 5.7 Hz), 5.00 (4H, d, 4xCH_3-(*Ar*)C-CH-C$\underline{H}$-C, $^3J_{H,H}$ = 5.9 Hz), 4.93 (4H, d, 4xCH_3-(*Ar*)C-C$\underline{H}$-CH-C, $^3J_{H,H}$ = 5.8 Hz), 4.61 (4H, d, 4xCH_3-(*Ar*)C-C$\underline{H}$-CH-C, $^3J_{H,H}$ = 5.9 Hz), 3.60 (4H, s, 2xS-C$\underline{H_2}$-(*Ar*)C-CH-CH-C-C$(CH_3)_3$), 3.42 (4H, s, 2xS-C$\underline{H_2}$-(*Ar*)C-CH-CH-C-C$(CH_3)_3$), 2.64 (4H, t, 2xS-(*Ar*)C-CH-CH-C-O-(C=O)-C$\underline{H_2}$, $^3J_{H,H}$ = 7.4 Hz), 1.90 (4H, sept, 4x(*Ar*)C-CH-CH-C-C$\underline{H}(CH_3)_2$, $^3J_{H,H}$ = 6.9 Hz), 1.49 (4H, quint, 2xS-(*Ar*)C-CH-CH-C-O-(C=O)-CH_2-C$\underline{H_2}$, $^3J_{H,H}$ = 7.4 Hz), 1.53-1.63 (2H, m, S-(*Ar*)C-CH-CH-C-O-(C=O)-$(CH_2)_2$-C$\underline{H_2}$), 1.77 (12H, s, 4xC$\underline{H_3}$-(*Ar*)C-CH-CH-C), 1.36 (18H, s, 2xS-CH_2-(*Ar*)C-CH-CH-C-C(C$\underline{H_3}$)$_3$), 1.32 (18H, s, 2xS-CH_2-(*Ar*)C-CH-CH-C-C(C$\underline{H_3}$)$_3$), 0.94 (12H, d, 2x(*Ar*)C-CH-

CH-C-CH(C*H*$_3$)$_2$, $^3J_{H,H}$ = 6.9 Hz), 0.89 (12H, d, 2x(*Ar*)C-CH-CH-C-CH(C*H*$_3$)$_2$, $^3J_{H,H}$ = 6.9 Hz).

^{13}C-NMR (CDCl$_3$) (δ_C, ppm): 171.8 (2C, 2xS-(*Ar*)C-CH-CH-C-O-(*C*=O)), 151.8, 151.9 (4C, 4xS-CH$_2$-(*Ar*)C-CH-CH-*C*-C(CH$_3$)$_3$), 151.2 (2C, 2xS-(*Ar*)C-CH-CH-*C*-O-(C=O)), 136.8 (4C, 4xS-CH$_2$-(*Ar*)*C*-CH-CH-C-C(CH$_3$)$_3$), 135.0 (2C, 2xS-(*Ar*)*C*-CH-CH-C-O-(C=O)), 133.7 (4C, 4xS-(*Ar*)C-*C*H-CH-C-O-(C=O)), 129.2, 129.4 (8C, 8xS-CH$_2$-(*Ar*)C-*C*H-CH-C), 125.5, 125.7 (8C, 8xS-CH$_2$-(*Ar*)C-CH-*C*H-C), 122.6 (4C, 4xS-(*Ar*)C-CH-*C*H-C-O-(C=O)), 107.2 (4C, 4xCH$_3$-(*Ar*)C-CH-CH-*C*), 100.7 (4C, 4xCH$_3$-(*Ar*)*C*-CH-CH-C), 84.3 (4C, 4xCH$_3$-(*Ar*)C-CH-*C*H-C), 83.8 (8C, 4xCH$_3$-(*Ar*)C-CH-*C*H-C, 4xCH$_3$-(*Ar*)C-*C*H-CH-C), 82.7 (4C, 4xCH$_3$-(*Ar*)C-*C*H-CH-C), 40.1 (2C, 2xS-*C*H$_2$-(*Ar*)C-CH-CH-C-C(CH$_3$)$_3$), 39.6 (2C, 2xS-*C*H$_2$-(*Ar*)C-CH-CH-C-C(CH$_3$)$_3$), 34.92 (2C, 2xS-CH$_2$-(*Ar*)C-CH-CH-C-*C*(CH$_3$)$_3$), 34.88 (2C, 2xS-CH$_2$-(*Ar*)C-CH-CH-C-*C*(CH$_3$)$_3$), 33.2 (2C, 2xS-CH$_2$-(*Ar*)C-CH-CH-C-O-(C=O)-*C*H$_2$), 31.56 (6C, 2xS-CH$_2$-(*Ar*)C-CH-CH-C-C(*C*H$_3$)$_3$), 31.54 (6C, 2xS-CH$_2$-(*Ar*)C-CH-CH-C-C(*C*H$_3$)$_3$), 31.0 (4C, 4x(*Ar*)CH-CH-C-*C*H(CH$_3$)$_2$), 28.6 (1C, S-(*Ar*)C-CH-CH-C-O-(C=O)-(CH$_2$)$_2$-*C*H$_2$), 24.6 (2C, 2xS-(*Ar*)C-CH-CH-C-O-(C=O)-CH$_2$-*C*H$_2$), 23.3 (4C, 2x(*Ar*)CH-CH-C-CH(*C*H$_3$)$_2$), 22.8 (4C, 2x(*Ar*)CH-CH-C-CH(*C*H$_3$)$_2$), 18.3 (4C, 4x*C*H$_3$-(*Ar*)C-CH-CH-C).

ESI-MS(+): *m/z* 1016.7390 [M-2Cl]$^{2+}$, calcd. for $C_{103}H_{134}O_4Ru_4S_6^{2+}$ 1016.4205, the isotopic pattern corresponds well to the calculated one.

R$_f$ (CH$_2$Cl$_2$/CH$_3$OH 9:1 (v/v)) = 0.47.

Elemental analysis (%): calcd. for $C_{103}H_{134}Cl_2O_4Ru_4S_6\cdot 2CH_3OH\cdot H_2O$: C 57.70, H 6.64; found: C 57.77, H 6.61.

Synthesis of [(η^6-p-MeC$_6$H$_4$Pr^i)$_2$Ru$_2$(μ_2-SCH$_2$C$_6$H$_4$-p-Bu^t)$_2$(μ_2-SC$_6$H$_4$-p-O)-(CO)(CH$_2$)$_6$(CO)-(p-O-C$_6$H$_4$-μ_2S)Ru$_2$(μ_2-SCH$_2$C$_6$H$_4$-p-Bu^t)$_2$(η^6-p-MeC$_6$H$_4$Pr^i)$_2$]Cl$_2$ (10)

To a solution of **3** (0.200 g, 0.202 mmol, 1 equiv.) in 20 mL dry CH$_2$Cl$_2$ at 0°C under inert atmosphere (N$_2$) were added successively a solution of octanedioyl dichloride (0.021 g, 0.101 mmol, 0.5 equiv.) in dry CH$_2$Cl$_2$ (2.9 mL) and TEA (0.1 mL, 0.727 mmol, 3.6 equiv.). The reaction mixture was then allowed to warm up to r.t. and further stirred overnight. The reaction evolution was verified by ^{1}H-NMR and TLC and then the mixture was concentrated under reduced pressure. Purification by column chromatography using CH$_2$Cl$_2$/CH$_3$OH (9:1) (v/v) allowed the isolation of **10** as an orange solid (0.014 g, 0.007 mmol, yield 7%).

^{1}H-NMR (CDCl$_3$) (δ_H, ppm): 7.81 (4H, d, 4xS-(*Ar*)C-C*H*-CH-C-O, $^3J_{H,H}$ = 8.7 Hz), 7.40-7.50 (16H, m, 8xS-CH$_2$-(*Ar*)C-C*H*-CH-C-C(CH$_3$)$_3$, 8xS-CH$_2$-(*Ar*)C-CH-C*H*-C-C(CH$_3$)$_3$), 7.07 (4H, d, 4xS-(*Ar*)C-CH-C*H*-C-O, $^3J_{H,H}$ = 8.7 Hz), 5.14 (4H, d, 4xCH$_3$-(*Ar*)C-CH-C*H*-C, $^3J_{H,H}$ = 5.8 Hz), 5.00 (4H, d, 4xCH$_3$-(*Ar*)C-CH-C*H*-C, $^3J_{H,H}$ = 5.9 Hz), 4.94 (4H, d, 4xCH$_3$-(*Ar*)C-C*H*-CH-C, $^3J_{H,H}$ = 5.8 Hz), 4.61 (4H, d, 4xCH$_3$-(*Ar*)C-C*H*-CH-C, $^3J_{H,H}$ = 5.9 Hz), 3.60

(4H, s, 2xS-C$\underline{H_2}$-(*Ar*)C-CH-CH-C-C(CH_3)$_3$), 3.43 (4H, s, 2xS-C$\underline{H_2}$-(*Ar*)C-CH-CH-C-C(CH_3)$_3$), 2.61 (4H, t, 2xS-(*Ar*)C-CH-CH-C-O-(C=O)-C$\underline{H_2}$, $^3J_{H,H}$ = 7.4 Hz), 1.90 (4H, sept, 4x(*Ar*)C-CH-CH-C-C$\underline{H}$(CH_3)$_2$, $^3J_{H,H}$ = 6.9 Hz), 1.76-1.88 (4H, m, 2xS-(*Ar*)C-CH-CH-C-O-(C=O)-CH_2-C$\underline{H_2}$, $^3J_{H,H}$ = 7.4 Hz), 1.77 (12H, s, 4xC$\underline{H_3}$-(*Ar*)C-CH-CH-C), 1.47-1.54 (4H, m, 2xS-(*Ar*)C-CH-CH-C-O-(C=O)-(CH_2)$_2$-C$\underline{H_2}$), 1.36 (18H, s, 2xS-CH_2-(*Ar*)C-CH-CH-C-C(C$\underline{H_3}$)$_3$), 1.32 (18H, s, 2xS-CH_2-(*Ar*)C-CH-CH-C-C(C$\underline{H_3}$)$_3$), 0.95 (12H, d, 2x(*Ar*)C-CH-CH-C-CH(C$\underline{H_3}$)$_2$, $^3J_{H,H}$ = 6.9 Hz), 0.89 (12H, d, 2x(*Ar*)C-CH-CH-C-CH(C$\underline{H_3}$)$_2$, $^3J_{H,H}$ = 6.9 Hz).

^{13}C-NMR ($CDCl_3$) (δ_C, ppm): 172.0 (2C, 2xS-(*Ar*)C-CH-CH-C-O-($\underline{C}$=O)), 151.8, 151.9 (4C, 4xS-CH_2-(*Ar*)C-CH-CH-$\underline{C}$-C(CH_3)$_3$), 151.2 (2C, 2xS-(*Ar*)C-CH-CH-$\underline{C}$-O-(C=O)), 136.8, 137.0 (4C, 4xS-CH_2-(*Ar*)$\underline{C}$-CH-CH-C-C(CH_3)$_3$), 135.0 (2C, 2xS-(*Ar*)$\underline{C}$-CH-CH-C-O-(C=O)), 133.7 (4C, 4xS-(*Ar*)C-$\underline{C}$H-CH-C-O-(C=O)), 129.3, 129.4 (8C, 8xS-CH_2-(*Ar*)C-$\underline{C}$H-CH-C), 125.5, 125.7 (8C, 8xS-CH_2-(*Ar*)C-CH-$\underline{C}$H-C), 122.6 (4C, 4xS-(*Ar*)C-CH-$\underline{C}$H-C-O-(C=O)), 107.2 (4C, 4xCH_3-(*Ar*)C-CH-CH-$\underline{C}$), 100.7 (4C, 4xCH_3-(*Ar*)$\underline{C}$-CH-CH-C), 84.4 (4C, 4xCH_3-(*Ar*)C-CH-$\underline{C}$H-C), 83.8 (8C, 4xCH_3-(*Ar*)C-CH-$\underline{C}$H-C, 4xCH_3-(*Ar*)C-$\underline{C}$H-CH-C), 82.7 (4C, 4xCH_3-(*Ar*)C-$\underline{C}$H-CH-C), 40.1 (2C, 2xS-$\underline{C}H_2$-(*Ar*)C-CH-CH-C-C(CH_3)$_3$), 39.6 (2C, 2xS-$\underline{C}H_2$-(*Ar*)C-CH-CH-C-C(CH_3)$_3$), 34.93 (2C, 2xS-CH_2-(*Ar*)C-CH-CH-C-$\underline{C}$(CH_3)$_3$), 34.88 (2C, 2xS-CH_2-(*Ar*)C-CH-CH-C-$\underline{C}$(CH_3)$_3$), 34.4 (2C, 2xS-CH_2-(*Ar*)C-CH-CH-C-O-(C=O)-$\underline{C}H_2$), 31.56 (6C, 2xS-CH_2-(*Ar*)C-CH-CH-C-C($\underline{C}H_3$)$_3$), 31.55 (6C, 2xS-CH_2-(*Ar*)C-CH-CH-C-C($\underline{C}H_3$)$_3$), 31.0 (4C, 4x(*Ar*)CH-CH-C-$\underline{C}$H(CH_3)$_2$), 28.8 (1C, S-(*Ar*)C-CH-CH-C-O-(C=O)-(CH_2)$_2$-$\underline{C}H_2$), 24.8 (2C, 2xS-(*Ar*)C-CH-CH-C-O-(C=O)-CH_2-$\underline{C}H_2$), 23.2 (4C, 2x(*Ar*)CH-CH-C-CH($\underline{C}H_3$)$_2$), 22.7 (4C, 2x(*Ar*)CH-CH-C-CH($\underline{C}H_3$)$_2$), 18.3 (4C, 4x$\underline{C}H_3$-(*Ar*)C-CH-CH-C).

ESI-MS(+): *m/z* 1023.7460 $[M-2Cl]^{2+}$, calcd. for $C_{104}H_{136}O_4Ru_4S_6^{2+}$ 1023.4335, the isotopic pattern corresponds well to the calculated one.

R_f (CH_2Cl_2/CH_3OH 9:1 (v/v)) = 0.47.

Elemental analysis (%): calcd. for $C_{104}H_{136}Cl_2O_4Ru_4S_6\cdot 4CH_3OH$: C 57.76, H 6.82; found: C 57.96, H 6.96.

7. Compounds with two trithiolato-bridged ruthenium(II)-*p*-cymene units and alkyl diamide linkers

Synthesis of $[(\eta^6\text{-}p\text{-}MeC_6H_4Pr^i)_2Ru_2(\mu_2\text{-}SCH_2C_6H_4\text{-}p\text{-}Bu^t)_2(\mu_2\text{-}SC_6H_4\text{-}p\text{-}NH)\text{-}(CO)\text{-}(CO)\text{-}(p\text{-}NH\text{-}C_6H_4\text{-}\mu_2S)Ru_2(\mu_2\text{-}SCH_2C_6H_4\text{-}p\text{-}Bu^t)_2(\eta^6\text{-}p\text{-}MeC_6H_4Pr^i)_2]Cl_2$ (11)

To a solution of **4** (0.200 g, 0.202 mmol, 1 equiv.) in dry CH_2Cl_2 (20 mL) at 0°C under inert atmosphere (N_2) were added successively a solution of oxalyl dichloride (0.013 g, 0.104 mmol, 0.52 equiv.) in dry CH_2Cl_2 (3.3 mL) and TEA (0.1 mL, 0.727 mmol, 3.6 equiv.). The reaction mixture was then allowed to warm up to r.t. and further stirred overnight (20 h). Due

to incomplete reaction, an excess of oxalyl dichloride (0.006 g, 0.035 mmol, 0.18 equiv.) in dry CH_2Cl_2 (0.33 mL) was added and the reaction mixture was further stirred at r.t. for another 2 h. The reaction evolution was verified by ^{1}H-NMR and TLC and then the mixture was concentrated under reduced pressure. Purification by column chromatography using CH_2Cl_2/CH_3OH (9:1, v/v) allowed the isolation of **11** as an orange solid (0.090 g, 0.044 mmol, yield 44%).

^{1}H-NMR ($CDCl_3$) (δ_H, ppm): 10.57 (2H, s, 2xN$\underline{H}$-(C=O)), 8.05 (4H, d, 4xS-(*Ar*)C-CH-C$\underline{H}$-C-NH, $^3J_{H,H}$ = 8.6 Hz), 7.77 (4H, d, 4xS-(*Ar*)C-C$\underline{H}$-CH-C-NH, $^3J_{H,H}$ = 8.6 Hz), 7.41-7.51 (16H, m, 8xS-CH$_2$-(*Ar*)C-C$\underline{H}$-CH-C-C(CH$_3$)$_3$, 8xS-CH$_2$-(*Ar*)C-CH-C$\underline{H}$-C-C(CH$_3$)$_3$), 5.07 (4H, d, 4xCH$_3$-(*Ar*)C-CH-C$\underline{H}$-C, $^3J_{H,H}$ = 5.7 Hz), 4.97 (4H, d, 4xCH$_3$-(*Ar*)C-CH-C$\underline{H}$-C, $^3J_{H,H}$ = 5.8 Hz), 4.86 (4H, d, 4xCH$_3$-(*Ar*)C-C$\underline{H}$-CH-C, $^3J_{H,H}$ = 5.7 Hz), 4.64 (4H, d, 4xCH$_3$-(*Ar*)C-C$\underline{H}$-CH-C, $^3J_{H,H}$ = 5.8 Hz), 3.61 (4H, s, 2xS-C$\underline{H_2}$-(*Ar*)C-CH-CH-C-C(CH$_3$)$_3$), 3.41 (4H, s, 2xS-C$\underline{H_2}$-(*Ar*)C-CH-CH-C-C(CH$_3$)$_3$), 1.97 (4H, sept, 4x(*Ar*)C-CH-CH-C-C$\underline{H}$(CH$_3$)$_2$, $^3J_{H,H}$ = 6.9 Hz), 1.73 (12H, s, 4xC$\underline{H_3}$-(*Ar*)C-CH-CH-C), 1.38 (18H, s, 2xS-CH$_2$-(*Ar*)C-CH-CH-C-C(C$\underline{H_3}$)$_3$), 1.35 (18H, s, 2xS-CH$_2$-(*Ar*)C-CH-CH-C-C(C$\underline{H_3}$)$_3$), 0.97 (12H, d, 2x(*Ar*)C-CH-CH-C-CH(C$\underline{H_3}$)$_2$, $^3J_{H,H}$ = 6.9 Hz), 0.93 (12H, d, 2x(*Ar*)C-CH-CH-C-CH(C$\underline{H_3}$)$_2$, $^3J_{H,H}$ = 6.9 Hz).

^{13}C-NMR ($CDCl_3$) (δ_C, ppm): 158.0 (2C, 2xS-(*Ar*)C-CH-CH-C-NH-($\underline{C}$=O)), 151.96, 152.02 (4C, 4xS-CH$_2$-(*Ar*)C-CH-CH-$\underline{C}$-C(CH$_3$)$_3$), 138.3 (2C, 2xS-(*Ar*)$\underline{C}$-CH-CH-C-NH-(C=O)), 136.6, 136.8 (4C, 4xS-CH$_2$-(*Ar*)$\underline{C}$-CH-CH-C-C(CH$_3$)$_3$), 133.3 (6C, 4xS-(*Ar*)C-$\underline{C}$H-CH-C-NH-(C=O), 2xS-(*Ar*)C-CH-CH-$\underline{C}$-NH-(C=O)), 129.2, 129.4 (8C, 8xS-CH$_2$-(*Ar*)C-$\underline{C}$H-CH-C), 125.7, 125.8 (8C, 8xS-CH$_2$-(*Ar*)C-CH-$\underline{C}$H-C), 121.1 (4C, 4xS-(*Ar*)C-CH-$\underline{C}$H-C-NH-(C=O)), 107.6 (4C, 4xCH$_3$-(*Ar*)C-CH-CH-$\underline{C}$), 100.5 (4C, 4xCH$_3$-(*Ar*)$\underline{C}$-CH-CH-C), 84.1 (4C, 4xCH$_3$-(*Ar*)C-CH-$\underline{C}$H-C), 83.9 (4C, 4xCH$_3$-(*Ar*)C-$\underline{C}$H-CH-C), 83.7 (4C, 4xCH$_3$-(*Ar*)C-CH-$\underline{C}$H-C), 82.5 (4C, 4xCH$_3$-(*Ar*)C-$\underline{C}$H-CH-C), 40.0 (2C, 2xS-$\underline{C}$H$_2$-(*Ar*)C-CH-CH-C-C(CH$_3$)$_3$), 39.5 (2C, 2xS-$\underline{C}$H$_2$-(*Ar*)C-CH-CH-C-C(CH$_3$)$_3$), 34.96 (2C, 2xS-CH$_2$-(*Ar*)C-CH-CH-C-$\underline{C}$(CH$_3$)$_3$), 34.92 (2C, 2xS-CH$_2$-(*Ar*)C-CH-CH-C-$\underline{C}$(CH$_3$)$_3$), 31.6 (12C, 4xS-CH$_2$-(*Ar*)C-CH-CH-C-C($\underline{C}$H$_3$)$_3$), 31.1 (4C, 4x(*Ar*)CH-CH-C-$\underline{C}$H(CH$_3$)$_2$), 23.2 (4C, 2x(*Ar*)CH-CH-C-CH($\underline{C}$H$_3$)$_2$), 22.9 (4C, 2x(*Ar*)CH-CH-C-CH($\underline{C}$H$_3$)$_2$), 18.3 (4C, 4x$\underline{C}$H$_3$-(*Ar*)C-CH-CH-C).

ESI-MS(+): *m/z* 980.7171 [M-2Cl]$^{2+}$, calcd. for $C_{98}H_{126}N_2O_2Ru_4S_6^{2+}$ 980.3685, the isotopic pattern corresponds well to the calculated one.

R$_f$ (CH_2Cl_2/CH_3OH 9:1 (v/v)) = 0.52.

Elemental analysis (%): calcd for $C_{98}H_{126}Cl_2N_2O_2Ru_4S_6 \cdot 0.7CH_3OH \cdot 2.5H_2O$: C 56.48, H 6.43, N 1.33; found: C 56.46, H 6.54, N 1.33.

Synthesis of [(η^6-p-MeC$_6$H$_4$Pr^i)$_2$Ru$_2$(μ_2-SCH$_2$C$_6$H$_4$-p-Bu^t)$_2$(μ_2-SC$_6$H$_4$-p-NH)-(CO)(CH$_2$)$_4$(CO)-(p-NH-C$_6$H$_4$-μ_2S)Ru$_2$(μ_2-SCH$_2$C$_6$H$_4$-p-Bu^t)$_2$(η^6-p-MeC$_6$H$_4$Pr^i)$_2$]Cl$_2$ (12)

To a solution of **4** (0.199 g, 0.202 mmol, 1 equiv.) in dry CH_2Cl_2 (20 mL) at 0°C under inert atmosphere (N_2) were added successively a solution of adipoyl dichloride (0.019 g, 0.104 mmol, 0.52 equiv.) in dry CH_2Cl_2 (3.3 mL) and TEA (0.1 mL, 0.727 mmol, 3.6 equiv.). The reaction mixture was then allowed to warm to r.t. and further stirred overnight. The reaction evolution was verified by ^{1}H-NMR and TLC and then the mixture was concentrated under reduced pressure. Purification by column chromatography using CH_2Cl_2/CH_3OH (9:1, v/v) allowed the isolation of **12** as an orange solid (0.077 g, 0.037 mmol, yield 37%).

^{1}H-NMR (CDCl$_3$) (δ_H, ppm): 10.87 (2H, s, 2xN*H*-(C=O)), 8.05 (4H, d, 4xS-(*Ar*)C-CH-C*H*-C-NH, $^3J_{H,H}$ = 8.6 Hz), 7.62 (4H, d, 4xS-(*Ar*)C-C*H*-CH-C-NH, $^3J_{H,H}$ = 8.6 Hz), 7.39-7.49 (16H, m, 8xS-CH$_2$-(*Ar*)C-C*H*-CH-C-C(CH$_3$)$_3$, 8xS-CH$_2$-(*Ar*)C-CH-C*H*-C-C(CH$_3$)$_3$), 5.00 (4H, d, 4xCH$_3$-(*Ar*)C-CH-C*H*-C, $^3J_{H,H}$ = 5.7 Hz), 4.93 (4H, d, 4xCH$_3$-(*Ar*)C-CH-C*H*-C, $^3J_{H,H}$ = 5.8 Hz), 4.75 (4H, d, 4xCH$_3$-(*Ar*)C-C*H*-CH-C, $^3J_{H,H}$ = 5.7 Hz), 4.63 (4H, d, 4xCH$_3$-(*Ar*)C-C*H*-CH-C, $^3J_{H,H}$ = 5.8 Hz), 3.57 (4H, s, 2xS-C*H$_2$*-(*Ar*)C-CH-CH-C-C(CH$_3$)$_3$), 3.38 (4H, s, 2xS-C*H$_2$*-(*Ar*)C-CH-CH-C-C(CH$_3$)$_3$), 2.74-2.84 (4H, m, 2xS-(*Ar*)C-CH-CH-C-NH-(C=O)-C*H$_2$*), 1.98 (4H, sept, 4x(*Ar*)C-CH-CH-C-C*H*(CH$_3$)$_2$, $^3J_{H,H}$ = 6.9 Hz), 1.85-1.95 (4H, m, 2xS-(*Ar*)C-CH-CH-C-NH-(C=O)-CH$_2$-C*H$_2$*), 1.71 (12H, s, 4xC*H$_3$*-(*Ar*)C-CH-CH-C), 1.36 (18H, s, 2xS-CH$_2$-(*Ar*)C-CH-CH-C-C(C*H$_3$*)$_3$), 1.34 (18H, s, 2xS-CH$_2$-(*Ar*)C-CH-CH-C-C(C*H$_3$*)$_3$), 0.98 (12H, d, 2x(*Ar*)C-CH-CH-C-CH(C*H$_3$*)$_2$, $^3J_{H,H}$ = 6.9 Hz), 0.93 (12H, d, 2x(*Ar*)C-CH-CH-C-CH(C*H$_3$*)$_2$, $^3J_{H,H}$ = 6.9 Hz).

^{13}C-NMR (CDCl$_3$) (δ_C, ppm): 173.6 (2C, 2xS-(*Ar*)C-CH-CH-C-NH-(*C*=O)), 151.9, 152.0 (4C, 4xS-CH$_2$-(*Ar*)C-CH-CH-*C*-C(CH$_3$)$_3$), 141.0 (2C, 2xS-(*Ar*)*C*-CH-CH-C-NH-(C=O)), 136.6, 136.8 (4C, 4xS-CH$_2$-(*Ar*)*C*-CH-CH-C-C(CH$_3$)$_3$), 132.9 (4C, 4xS-(*Ar*)C-*C*H-CH-C-NH-(C=O)), 130.4 (2C, 2xS-(*Ar*)C-CH-CH-*C*-NH-(C=O)), 129.1, 129.4 (8C, 8xS-CH$_2$-(*Ar*)C-*C*H-CH-C), 125.6, 125.7 (8C, 8xS-CH$_2$-(*Ar*)C-CH-*C*H-C), 120.1 (4C, 4xS-(*Ar*)C-CH-*C*H-C-NH-(C=O)), 107.6 (4C, 4xCH$_3$-(*Ar*)C-CH-CH-*C*), 100.3 (4C, 4xCH$_3$-(*Ar*)*C*-CH-CH-C), 84.1 (4C, 4xCH$_3$-(*Ar*)C-CH-*C*H-C), 83.8 (4C, 4xCH$_3$-(*Ar*)C-*C*H-CH-C), 83.4 (4C, 4xCH$_3$-(*Ar*)C-CH-*C*H-C), 82.3 (4C, 4xCH$_3$-(*Ar*)C-*C*H-CH-C), 39.9 (2C, 2xS-*C*H$_2$-(*Ar*)C-CH-CH-C-C(CH$_3$)$_3$), 39.4 (2C, 2xS-*C*H$_2$-(*Ar*)C-CH-CH-C-C(CH$_3$)$_3$), 35.8 (2C, 2xS-CH$_2$-(*Ar*)C-CH-CH-C-NH-(C=O)-*C*H$_2$), 34.92 (2C, 2xS-CH$_2$-(*Ar*)C-CH-CH-C-*C*(CH$_3$)$_3$), 34.89 (2C, 2xS-CH$_2$-(*Ar*)C-CH-CH-C-*C*(CH$_3$)$_3$), 31.6 (12C, 4xS-CH$_2$-(*Ar*)C-CH-CH-C-C(*C*H$_3$)$_3$), 31.1 (4C, 4x(*Ar*)CH-CH-C-*C*H(CH$_3$)$_2$), 24.3 (2C, 2xS-(*Ar*)C-CH-CH-C-NH-(C=O)-CH$_2$-*C*H$_2$), 23.2 (4C, 2x(*Ar*)CH-CH-C-CH(*C*H$_3$)$_2$), 22.9 (4C, 2x(*Ar*)CH-CH-C-CH(*C*H$_3$)$_2$), 18.2 (4C, 4x*C*H$_3$-(*Ar*)C-CH-CH-C).

ESI-MS(+): *m/z* 1008.7530 [M-2Cl]$^{2+}$, calcd. for $C_{102}H_{134}N_2O_2Ru_4S_6^{2+}$ 1008.4230, the isotopic pattern corresponds well to the calculated one.

R$_f$ (CH_2Cl_2/CH_3OH 9:1 (v/v)) = 0.57.

Elemental analysis (%): calcd. for $C_{102}H_{134}Cl_2N_2O_2Ru_4S_6 \cdot 0.7CH_2Cl_2 \cdot 1.5H_2O$: C 56.73, H 6.42, N 1.29; found: C 56.72, H 6.46, N 1.37.

Synthesis of $[(\eta^6\text{-}p\text{-}MeC_6H_4Pr^i)_2Ru_2(\mu_2\text{-}SCH_2C_6H_4\text{-}p\text{-}Bu^t)_2(\mu_2\text{-}SC_6H_4\text{-}p\text{-}NH)\text{-}(CO)(CH_2)_5(CO)\text{-}(p\text{-}NH\text{-}C_6H_4\text{-}\mu_2S)Ru_2(\mu_2\text{-}SCH_2C_6H_4\text{-}p\text{-}Bu^t)_2(\eta^6\text{-}p\text{-}MeC_6H_4Pr^i)_2]Cl_2$ (13)

To a solution of heptanedioic acid (0.018 g, 0.114 mmol, 0.55 equiv.) in dry CH_2Cl_2 (20 mL) were added at r.t. under inert atmosphere (N_2), HOBt·H_2O (0.048 g, 0.312 mmol, 1.52 equiv.) and DIPEA (0.05 mL, 0.253 mmol, 1.25 equiv.) and the mixture was stirred for 10 min. Then were successively added EDCI (0.073 g, 0.378 mmol, 1.84 equiv.), **4** (0.203 g, 0.205 mmol, 1 equiv.) and DIPEA (0.05 mL, 0.253 mmol, 1.25 equiv.) and the reaction mixture was further stirred overnight (20 h). The reaction evolution was verified by ^{1}H-NMR and TLC and then the mixture was concentrated under reduced pressure. Purification by column chromatography using CH_2Cl_2/CH_3OH (9:1, v/v) allowed the isolation of **13** as an orange solid (0.091 g, 0.043 mmol, yield 43%).

^{1}H-NMR ($CDCl_3$) (δ_H, ppm): 10.95 (2H, s, 2xN$\underline{H}$-(C=O)), 8.11 (4H, d, 4xS-(*Ar*)C-CH-C$\underline{H}$-C-NH, $^3J_{H,H}$ = 8.5 Hz), 7.62 (4H, d, 4xS-(*Ar*)C-C$\underline{H}$-CH-C-NH, $^3J_{H,H}$ = 8.5 Hz), 7.40-7.49 (16H, m, 8xS-CH_2-(*Ar*)C-C$\underline{H}$-CH-C-C$(CH_3)_3$, 8xS-CH_2-(*Ar*)C-CH-C$\underline{H}$-C-C$(CH_3)_3$), 5.00 (4H, d, 4xCH_3-(*Ar*)C-CH-C$\underline{H}$-C, $^3J_{H,H}$ = 5.6 Hz), 4.93 (4H, d, 4xCH_3-(*Ar*)C-CH-C$\underline{H}$-C, $^3J_{H,H}$ = 5.6 Hz), 4.74 (4H, d, 4xCH_3-(*Ar*)C-C$\underline{H}$-CH-C, $^3J_{H,H}$ = 5.6 Hz), 4.64 (4H, d, 4xCH_3-(*Ar*)C-C$\underline{H}$-CH-C, $^3J_{H,H}$ = 5.6 Hz), 3.58 (4H, s, 2xS-C$\underline{H_2}$-(*Ar*)C-CH-CH-C-C$(CH_3)_3$), 3.39 (4H, s, 2xS-C$\underline{H_2}$-(*Ar*)C-CH-CH-C-C$(CH_3)_3$), 2.61-2.70 (4H, m, 2xS-(*Ar*)C-CH-CH-C-NH-(C=O)-C$\underline{H_2}$), 1.99 (4H, sept, 4x(*Ar*)C-CH-CH-C-C$\underline{H}$$(CH_3)_2$, $^3J_{H,H}$ = 6.8 Hz), 1.90-2.09 (4H, m, 2xS-(*Ar*)C-CH-CH-C-NH-(C=O)-CH_2-C$\underline{H_2}$), 1.71 (12H, s, 4xC$\underline{H_3}$-(*Ar*)C-CH-CH-C), 1.55-1.65 (2H, m, 2xS-(*Ar*)C-CH-CH-C-NH-(C=O)-$(CH_2)_2$-C$\underline{H_2}$), 1.37 (18H, s, 2xS-CH_2-(*Ar*)C-CH-CH-C-C(C$\underline{H_3}$)$_3$), 1.35 (18H, s, 2xS-CH_2-(*Ar*)C-CH-CH-C-C(C$\underline{H_3}$)$_3$), 0.99 (12H, d, 2x(*Ar*)C-CH-CH-C-CH(C$\underline{H_3}$)$_2$, $^3J_{H,H}$ = 6.8 Hz), 0.94 (12H, d, 2x(*Ar*)C-CH-CH-C-CH(C$\underline{H_3}$)$_2$, $^3J_{H,H}$ = 6.8 Hz).

^{13}C-NMR ($CDCl_3$) (δ_C, ppm): 174.4 (2C, 2xS-(*Ar*)C-CH-CH-C-NH-($\underline{C}$=O)), 151.9, 152.0 (4C, 4xS-CH_2-(*Ar*)C-CH-CH-$\underline{C}$-C$(CH_3)_3$), 141.3 (2C, 2xS-(*Ar*)$\underline{C}$-CH-CH-C-NH-(C=O)), 136.6, 136.8 (4C, 4xS-CH_2-(*Ar*)$\underline{C}$-CH-CH-C-C$(CH_3)_3$), 132.8 (4C, 4xS-(*Ar*)C-$\underline{C}$H-CH-C-NH-(C=O)), 130.1 (2C, 2xS-(*Ar*)C-CH-CH-$\underline{C}$-NH-(C=O)), 129.1, 129.4 (8C, 8xS-CH_2-(*Ar*)C-$\underline{C}$H-CH-C), 125.6, 125.7 (8C, 8xS-CH_2-(*Ar*)C-CH-$\underline{C}$H-C), 120.2 (4C, 4xS-(*Ar*)C-CH-$\underline{C}$H-C-NH-(C=O)), 107.8 (4C, 4xCH_3-(*Ar*)C-CH-CH-$\underline{C}$), 100.2 (4C, 4xCH_3-(*Ar*)$\underline{C}$-CH-CH-C), 84.3 (4C, 4xCH_3-(*Ar*)C-CH-$\underline{C}$H-C), 83.9 (4C, 4xCH_3-(*Ar*)C-$\underline{C}$H-CH-C), 83.3 (4C, 4xCH_3-(*Ar*)C-CH-$\underline{C}$H-C), 82.3 (4C, 4xCH_3-(*Ar*)C-$\underline{C}$H-CH-C), 39.8 (2C, 2xS-$\underline{C}H_2$-(*Ar*)C-CH-CH-C-C$(CH_3)_3$), 39.4 (2C, 2xS-$\underline{C}H_2$-(*Ar*)C-CH-CH-C-C$(CH_3)_3$), 37.2 (2C, 2xS-CH_2-

(*Ar*)C-CH-CH-C-NH-(C=O)-$\underline{C}H_2$), 34.94 (2C, 2xS-CH_2-(*Ar*)C-CH-CH-C-$\underline{C}(CH_3)_3$), 34.90 (2C, 2xS-CH_2-(*Ar*)C-CH-CH-C-$\underline{C}(CH_3)_3$), 31.6 (12C, 4xS-CH_2-(*Ar*)C-CH-CH-C-C($\underline{C}H_3)_3$), 31.1 (4C, 4x(*Ar*)CH-CH-C-$\underline{C}H(CH_3)_2$), 27.7 (1C, S-(*Ar*)C-CH-CH-C-NH-(C=O)-$(CH_2)_2$-$\underline{C}H_2$), 24.6 (2C, 2xS-(*Ar*)C-CH-CH-C-NH-(C=O)-CH_2-$\underline{C}H_2$), 23.2 (4C, 2x(*Ar*)CH-CH-C-CH($\underline{C}H_3)_2$), 23.0 (4C, 2x(*Ar*)CH-CH-C-CH($\underline{C}H_3)_2$), 18.2 (4C, 4x$\underline{C}H_3$-(*Ar*)C-CH-CH-C).

ESI-MS(+): *m/z* 1015.7526 $[M\text{-}2Cl]^{2+}$, calcd. for $C_{103}H_{136}N_2O_2Ru_4S_6^{2+}$ 1015.4360, the isotopic pattern corresponds well to the calculated one.

R_f (CH_2Cl_2/CH_3OH 9:1 (v/v)) = 0.57.

Elemental analysis (%): calcd. for $C_{103}H_{136}Cl_2N_2O_2Ru_4S_6$ ·2.5CH_2Cl_2·EDCI: C 55.38, H 6.49, N 2.82; found: C 55.19, H 6.78, N 2.87.

Synthesis of $[(\eta^6\text{-}p\text{-}MeC_6H_4Pr^i)_2Ru_2(\mu_2\text{-}SCH_2C_6H_4\text{-}p\text{-}Bu^t)_2(\mu_2\text{-}SC_6H_4\text{-}p\text{-}NH)\text{-}(CO)(CH_2)_6(CO)\text{-}(p\text{-}NH\text{-}C_6H_4\text{-}\mu_2S)Ru_2(\mu_2\text{-}SCH_2C_6H_4\text{-}p\text{-}Bu^t)_2(\eta^6\text{-}p\text{-}MeC_6H_4Pr^i)_2]Cl_2$ (14)

To a solution of **4** (0.200 g, 0.202 mmol, 1 equiv.) in dry CH_2Cl_2 (20 mL) at 0°C under inert atmosphere (N_2) were added successively a solution of octanedioyl dichloride (0.022 g, 0.103 mmol, 0.51 equiv.) in dry CH_2Cl_2 (2.4 mL) and TEA (0.1 mL, 0.727 mmol, 3.6 equiv.). The reaction mixture was then allowed to warm up to r.t. and further stirred overnight. The reaction evolution was verified by ^1H-NMR and TLC and then the mixture was concentrated under reduced pressure. Purification by column chromatography using CH_2Cl_2/CH_3OH (9:1, v/v) allowed the isolation of **14** as an orange solid (0.073 g, 0.035 mmol, yield 34%).

^1H-NMR ($CDCl_3$) (δ_H, ppm): 10.68 (2H, s, 2xN$\underline{H}$-(C=O)), 8.06 (4H, d, 4xS-(*Ar*)C-CH-C$\underline{H}$-C-NH, $^3J_{H,H}$ = 8.7 Hz), 7.60 (4H, d, 4xS-(*Ar*)C-C$\underline{H}$-CH-C-NH, $^3J_{H,H}$ = 8.7 Hz), 7.37-7.47 (16H, m, 8xS-CH_2-(*Ar*)C-C$\underline{H}$-CH-C-C$(CH_3)_3$, 8xS-CH_2-(*Ar*)C-CH-C$\underline{H}$-C-C$(CH_3)_3$), 4.98 (4H, d, 4xCH_3-(*Ar*)C-CH-C$\underline{H}$-C, $^3J_{H,H}$ = 5.8 Hz), 4.91 (4H, d, 4xCH_3-(*Ar*)C-CH-C$\underline{H}$-C, $^3J_{H,H}$ = 5.8 Hz), 4.72 (4H, d, 4xCH_3-(*Ar*)C-C$\underline{H}$-CH-C, $^3J_{H,H}$ = 5.8 Hz), 4.61 (4H, d, 4xCH_3-(*Ar*)C-C$\underline{H}$-CH-C, $^3J_{H,H}$ = 5.8 Hz), 3.55 (4H, s, 2xS-C$\underline{H_2}$-(*Ar*)C-CH-CH-C-C$(CH_3)_3$), 3.36 (4H, s, 2xS-C$\underline{H_2}$-(*Ar*)C-CH-CH-C-C$(CH_3)_3$), 2.56-2.66 (4H, m, 2xS-(*Ar*)C-CH-CH-C-NH-(C=O)-C$\underline{H_2}$), 1.96 (4H, sept, 4x(*Ar*)C-CH-CH-C-C$\underline{H}(CH_3)_2$, $^3J_{H,H}$ = 6.9 Hz), 1.80-1.90 (4H, m, 2xS-(*Ar*)C-CH-CH-C-NH-(C=O)-CH_2-C$\underline{H_2}$), 1.69 (12H, s, 4xC$\underline{H_3}$-(*Ar*)C-CH-CH-C), 1.46-1.55 (4H, m, 2xS-(*Ar*)C-CH-CH-C-NH-(C=O)-$(CH_2)_2$-C$\underline{H_2}$), 1.34 (18H, s, 2xS-CH_2-(*Ar*)C-CH-CH-C-C(C$\underline{H_3})_3$), 1.31 (18H, s, 2xS-CH_2-(*Ar*)C-CH-CH-C-C(C$\underline{H_3})_3$), 0.95 (12H, d, 2x(*Ar*)C-CH-CH-C-CH(C$\underline{H_3})_2$, $^3J_{H,H}$ = 6.9 Hz), 0.91 (12H, d, 2x(*Ar*)C-CH-CH-C-CH(C$\underline{H_3})_2$, $^3J_{H,H}$ = 6.9 Hz).

^{13}C-NMR ($CDCl_3$) (δ_C, ppm): 174.0 (2C, 2xS-(*Ar*)C-CH-CH-C-NH-($\underline{C}$=O)), 151.7, 151.9 (4C, 4xS-CH_2-(*Ar*)C-CH-CH-$\underline{C}$-C$(CH_3)_3$), 141.2 (2C, 2xS-(*Ar*)$\underline{C}$-CH-CH-C-NH-(C=O)), 136.6, 136.8 (4C, 4xS-CH_2-(*Ar*)$\underline{C}$-CH-CH-C-C$(CH_3)_3$), 132.8 (4C, 4xS-(*Ar*)C-$\underline{C}$H-CH-C-NH-(C=O)), 129.9 (2C, 2xS-(*Ar*)C-CH-CH-$\underline{C}$-NH-(C=O)), 129.0, 129.3 (8C, 8xS-CH_2-

(*Ar*)C-$\underline{C}$H-CH-C), 125.5, 125.6 (8C, 8xS-CH$_2$-(*Ar*)C-CH-$\underline{C}$H-C), 120.1 (4C, 4xS-(*Ar*)C-CH-$\underline{C}$H-C-NH-(C=O)), 107.6 (4C, 4xCH$_3$-(*Ar*)C-CH-CH-$\underline{C}$), 100.1 (4C, 4xCH$_3$-(*Ar*)$\underline{C}$-CH-CH-C), 84.2 (4C, 4xCH$_3$-(*Ar*)C-CH-$\underline{C}$H-C), 83.8 (4C, 4xCH$_3$-(*Ar*)C-$\underline{C}$H-CH-C), 83.3 (4C, 4xCH$_3$-(*Ar*)C-CH-$\underline{C}$H-C), 82.2 (4C, 4xCH$_3$-(*Ar*)C-$\underline{C}$H-CH-C), 39.8 (2C, 2xS-$\underline{C}$H$_2$-(*Ar*)C-CH-CH-C-C(CH$_3$)$_3$), 39.3 (2C, 2xS-$\underline{C}$H$_2$-(*Ar*)C-CH-CH-C-C(CH$_3$)$_3$), 38.0 (2C, 2xS-CH$_2$-(*Ar*)C-CH-CH-C-NH-(C=O)-$\underline{C}$H$_2$), 34.86 (2C, 2xS-CH$_2$-(*Ar*)C-CH-CH-C-$\underline{C}$(CH$_3$)$_3$), 34.82 (2C, 2xS-CH$_2$-(*Ar*)C-CH-CH-C-$\underline{C}$(CH$_3$)$_3$), 31.5 (12C, 4xS-CH$_2$-(*Ar*)C-CH-CH-C-C($\underline{C}$H$_3$)$_3$), 31.0 (4C, 4x(*Ar*)CH-CH-C-$\underline{C}$H(CH$_3$)$_2$), 27.6 (2C, 2xS-(*Ar*)C-CH-CH-C-NH-(C=O)-(CH$_2$)$_2$-$\underline{C}$H$_2$), 25.0 (2C, 2xS-(*Ar*)C-CH-CH-C-NH-(C=O)-CH$_2$-$\underline{C}$H$_2$), 23.1 (4C, 2x(*Ar*)CH-CH-C-CH($\underline{C}$H$_3$)$_2$), 23.0 (4C, 2x(*Ar*)CH-CH-C-CH($\underline{C}$H$_3$)$_2$), 18.2 (4C, 4x$\underline{C}$H$_3$-(*Ar*)C-CH-CH-C).
ESI-MS(+): *m/z* 1022.7640 [M-2Cl]$^{2+}$, calcd. for $C_{104}H_{138}N_2O_2Ru_4S_6^{2+}$ 1022.4495, the isotopic pattern corresponds well to the calculated one.
R$_f$ (CH$_2$Cl$_2$/CH$_3$OH 9:1 (v/v)) = 0.57.
Elemental analysis (%): calcd. for $C_{104}H_{138}Cl_2N_2O_2Ru_4S_6\cdot0.3CH_3OH\cdot2H_2O$: C 57.54, H 6.60, N 1.29; found: C 57.59, H 6.56, N 1.35.

8. Compounds with two trithiolato-bridged ruthenium(II)-*p*-cymene units and *meta* and *para* substituted diester and diamide linkers

Synthesis of [(η^6-p-MeC$_6$H$_4$Pr^i)$_2$Ru$_2$(μ_2-SCH$_2$C$_6$H$_4$-p-Bu^t)$_2$(μ_2-SC$_6$H$_4$-p-O)-(CO)-m-C$_6$H$_4$-(CO)-(p-O-C$_6$H$_4$-μ_2S)Ru$_2$(μ_2-SCH$_2$C$_6$H$_4$-p-Bu^t)$_2$(η^6-p-MeC$_6$H$_4$Pr^i)$_2$]Cl$_2$ (15)

To a solution of **3** (0.201 g, 0.203 mmol, 1equiv.) in 20 mL dry CH$_2$Cl$_2$ at 0°C under inert atmosphere (N$_2$) were added successively a solution of isophthaloyl dichloride (0.021 g, 0.103 mmol, 0.51 equiv.) in dry CH$_2$Cl$_2$ (5 mL) and TEA (0.1 mL, 0.727 mmol, 3.6 equiv.). The reaction mixture was then allowed to warm up to r.t. and further stirred overnight. The reaction evolution was verified by ^{1}H-NMR and TLC and then the mixture was concentrated under reduced pressure. Purification by column chromatography using CH$_2$Cl$_2$/CH$_3$OH (9:1, v/v) allowed the isolation of **15** as an orange solid (0.174 g, 0.083 mmol, yield 82%).
^{1}H-NMR (CDCl$_3$) (δ_H, ppm): 8.36 (1H, t, O-(C=O)-(*Ar*)C-C$\underline{H}$-C(*Ar*), $^4J_{H,H}$ = 1.7 Hz), 8.47 (2H, dd, 2xO-(C=O)-(*Ar*)C-C$\underline{H}$-CH, $^3J_{H,H}$ = 7.8 Hz, $^4J_{H,H}$ = 1.7 Hz), 7.89 (4H, d, 4xS-(*Ar*)C-C$\underline{H}$-CH-C-O, $^3J_{H,H}$ = 8.7 Hz), 7.73 (1H, t, O-(C=O)-(*Ar*)C-CH-C$\underline{H}$, $^3J_{H,H}$ = 7.8 Hz), 7.42-7.50 (16H, m, 8xS-CH$_2$-(*Ar*)C-C$\underline{H}$-CH-C-C(CH$_3$)$_3$, 8xS-CH$_2$-(*Ar*)C-CH-C$\underline{H}$-C-C(CH$_3$)$_3$), 7.28 (4H, d, 4xS-(*Ar*)C-CH-C$\underline{H}$-C-O), $^3J_{H,H}$ = 8.7 Hz), 5.16 (4H, d, 4xCH$_3$-(*Ar*)C-CH-C$\underline{H}$-C, $^3J_{H,H}$ = 5.8 Hz), 5.02 (4H, d, 4xCH$_3$-(*Ar*)C-CH-C$\underline{H}$-C, $^3J_{H,H}$ = 5.9 Hz), 4.97 (4H, d, 4xCH$_3$-(*Ar*)C-C$\underline{H}$-CH-C, $^3J_{H,H}$ = 5.8 Hz), 4.64 (4H, d, 4xCH$_3$-(*Ar*)C-C$\underline{H}$-CH-C, $^3J_{H,H}$ = 5.9 Hz), 3.62 (4H, s, 2xS-C$\underline{H_2}$-(*Ar*)C-CH-CH-C-C(CH$_3$)$_3$), 3.44 (4H, s, 2xS-C$\underline{H_2}$-(*Ar*)C-CH-CH-C-C(CH$_3$)$_3$), 1.94 (4H, sept, 4x(*Ar*)C-CH-CH-C-C$\underline{H}$(CH$_3$)$_2$, $^3J_{H,H}$ = 6.9 Hz), 1.80 (12H, s, 4xC$\underline{H_3}$-(*Ar*)C-CH-CH-C), 1.37 (18H, s, 2xS-CH$_2$-(*Ar*)C-CH-CH-C-C(C$\underline{H_3}$)$_3$), 1.33 (18H, s,

2xS-CH_2-(*Ar*)C-CH-CH-C-C(C$\underline{H}_3$)$_3$), 0.98 (12H, d, 2x(*Ar*)C-CH-CH-C-CH(C$\underline{H}_3$)$_2$, $^3J_{H,H}$ = 6.9 Hz), 0.92 (12H, d, 2x(*Ar*)C-CH-CH-C-CH(C$\underline{H}_3$)$_2$, $^3J_{H,H}$ = 6.9 Hz).
^{13}C-NMR ($CDCl_3$) (δ_C, ppm): 164.1 (2C, 2xS-(*Ar*)C-CH-CH-C-O-($\underline{C}$=O)), 151.9, 152.0 (4C, 4xS-CH_2-(*Ar*)C-CH-CH-$\underline{C}$-C(CH_3)$_3$), 151.2 (2C, 2xS-(*Ar*)C-CH-CH-$\underline{C}$-O-(C=O)), 136.8 (4C, 4xS-CH_2-(*Ar*)$\underline{C}$-CH-CH-C-C(CH_3)$_3$), 135.5 (2C, 2xS-(*Ar*)$\underline{C}$-CH-CH-C-O-(C=O)), 135.4 (2C, 2xO-(C=O)-(*Ar*)C-$\underline{C}$H-CH), 133.9 (4C, 4xS-(*Ar*)C-$\underline{C}$H-CH-C-O-(C=O)), 131.9 (1C, O-(C=O)-(*Ar*)C-$\underline{C}$H-C(*Ar*)), 130.2 (2C, 2xO-(C=O)-(*Ar*)$\underline{C}$-CH), 129.5 (1C, O-(C=O)-(*Ar*)C-CH-$\underline{C}$H), 129.3, 129.4 (8C, 8xS-CH_2-(*Ar*)C-$\underline{C}$H-CH-C), 125.6, 125.8 (8C, 8xS-CH_2-(*Ar*)C-CH-$\underline{C}$H-C), 122.7 (4C, 4xS-(*Ar*)C-CH-$\underline{C}$H-C-O-(C=O)), 107.2 (4C, 4xCH_3-(*Ar*)C-CH-CH-$\underline{C}$), 100.8 (4C, 4xCH_3-(*Ar*)$\underline{C}$-CH-CH-C), 84.3 (4C, 4xCH_3-(*Ar*)C-CH-$\underline{C}$H-C), 83.9 (4C, 4xCH_3-(*Ar*)C-$\underline{C}$H-CH-C), 83.8 (4C, 4xCH_3-(*Ar*)C-CH-$\underline{C}$H-C), 82.7 (4C, 4xCH_3-(*Ar*)C-$\underline{C}$H-CH-C), 40.2 (2C, 2xS-$\underline{C}H_2$-(*Ar*)C-CH-CH-C-C(CH_3)$_3$), 39.7 (2C, 2xS-$\underline{C}H_2$-(*Ar*)C-CH-CH-C-C(CH_3)$_3$), 34.95 (2C, 2xS-CH_2-(*Ar*)C-CH-CH-C-$\underline{C}$(CH_3)$_3$), 34.90 (2C, 2xS-CH_2-(*Ar*)C-CH-CH-C-$\underline{C}$(CH_3)$_3$), 31.57 (6C, 2xS-CH_2-(*Ar*)C-CH-CH-C-C($\underline{C}H_3$)$_3$), 31.56 (6C, 2xS-CH_2-(*Ar*)C-CH-CH-C-C($\underline{C}H_3$)$_3$), 31.0 (4C, 4x(*Ar*)CH-CH-C-$\underline{C}$H(CH_3)$_2$), 23.3 (4C, 2x(*Ar*)CH-CH-C-CH($\underline{C}H_3$)$_2$), 22.8 (4C, 2x(*Ar*)CH-CH-C-CH($\underline{C}H_3$)$_2$), 18.4 (4C, 4x$\underline{C}H_3$-(*Ar*)C-CH-CH-C).
ESI-MS(+): *m/z* 1019.7156 $[M\text{-}2Cl]^{2+}$, calcd. for $C_{104}H_{128}O_4Ru_4S_6^{2+}$ 1019.4015, the isotopic pattern corresponds well to the calculated one.
R_f (CH_2Cl_2/CH_3OH 9:1 (v/v)) = 0.38.
Elemental analysis (%): calcd. for $C_{104}H_{128}Cl_2O_4Ru_4S_6\cdot 2CH_3OH$: C 58.57, H 6.31; found: C 58.52, H 6.53.

Synthesis of $[(\eta^6\text{-}p\text{-}MeC_6H_4Pr^i)_2Ru_2(\mu_2\text{-}SCH_2C_6H_4\text{-}p\text{-}Bu^t)_2(\mu_2\text{-}SC_6H_4\text{-}p\text{-}O)\text{-}(CO)\text{-}p\text{-}C_6H_4\text{-}(CO)\text{-}(p\text{-}O\text{-}C_6H_4\text{-}\mu_2S)Ru_2(\mu_2\text{-}SCH_2C_6H_4\text{-}p\text{-}Bu^t)_2(\eta^6\text{-}p\text{-}MeC_6H_4Pr^i)_2]Cl_2$ (16)

To a solution of **3** (0.203 g, 0.205 mmol, 1 equiv.) in 20 mL dry CH_2Cl_2 at 0°C under inert atmosphere (N_2) were added successively a solution of terephthaloyl dichloride (0.025 g, 0.123 mmol, 0.6 equiv.) in dry CH_2Cl_2 (5 mL) and TEA (0.09 mL, 0.656 mmol, 3.2 equiv.). The reaction mixture was then allowed to warm up to r.t. and further stirred overnight. The reaction evolution was verified by ^{1}H-NMR and TLC and then the mixture was concentrated under reduced pressure. Purification by column chromatography using CH_2Cl_2/CH_3OH (10:1, v/v) allowed the isolation of **16** as an orange solid (0.064 g, 0.030 mmol, yield 32%).
^{1}H-NMR ($CDCl_3$) (δ_H, ppm): 8.36 (4H, s, 4xO-(C=O)-(*Ar*)C-C$\underline{H}$), 7.89 (4H, d, 4xS-(*Ar*)C-C$\underline{H}$-CH-C-O, $^3J_{H,H}$ = 8.6 Hz), 7.42-7.50 (16H, m, 8xS-CH_2-(*Ar*)C-C$\underline{H}$-CH-C-C(CH_3)$_3$, 8xS-CH_2-(*Ar*)C-CH-C$\underline{H}$-C-C(CH_3)$_3$), 7.26 (4H, d, 4xS-(*Ar*)C-CH-C$\underline{H}$-C-O, $^3J_{H,H}$ = 8.6 Hz), 5.17 (4H, d, 4xCH_3-(*Ar*)C-CH-C$\underline{H}$-C, $^3J_{H,H}$ = 5.8 Hz), 5.04 (4H, d, 4xCH_3-(*Ar*)C-CH-C$\underline{H}$-C, $^3J_{H,H}$ = 5.8 Hz), 4.97 (4H, d, 4xCH_3-(*Ar*)C-C$\underline{H}$-CH-C, $^3J_{H,H}$ = 5.8 Hz), 4.65 (4H, d, 4xCH_3-(*Ar*)C-

C$\underline{H}$-CH-C, $^3J_{H,H}$ = 5.8 Hz), 3.63 (4H, s, 2xS-C$\underline{H_2}$-(*Ar*)C-CH-CH-C-C(CH_3)$_3$), 3.45 (4H, s, 2xS-C$\underline{H_2}$-(*Ar*)C-CH-CH-C-C(CH_3)$_3$), 1.94 (4H, sept, 4x(*Ar*)C-CH-CH-C-C$\underline{H}$(CH_3)$_2$, $^3J_{H,H}$ = 6.9 Hz), 1.81 (12H, s, 4xC$\underline{H_3}$-(*Ar*)C-CH-CH-C), 1.37 (18H, s, 2xS-CH_2-(*Ar*)C-CH-CH-C-C(C$\underline{H_3}$)$_3$), 1.34 (18H, s, 2xS-CH_2-(*Ar*)C-CH-CH-C-C(C$\underline{H_3}$)$_3$), 0.98 (12H, d, 2x(*Ar*)C-CH-CH-C-CH(C$\underline{H_3}$)$_2$, $^3J_{H,H}$ = 6.9 Hz), 0.92 (12H, d, 2x(*Ar*)C-CH-CH-C-CH(C$\underline{H_3}$)$_2$, $^3J_{H,H}$ = 6.9 Hz).

^{13}C-NMR ($CDCl_3$) (δ_C, ppm): 164.1 (2C, 2xS-(*Ar*)C-CH-CH-C-O-($\underline{C}$=O)), 151.8, 151.9 (4C, 4xS-CH_2-(*Ar*)C-CH-CH-$\underline{C}$-C(CH_3)$_3$), 151.2 (2C, 2xS-(*Ar*)C-CH-CH-$\underline{C}$-O-(C=O)), 136.8 (4C, 4xS-CH_2-(*Ar*)$\underline{C}$-CH-CH-C-C(CH_3)$_3$), 135.6 (2C, 2xS-(*Ar*)$\underline{C}$-CH-CH-C-O-(C=O)), 133.9 (2C, 2xO-(C=O)-(*Ar*)$\underline{C}$-CH), 133.8 (4C, 4xS-(*Ar*)C-$\underline{C}$H-CH-C-O-(C=O)), 130.6 (4C, 4xO-(C=O)-(*Ar*)C-$\underline{C}$H), 129.2, 129.4 (8C, 8xS-CH_2-(*Ar*)C-$\underline{C}$H-CH-C), 125.6, 125.7 (8C, 8xS-CH_2-(*Ar*)C-CH-$\underline{C}$H-C), 122.5 (4C, 4xS-(*Ar*)C-CH-$\underline{C}$H-C-O-(C=O)), 107.2 (4C, 4xCH_3-(*Ar*)C-CH-CH-$\underline{C}$), 100.7 (4C, 4xCH_3-(*Ar*)$\underline{C}$-CH-CH-C), 84.3 (4C, 4xCH_3-(*Ar*)C-CH-$\underline{C}$H-C), 83.83 (4C, 4xCH_3-(*Ar*)C-$\underline{C}$H-CH-C), 83.78 (4C, 4xCH_3-(*Ar*)C-CH-$\underline{C}$H-C), 82.7 (4C, 4xCH_3-(*Ar*)C-$\underline{C}$H-CH-C), 40.2 (2C, 2xS-$\underline{C}H_2$-(*Ar*)C-CH-CH-C-C(CH_3)$_3$), 39.7 (2C, 2xS-$\underline{C}H_2$-(*Ar*)C-CH-CH-C-C(CH_3)$_3$), 34.92 (2C, 2xS-CH_2-(*Ar*)C-CH-CH-C-$\underline{C}$(CH_3)$_3$), 34.88 (2C, 2xS-CH_2-(*Ar*)C-CH-CH-C-$\underline{C}$(CH_3)$_3$), 31.55 (6C, 2xS-CH_2-(*Ar*)C-CH-CH-C-C($\underline{C}H_3$)$_3$), 31.53 (6C, 2xS-CH_2-(*Ar*)C-CH-CH-C-C($\underline{C}H_3$)$_3$), 31.0 (4C, 4x(*Ar*)CH-CH-C-$\underline{C}$H(CH_3)$_2$), 23.2 (4C, 2x(*Ar*)CH-CH-C-CH($\underline{C}H_3$)$_2$), 22.7 (4C, 2x(*Ar*)CH-CH-C-CH($\underline{C}H_3$)$_2$), 18.3 (4C, 4x$\underline{C}H_3$-(*Ar*)C-CH-CH-C).

ESI-MS(+): *m/z* 1019.7151 [M-2Cl]$^{2+}$, calcd. for $C_{104}H_{128}O_4Ru_4S_6^{2+}$ 1019.4015, the isotopic pattern corresponds well to the calculated one.

R$_f$ (CH_2Cl_2/CH_3OH 9:1 (v/v)) = 0.36.

Elemental analysis (%): calcd. for $C_{104}H_{128}Cl_2O_4Ru_4S_6 \cdot 2.3H_2O$: C 58.07, H 6.21; found: C 58.1, H 6.31.

Synthesis of [(η^6-p-$MeC_6H_4Pr^i$)$_2Ru_2$(μ_2-$SCH_2C_6H_4$-p-Bu^t)$_2$(μ_2-SC_6H_4-p-NH)-(CO)-m-C_6H_4-(CO)-(p-NH-C_6H_4-μ_2S)Ru_2(μ_2-$SCH_2C_6H_4$-p-Bu^t)$_2$(η^6-p-$MeC_6H_4Pr^i$)$_2$]Cl_2 (17)

To a solution of **4** (0.200 g, 0.202 mmol, 1 equiv.) in dry CH_2Cl_2 (20 mL) at 0°C under inert atmosphere (N_2) were added successively a solution of isophthaloyl dichloride (0.013 g, 0.104 mmol, 0.52 equiv.) in dry CH_2Cl_2 (3.3 mL) and TEA (0.1 mL, 0.727 mmol, 3.6 equiv.). The reaction mixture was then allowed to warm up to r.t. and further stirred overnight (20 h). The reaction evolution was verified by ^{1}H-NMR and TLC and the mixture was then concentrated under reduced pressure. Purification by column chromatography using CH_2Cl_2/CH_3OH (9:1, v/v) allowed the isolation of **17** as an orange solid (0.107 g, 0.051 mmol, yield 50%).

^{1}H-NMR (CDCl$_3$) (δ$_H$, ppm): 11.27 (2H, s, 2xN*H*-(C=O)), 9.37 (1H, m, NH-(C=O)-(*Ar*)C-C*H*-C(*Ar*)), 8.41 (4H, d, 4xS-(*Ar*)C-CH-C*H*-C-NH, $^3J_{H,H}$ = 8.8 Hz), 8.24 (2H, dd, 2xNH-(C=O)-(*Ar*)C-C*H*-CH, $^3J_{H,H}$ = 7.8 Hz, $^4J_{H,H}$ = 1.4 Hz), 7.70 (4H, d, 4xS-(*Ar*)C-C*H*-CH-C-NH), $^3J_{H,H}$ = 8.8 Hz), 7.60 (1H, t, NH-(C=O)-(*Ar*)C-CH-C*H*, $^3J_{H,H}$ = 7.8 Hz), 7.42-7.50 (16H, m, 8xS-CH$_2$-(*Ar*)C-C*H*-CH-C-C(CH$_3$)$_3$, 8xS-CH$_2$-(*Ar*)C-CH-C*H*-C-C(CH$_3$)$_3$), 5.02 (4H, d, 4xCH$_3$-(*Ar*)C-CH-C*H*-C, $^3J_{H,H}$ = 5.8 Hz), 4.95 (4H, d, 4xCH$_3$-(*Ar*)C-CH-C*H*-C, $^3J_{H,H}$ = 5.9 Hz), 4.77 (4H, d, 4xCH$_3$-(*Ar*)C-C*H*-CH-C, $^3J_{H,H}$ = 5.8 Hz), 4.66 (4H, d, 4xCH$_3$-(*Ar*)C-C*H*-CH-C, $^3J_{H,H}$ = 5.8 Hz), 3.60 (4H, s, 2xS-C*H$_2$*-(*Ar*)C-CH-CH-C-C(CH$_3$)$_3$), 3.40 (4H, s, 2xS-C*H$_2$*-(*Ar*)C-CH-CH-C-C(CH$_3$)$_3$), 2.01 (4H, sept, 4x(*Ar*)C-CH-CH-C-C*H*(CH$_3$)$_2$, $^3J_{H,H}$ = 6.9 Hz), 1.73 (12H, s, 4xC*H$_3$*-(*Ar*)C-CH-CH-C), 1.37 (18H, s, 2xS-CH$_2$-(*Ar*)C-CH-CH-C-C(C*H$_3$*)$_3$), 1.35 (18H, s, 2xS-CH$_2$-(*Ar*)C-CH-CH-C-C(C*H$_3$*)$_3$), 1.00 (12H, d, 2x(*Ar*)C-CH-CH-C-CH(C*H$_3$*)$_2$, $^3J_{H,H}$ = 6.9 Hz), 0.96 (12H, d, 2x(*Ar*)C-CH-CH-C-CH(C*H$_3$*)$_2$, $^3J_{H,H}$ = 6.9 Hz).

^{13}C-NMR (CDCl$_3$) (δ$_C$, ppm): 166.5 (2C, 2xS-(*Ar*)C-CH-CH-C-NH-(C=O)), 151.9, 152.0 (4C, 4xS-CH$_2$-(*Ar*)C-CH-CH-C-C(CH$_3$)$_3$), 141.3 (2C, 2xS-(*Ar*)C-CH-CH-C-NH-(C=O)), 136.6, 136.8 (4C, 4xS-CH$_2$-(*Ar*)C-CH-CH-C-C(CH$_3$)$_3$), 134.7 (2C, 2xNH-(C=O)-(*Ar*)C-CH), 132.9 (4C, 4xS-(*Ar*)C-CH-CH-C-NH-(C=O)), 132.3 (2C, 2xNH-(C=O)-(*Ar*)C-CH-CH), 130.9 (2C, 2xS-(*Ar*)C-CH-CH-C-NH-(C=O)), 129.1, 129.4 (8C, 8xS-CH$_2$-(*Ar*)C-CH-CH-C), 129.0 (1C, NH-(C=O)-(*Ar*)C-CH-CH), 126.9 (1C, NH-(C=O)-(*Ar*)C-CH-C(*Ar*)), 125.7, 125.8 (8C, 8xS-CH$_2$-(*Ar*)C-CH-CH-C), 121.1 (4C, 4xS-(*Ar*)C-CH-CH-C-NH-(C=O)), 107.8 (4C, 4xCH$_3$-(*Ar*)C-CH-CH-C), 100.3 (4C, 4xCH$_3$-(*Ar*)C-CH-CH-C), 84.2 (4C, 4xCH$_3$-(*Ar*)C-CH-CH-C), 83.9 (4C, 4xCH$_3$-(*Ar*)C-CH-CH-C), 83.3 (4C, 4xCH$_3$-(*Ar*)C-CH-CH-C), 82.3 (4C, 4xCH$_3$-(*Ar*)C-CH-CH-C), 40.0 (2C, 2xS-CH$_2$-(*Ar*)C-CH-CH-C-C(CH$_3$)$_3$), 39.4 (2C, 2xS-CH$_2$-(*Ar*)C-CH-CH-C-C(CH$_3$)$_3$), 34.96 (2C, 2xS-CH$_2$-(*Ar*)C-CH-CH-C-C(CH$_3$)$_3$), 34.92 (2C, 2xS-CH$_2$-(*Ar*)C-CH-CH-C-C(CH$_3$)$_3$), 31.58 (6C, 2xS-CH$_2$-(*Ar*)C-CH-CH-C-C(CH$_3$)$_3$), 31.56 (6C, 2xS-CH$_2$-(*Ar*)C-CH-CH-C-C(CH$_3$)$_3$), 31.1 (4C, 4x(*Ar*)CH-CH-C-CH(CH$_3$)$_2$), 23.2 (4C, 2x(*Ar*)CH-CH-C-CH(CH$_3$)$_2$), 23.0 (4C, 2x(*Ar*)CH-CH-C-CH(CH$_3$)$_2$), 18.3 (4C, 4xCH$_3$-(*Ar*)C-CH-CH-C).

ESI-MS(+): *m/z* 1018.7381 [M-2Cl]$^{2+}$, calcd. for $C_{104}H_{130}N_2O_2Ru_4S_6^{2+}$ 1018.4175, the isotopic pattern corresponds well to the calculated one.

R$_f$ (CH_2Cl_2/CH_3OH 9:1 (v/v)) = 0.50.

Elemental analysis (%): calcd for $C_{104}H_{130}Cl_2N_2O_2Ru_4S_6\cdot 2H_2O$: C 58.27, H 6.30, N 1.31; found: C 58.25, H 6.31, N 1.35.

Synthesis of [(η^6-p-MeC$_6$H$_4$Pr^i)$_2$Ru$_2$(μ_2-SCH$_2$C$_6$H$_4$-p-Bu^t)$_2$(μ_2-SC$_6$H$_4$-p-NH)-(CO)-p-C$_6$H$_4$-(CO)-(p-NH-C$_6$H$_4$-μ_2S)Ru$_2$(μ_2-SCH$_2$C$_6$H$_4$-p-Bu^t)$_2$(η^6-p-MeC$_6$H$_4$Pr^i)$_2$]Cl$_2$ (18)

To a solution of **4** (0.200 g, 0.202 mmol, 1 equiv.) in dry CH_2Cl_2 (20 mL) at 0°C under inert atmosphere (N_2) were added successively a solution of terephthaloyl dichloride (0.013 g, 0.104 mmol, 0.52 equiv.) in dry CH_2Cl_2 (3.3 mL) and TEA (0.1 mL, 0.727 mmol, 3.6 equiv.). The reaction mixture was then allowed to warm up to r.t. and further stirred overnight (20 h). The reaction evolution was verified by ^{1}H-NMR and TLC and then the mixture was concentrated under reduced pressure. Purification by column chromatography using CH_2Cl_2/CH_3OH (9:1, v/v) allowed the isolation of **18** as an orange solid (0.045 g, 0.021 mmol, yield 21%).

^{1}H-NMR (CDCl$_3$) (δ_H, ppm): 10.42 (2H, s, 2xN$\underline{H}$-(C=O)), 8.32 (4H, s, 4xNH-(C=O)-(*Ar*)C-C$\underline{H}$), 8.23 (4H, d, 4xS-(*Ar*)C-CH-C$\underline{H}$-C-NH, $^3J_{H,H}$ = 8.7 Hz), 7.68 (4H, d, 4xS-(*Ar*)C-C$\underline{H}$-CH-C-NH, $^3J_{H,H}$ = 8.7 Hz), 7.39-7.49 (16H, m, 8xS-CH$_2$-(*Ar*)C-C$\underline{H}$-CH-C-C(CH$_3$)$_3$, 8xS-CH$_2$-(*Ar*)C-CH-C$\underline{H}$-C-C(CH$_3$)$_3$), 5.03 (4H, d, 4xCH$_3$-(*Ar*)C-CH-C$\underline{H}$-C, $^3J_{H,H}$ = 5.8 Hz), 4.94 (4H, d, 4xCH$_3$-(*Ar*)C-CH-C$\underline{H}$-C, $^3J_{H,H}$ = 5.8 Hz), 4.79 (4H, d, 4xCH$_3$-(*Ar*)C-C$\underline{H}$-CH-C, $^3J_{H,H}$ = 5.8 Hz), 4.64 (4H, d, 4xCH$_3$-(*Ar*)C-C$\underline{H}$-CH-C, $^3J_{H,H}$ = 5.8 Hz), 3.58 (4H, s, 2xS-C$\underline{H_2}$-(*Ar*)C-CH-CH-C-C(CH$_3$)$_3$), 3.39 (4H, s, 2xS-C$\underline{H_2}$-(*Ar*)C-CH-CH-C-C(CH$_3$)$_3$), 1.99 (4H, sept, 4x(*Ar*)C-CH-CH-C-C$\underline{H}$(CH$_3$)$_2$, $^3J_{H,H}$ = 6.9 Hz), 1.72 (12H, s, 4xC$\underline{H_3}$-(*Ar*)C-CH-CH-C), 1.36 (18H, s, 2xS-CH$_2$-(*Ar*)C-CH-CH-C-C(C$\underline{H_3}$)$_3$), 1.33 (18H, s, 2xS-CH$_2$-(*Ar*)C-CH-CH-C-C(C$\underline{H_3}$)$_3$), 0.98 (12H, d, 2x(*Ar*)C-CH-CH-C-CH(C$\underline{H_3}$)$_2$, $^3J_{H,H}$ = 6.9 Hz), 0.94 (12H, d, 2x(*Ar*)C-CH-CH-C-CH(C$\underline{H_3}$)$_2$, $^3J_{H,H}$ = 6.9 Hz).

^{13}C-NMR (CDCl$_3$) (δ_C, ppm): 166.4 (2C, 2xS-(*Ar*)C-CH-CH-C-NH-($\underline{C}$=O)), 151.8, 151.9 (4C, 4xS-CH$_2$-(*Ar*)C-CH-CH-$\underline{C}$-C(CH$_3$)$_3$), 140.6 (2C, 2xS-(*Ar*)$\underline{C}$-CH-CH-C-NH-(C=O)), 137.4 (2C, 2xNH-(C=O)-(*Ar*)$\underline{C}$-CH), 136.6, 136.8 (4C, 4xS-CH$_2$-(*Ar*)$\underline{C}$-CH-CH-C-C(CH$_3$)$_3$), 132.9 (4C, 4xS-(*Ar*)C-$\underline{C}$H-CH-C-NH-(C=O)), 131.4 (2C, 2xS-(*Ar*)C-CH-CH-$\underline{C}$-NH-(C=O)), 129.1, 129.4 (8C, 8xS-CH$_2$-(*Ar*)C-$\underline{C}$H-CH-C), 128.5 (4C, 4xNH-(C=O)-(*Ar*)C-$\underline{C}$H), 125.6, 125.7 (8C, 8xS-CH$_2$-(*Ar*)C-CH-$\underline{C}$H-C), 121.2 (4C, 4xS-(*Ar*)C-CH-$\underline{C}$H-C-NH-(C=O)), 107.6 (4C, 4xCH$_3$-(*Ar*)C-CH-CH-$\underline{C}$), 100.4 (4C, 4xCH$_3$-(*Ar*)$\underline{C}$-CH-CH-C), 84.1 (4C, 4xCH$_3$-(*Ar*)C-CH-$\underline{C}$H-C), 83.8 (4C, 4xCH$_3$-(*Ar*)C-$\underline{C}$H-CH-C), 83.5 (4C, 4xCH$_3$-(*Ar*)C-CH-$\underline{C}$H-C), 82.3 (4C, 4xCH$_3$-(*Ar*)C-$\underline{C}$H-CH-C), 40.0 (2C, 2xS-$\underline{C}$H$_2$-(*Ar*)C-CH-CH-C-C(CH$_3$)$_3$), 39.4 (2C, 2xS-$\underline{C}$H$_2$-(*Ar*)C-CH-CH-C-C(CH$_3$)$_3$), 34.91 (2C, 2xS-CH$_2$-(*Ar*)C-CH-CH-C-$\underline{C}$(CH$_3$)$_3$), 34.88 (2C, 2xS-CH$_2$-(*Ar*)C-CH-CH-C-$\underline{C}$(CH$_3$)$_3$), 31.55 (6C, 2xS-CH$_2$-(*Ar*)C-CH-CH-C-C($\underline{C}$H$_3$)$_3$), 31.54 (6C, 2xS-CH$_2$-(*Ar*)C-CH-CH-C-C($\underline{C}$H$_3$)$_3$), 31.1 (4C, 4x(*Ar*)CH-CH-C-$\underline{C}$H(CH$_3$)$_2$), 23.2 (4C, 2x(*Ar*)CH-CH-C-CH($\underline{C}$H$_3$)$_2$), 22.9 (4C, 2x(*Ar*)CH-CH-C-CH($\underline{C}$H$_3$)$_2$), 18.2 (4C, 4x$\underline{C}$H$_3$-(*Ar*)C-CH-CH-C).

ESI-MS(+): *m/z* 1018.7375 $[M-2Cl]^{2+}$, calcd. for $C_{104}H_{130}N_2O_2Ru_4S_6^{2+}$ 1018.4175, the isotopic pattern corresponds well to the calculated one.

R$_f$ (CH_2Cl_2/CH_3OH 9:1 (v/v)) = 0.50.

Elemental analysis (%): calcd for $C_{104}H_{130}Cl_2N_2O_2Ru_4S_6 \cdot CH_2Cl_2 \cdot 3H_2O$: C 56.13, H 6.19, N 1.25; found: C 56.08, H 6.18, N 1.33.

9. Compound with three trithiolato-bridged ruthenium(II)-*p*-cymene units with linear distribution

Synthesis of $[(\eta^6\text{-}p\text{-}MeC_6H_4Pr^i)_2Ru_2(\mu_2\text{-}SCH_2C_6H_4\text{-}p\text{-}Bu^t)_2(\mu_2\text{-}SC_6H_4\text{-}p\text{-}O)\text{-}(CO)(CH_2)_3(CO)\text{-}(p\text{-}O\text{-}C_6H_4\text{-}\mu_2S)Ru_2(\mu_2\text{-}SCH_2C_6H_4\text{-}p\text{-}Bu^t)(\eta^6\text{-}p\text{-}MeC_6H_4Pr^i)_2(\mu_2\text{-}SC_6H_4\text{-}p\text{-}O)\text{-}(CO)(CH_2)_3(CO)\text{-}(p\text{-}O\text{-}C_6H_4\text{-}\mu_2S)Ru_2(\mu_2\text{-}SCH_2C_6H_4\text{-}p\text{-}Bu^t)_2(\eta^6\text{-}p\text{-}MeC_6H_4Pr^i)_2]Cl_3$ ***(20)***

To a solution of ruthenium acid intermediate **19** (0.201 g, 0.181 mmol, 2.0 equiv.) in dry CH_2Cl_2 (20 mL) were added at r.t. under inert atmosphere (N_2), EDCI (0.044 g, 0.232 mmol, 2.56 equiv.), **5** (0.085 g, 0.091 mmol, 1 equiv.) and DMAP (0.004 g, 0.031mmol, 0.35 equiv.) and the reaction mixture was further stirred overnight. The reaction evolution was verified by ^{1}H-NMR and TLC and then the mixture was concentrated under reduced pressure. Purification by column chromatography using CH_2Cl_2/CH_3OH (9:1, v/v) allowed the isolation of **20** as an orange solid (0.045 g, 0.015 mmol, yield 16%).

C'- carbon atoms on the central binuclear ruthenium complex

^{1}H-NMR ($CDCl_3$) δ_H, ppm: 7.98 (2H, d, 2xS-(*Ar*)C'-C'$\underline{H}$-C'H-C'-O, $^3J_{H,H}$ = 8.6 Hz), 7.84 (2H, d, 2xS-(*Ar*)C'-C'$\underline{H}$-C'H-C'-O, $^3J_{H,H}$ = 8.6 Hz), 7.83 (4H, d, 4xS-(*Ar*)C-C$\underline{H}$-CH-C-O, $^3J_{H,H}$ = 8.6 Hz), 7.40-7.52 (20H, m, 8xS-CH_2-(*Ar*)C-C$\underline{H}$-CH-C-C(CH_3)$_3$, 8xS-CH_2-(*Ar*)C-CH-C$\underline{H}$-C-C(CH_3)$_3$, 2xS-C'H_2-(*Ar*)C'-C'$\underline{H}$-C'H-C'-C'(C'H_3)$_3$, 2xS-C'H_2-(*Ar*)C'-C'H-C'$\underline{H}$-C'-C'(C'H_3)$_3$, $^3J_{H,H}$ = 8.6 Hz), 7.20 (2H, d, 2xS-(*Ar*)C'-C'H-C'$\underline{H}$-C'-O, $^3J_{H,H}$ = 8.6 Hz), 7.11-7.14 (6H, m, 4xS-(*Ar*)C-CH-C$\underline{H}$-C-O, 2xS-(*Ar*)C'-C'H-C'$\underline{H}$-C'-O, $^3J_{H,H}$ = 8.6 Hz), 5.22 (2H, d, 2xC'H_3-(*Ar*)C'-C'H-C'$\underline{H}$-C', $^3J_{H,H}$ = 5.8 Hz), 5.16 (2H, d, 2xC'H_3-(*Ar*)C'-C'$\underline{H}$-C'H-C', $^3J_{H,H}$ = 5.9 Hz), 5.15 (4H, d, 4xCH_3-(*Ar*)C-CH-C$\underline{H}$-C, $^3J_{H,H}$ = 6.0 Hz), 5.08 (2H, d, 2xC'H_3-(*Ar*)C'-C'H-C'$\underline{H}$-C', $^3J_{H,H}$ = 5.9 Hz), 5.00 (4H, d, 4xCH_3-(*Ar*)C-CH-C$\underline{H}$-C, $^3J_{H,H}$ = 5.8 Hz), 4.95 (4H, d, 4xCH_3-(*Ar*)C-C$\underline{H}$-CH-C, $^3J_{H,H}$ = 5.7 Hz), 4.91 (2H, d, 2xC'H_3-(*Ar*)C'-C'$\underline{H}$-C'H-C', $^3J_{H,H}$ = 5.9 Hz), 4.62 (4H, d, 4xCH_3-(*Ar*)C-C$\underline{H}$-CH-C, $^3J_{H,H}$ = 5.9 Hz), 3.61 (6H, s, 2xS-C$\underline{H_2}$-(*Ar*)C-CH-CH-C-C(CH_3)$_3$, C'$\underline{H_2}$-(*Ar*)C'-C'H-C'H-C'-C'(C'H_3)$_3$), 3.43 (4H, s, 2xS-C$\underline{H_2}$-(*Ar*)C-CH-CH-C-C(CH_3)$_3$), 2.75-2.82 (8H, m, 2xS-(*Ar*)C-CH-CH-C-O-(C=O)-C$\underline{H_2}$-CH_2, 2xS-(*Ar*)C-CH-CH-C-O-(C=O)-(CH_2)$_2$-C$\underline{H_2}$, $^3J_{H,H}$ = 7.2 Hz), 2.16-2.28 (4H, m, 2xS-(*Ar*)C-CH-CH-C-O-(C=O)-CH_2-C$\underline{H_2}$, $^3J_{H,H}$ = 7.3 Hz), 1.92 (4H, sept, 4x(*Ar*)C-CH-CH-C-C$\underline{H}$(CH_3)$_2$, $^3J_{H,H}$ = 6.9 Hz), 1.87 (2H, sept, 2x(*Ar*)C'-C'H-C'H-C'-C'$\underline{H}$(C'H_3)$_2$, $^3J_{H,H}$ = 6.9 Hz), 1.77 (12H, s, 4xC$\underline{H_3}$-(*Ar*)C-CH-CH-C), 1.73 (6H, s, 2xC'$\underline{H_3}$-(*Ar*)C'-C'H-C'H-C'), 1.37 (18H, s, 2xS-CH_2-(*Ar*)C-CH-CH-C-C(C$\underline{H_3}$)$_3$), 1.33 (9H, s, S-C'H_2-(*Ar*)C'-C'H-C'H-C-C'(C'$\underline{H_3}$)$_3$), 1.33 (18H, s, 2xS-CH_2-(*Ar*)C-CH-CH-C-C(C$\underline{H_3}$)$_3$), 0.95 (12H, d,

2x(*Ar*)C-CH-CH-C-CH(C$\underline{H_3}$)$_2$, $^3J_{H,H}$ = 6.9 Hz), 0.87-0.92 (24H, m, 2x(*Ar*)C-CH-CH-C-CH(C$\underline{H_3}$)$_2$, 2x(*Ar*)C'-C'H-C'H-C'-C'H(C'$\underline{H_3}$)$_2$, $^3J_{H,H}$ = 6.9 Hz).

^{13}C-NMR (CDCl$_3$) (δ_C, ppm): 171.2 (4C, 2xS-(*Ar*)C-CH-CH-C-O-($\underline{C}$=O), 2xS-(*Ar*)C'-C'H-C'H-C'-O-($\underline{C}$=O)), 151.9 (2C, 2xS-CH$_2$-(*Ar*)C-CH-CH-$\underline{C}$-C(CH$_3$)$_3$), 151.8 (1C, S-C'H$_2$-(*Ar*)C'-C'H-C'H-$\underline{C}$'-C(CH$_3$)$_3$), 151.5 (2C, 2xS-CH$_2$-(*Ar*)C-CH-CH-$\underline{C}$-C(CH$_3$)$_3$), 151.2 (1C, S-(*Ar*)C'-C'H-C'H-$\underline{C}$'-O), 151.0 (2C, 2xS-(*Ar*)C-CH-CH-$\underline{C}$-O), 150.8 (1C, S-(*Ar*)C'-C'H-C'H-$\underline{C}$'-O), 136.7 (4C, 4xS-CH$_2$-(*Ar*)$\underline{C}$-CH-CH-C-C(CH$_3$)$_3$), 136.6 (1C, S-C'H$_2$-(*Ar*)$\underline{C}$'-C'H-C'H-C'-C'(C'H$_3$)$_3$), 135.9 (1C, S-(*Ar*)$\underline{C}$'-C'H-C'H-C'-O), 135.1 (2C, 2xS-(*Ar*)$\underline{C}$-CH-CH-C-O), 135.0 (1C, S-(*Ar*)$\underline{C}$'-C'H-C'H-C'-O), 133.8 (2C, 2xS-(*Ar*)C'-$\underline{C}$'H-C'H-C'-O), 133.7 (4C, 4xS-(*Ar*)C-$\underline{C}$H-CH-C-O), 133.6 (2C, 2xS-(*Ar*)C'-$\underline{C}$'H-C'H-C'-O), 129.4 (2C, 2xS-C'H$_2$-(*Ar*)C'-$\underline{C}$'H-C'H-C'-C'(C'H$_3$)$_3$), 129.3 (4C, 4xS-CH$_2$-(*Ar*)C-$\underline{C}$H-CH-C), 129.2 (4C, 4xS-CH$_2$-(*Ar*)C-$\underline{C}$H-CH-C), 125.7 (4C, 4xS-CH$_2$-(*Ar*)C-CH-$\underline{C}$H-C), 125.6 (2C, 2xS-C'H$_2$-(*Ar*)C'-C'H-$\underline{C}$'H-C'), 125.5 (4C, 4xS-CH$_2$-(*Ar*)C-CH-$\underline{C}$H-C), 122.6 (2C, 2xS-(*Ar*)C'-C'H-$\underline{C}$'H-C'-O), 122.5 (4C, 4xS-(*Ar*)C-CH-$\underline{C}$H-C-O), 122.4 (2C, 2xS-(*Ar*)C'-C'H-$\underline{C}$'H-C'-O), 107.3 (2C, 2xC'H$_3$-(*Ar*)C'-C'H-C'H-$\underline{C}$'), 107.1 (4C, 4xCH$_3$-(*Ar*)C-CH-CH-$\underline{C}$), 100.7 (4C, 4xCH$_3$-(*Ar*)$\underline{C}$-CH-CH-C), 100.5 (2C, 2xC'H$_3$-(*Ar*)$\underline{C}$'-C'H-C'H-C'), 85.1 (2C, 2xC'H$_3$-(*Ar*)C'-$\underline{C}$'H-C'H-C'), 84.5 (2C, 2xC'H$_3$-(*Ar*)C'-C'H-$\underline{C}$'H-C'), 84.28 (4C, 4xCH$_3$-(*Ar*)C-CH-$\underline{C}$H-C), 84.25 (2C, 2xC'H$_3$-(*Ar*)C'-C'H-$\underline{C}$'H-C), 83.71 (4C, 4xCH$_3$-(*Ar*)C-$\underline{C}$H-CH-C), 83.68 (4C, 4xCH$_3$-(*Ar*)C-CH-$\underline{C}$H-C), 83.0 (2C, 2xC'H$_3$-(*Ar*)C'-$\underline{C}$'H-C'H-C'), 82.6 (4C, 4xCH$_3$-(*Ar*)C-$\underline{C}$H-CH-C), 40.0 (2C, 2xS-$\underline{C}$H$_2$-(*Ar*)C-CH-CH-C-C(CH$_3$)$_3$), 39.6 (2C, 2xS-$\underline{C}$H$_2$-(*Ar*)C-CH-CH-C-C(CH$_3$)$_3$), 38.8 (1C, S-$\underline{C}$'H$_2$-(*Ar*)C'-C'H-C'H-C'-C'(C'H$_3$)$_3$), 34.85 (2C, 2xS-CH$_2$-(*Ar*)C-CH-CH-C-$\underline{C}$(CH$_3$)$_3$), 34.83 (1C, S-C'H$_2$-(*Ar*)C'-C'H-C'H-C'-$\underline{C}$'(C'H$_3$)$_3$), 34.81 (2C, 2xS-CH$_2$-(*Ar*)C-CH-CH-C-$\underline{C}$(CH$_3$)$_3$), 33.2 (4C, 2xS-CH$_2$-(*Ar*)C-CH-CH-C-O-(C=O)-$\underline{C}$H$_2$, 2x(S-CH$_2$-(*Ar*)C-CH-CH-C-O-(C=O)-(CH$_2$)$_2$-$\underline{C}$H$_2$), 31.65 (15, 4xS-CH$_2$-(*Ar*)C-CH-CH-C-C($\underline{C}$H$_3$)$_3$, S-C'H$_2$-(*Ar*)C'-C'H-C'H-C'-C'($\underline{C}$'H$_3$)$_3$), 30.9 (4C, 4x(*Ar*)CH-CH-C-$\underline{C}$H(CH$_3$)$_2$), 30.7 (2C, 2x(*Ar*)C'H-C'H-C'-$\underline{C}$'H(C'H$_3$)$_2$), 23.2 (4C, 2x(*Ar*)CH-CH-C-CH($\underline{C}$H$_3$)$_2$), 22.73 (2C, (*Ar*)C'H-C'H-C'-C'H($\underline{C}$'H$_3$)$_2$), 22.66 (4C, 2x(*Ar*)CH-CH-C-CH($\underline{C}$H$_3$)$_2$), 22.5 (2C, (*Ar*)C'H-C'H-C'-C'H($\underline{C}$'H$_3$)$_2$), 20.0 (2C, 2xS-(*Ar*)C-CH-CH-C-O-(C=O)-CH$_2$-$\underline{C}$H$_2$), 18.3 (4C, 4x$\underline{C}$H$_3$-(*Ar*)C-CH-CH-C), 18.1 (2C, 2x$\underline{C}$'H$_3$-(*Ar*)C'-C'H-C'H).

ESI-MS(+): *m/z* 1000.5312 [M-3Cl]$^{3+}$, calcd. for $C_{149}H_{187}O_8Ru_6S_9^{3+}$ 1000.3618, the isotopic pattern corresponds well to the calculated one.

R$_f$ (CH_2Cl_2/CH_3OH 9:1 (v/v)) = 0.66.

Elemental analysis (%): calcd. for $C_{149}H_{187}Cl_3O_8Ru_6S_9\cdot 0.5CH_2Cl_2\cdot 5H_2O$: C 55.42, H 6.16; found: C 55.32, H 6.18.

10. Compounds with three trithiolato-bridged ruthenium(II)-*p*-cymene units in a star-shape arrangement

Synthesis of $\{[(\eta^6\text{-}p\text{-}MeC_6H_4Pr^i)_2Ru_2(\mu_2\text{-}SCH_2C_6H_4\text{-}p\text{-}Bu^t)_2(\mu_2\text{-}SC_6H_4\text{-}p\text{-}NH)\text{-}(C{=}O)\text{-}(CH_2)_3\text{-}(C{=}O)\text{-}O\text{-}(CH_2)_2]_3NCl_3\}$ ***(23)***

To a solution of core linker **21** (0.038 g, 0.077 mmol, 1 equiv.) in CH_2Cl_2 (50 mL) at r.t. under inert atmosphere (N_2) were added HOBt·H_2O (0.037 g, 0.276 mmol, 3.6 equiv.) and DIPEA (0.06 mL, 0.347 mmol, 4.5 equiv.). The resulting mixture was stirred for 10 min and the ruthenium intermediate **4** (0.250 g, 0.253 mmol, 3.3 equiv.), EDCI (0.067 g, 0.347 mmol, 4.5 equiv.) and DIPEA (0.06 mL, 0.347 mmol, 9 equiv.) were added successively and the resulting reaction mixture was stirred for 24 h. The reaction evolution was verified by TLC and ^1H NMR. The solvent was removed under reduced pressure and the purification by flash column chromatography using CH_2Cl_2/CH_3OH (10:1, v/v) followed by purification on analytical TLC plates using CH_2Cl_2/CH_3OH (10:1, v/v) afforded **23** as an orange solid (0.054 g, 0.016 mmol, yield 20%).

^{1}H-NMR ($CDCl_3$) δ_H, ppm: 10.97 (3H, s, 3xS-(*Ar*)C-CH-CH-C-N$\underline{H}$), 8.06 (6H, d, 6xS-(*Ar*)C-CH-C$\underline{H}$-C-NH, $^3J_{H,H}$ = 8.7 Hz), 7.60 (6H, d, 6xS-(*Ar*)C-C$\underline{H}$-CH-C-NH, , $^3J_{H,H}$ = 8.7 Hz), 7.40-7.49 (24H, m, 12xS-CH_2-(*Ar*)C-C$\underline{H}$-CH-C-C$(CH_3)_3$, 12xS-CH_2-(*Ar*)C-CH-C$\underline{H}$-C-C$(CH_3)_3$), 5.00 (6H, d, 6xCH_3-(*Ar*)C-CH-C$\underline{H}$-C, $^3J_{H,H}$ = 5.8 Hz), 4.92 (6H, d, 6xCH_3-(*Ar*)C-CH-C$\underline{H}$-C, $^3J_{H,H}$ = 5.8 Hz), 4.74 (6H, d, 6xCH_3-(*Ar*)C-C$\underline{H}$-CH-C, $^3J_{H,H}$ = 5.7 Hz), 4.63 (6H, d, 6xCH_3-(*Ar*)C-C$\underline{H}$-CH-C, $^3J_{H,H}$ = 5.8 Hz), 4.13 (6H, t, 3xN-$(CH_2)_2$-C$\underline{H_2}$, $^3J_{H,H}$ = 5.6 Hz), 3.57 (6H, s, 3xS-C$\underline{H_2}$-(*Ar*)C-CH-CH-C-C$(CH_3)_3$), 3.38 (6H, s, 3xS-C$\underline{H_2}$-(*Ar*)C-CH-CH-C-C$(CH_3)_3$), 2.85 (6H, t, 3xN-C$\underline{H_2}$, $^3J_{H,H}$ = 5.6 Hz), 2.76 (6H, t, 3xCH_2-O-(C=O)-$(CH_2)_2$-C$\underline{H_2}$, $^3J_{H,H}$ = 7.2 Hz), 2.48 (6H, t, 3xCH_2-O-(C=O)-C$\underline{H_2}$, $^3J_{H,H}$ = 7.5 Hz), 2.07 (6H, m, 3xCH_2-O-(C=O)-CH_2-C$\underline{H_2}$, $^3J_{H,H}$ = 7.8 Hz), 2.00 (6H, sept, 6x(*Ar*)C-CH-CH-C-C$\underline{H}(CH_3)_2$, $^3J_{H,H}$ = 6.9 Hz), 1.69 (18H, s, 6xC$\underline{H_3}$-(*Ar*)C-CH-CH-C), 1.37 (27H, s, 3xS-CH_2-(*Ar*)C-CH-CH-C-C(C$\underline{H_3}$)$_3$), 1.34 (27H, s, 3xS-CH_2-(*Ar*)C-CH-CH-C-C(C$\underline{H_3}$)$_3$), 0.99 (12H, d, 3x(*Ar*)C-CH-CH-C-CH(C$\underline{H_3}$)$_2$, $^3J_{H,H}$ = 6.9 Hz), 0.94 (12H, d, 3x(*Ar*)C-CH-CH-C-CH(C$\underline{H_3}$)$_2$, $^3J_{H,H}$ = 6.9 Hz).

^{13}C-NMR ($CDCl_3$) δ_C, ppm: 173.6 (3C, 3xCH_2-O-(C=O)-$(CH_2)_3$-($\underline{C}$=O)-NH), 172.7 (3C, s, 3xCH_2-O-($\underline{C}$=O)-$(CH_2)_3$), 152.0, 151.9 (6C, 3xS-CH_2-(*Ar*)C-CH-CH-$\underline{C}$-C$(CH_3)_3$, 3xS-CH_2-(*Ar*)C-CH-CH-$\underline{C}$-C$(CH_3)_3$), 141.2 (3C, 3xS-(*Ar*)$\underline{C}$-CH-CH-C-NH), 136.8, 136.6 (6C, 3xS-CH_2-(*Ar*)$\underline{C}$-CH-CH-C-C$(CH_3)_3$, 3xS-CH_2-(*Ar*)$\underline{C}$-CH-CH-C-C$(CH_3)_3$), 132.8 (6C, 6xS-(*Ar*)C-$\underline{C}$H-CH-C-NH), 129.9 (3C, 3xS-(*Ar*)C-CH-CH-$\underline{C}$-NH), 129.1, 129.4 (12C, 6xS-CH_2-(*Ar*)C-$\underline{C}$H-CH-C-C$(CH_3)_3$, 6xS-CH_2-(*Ar*)C-$\underline{C}$H-CH-C-C$(CH_3)_3$), 125.7, 125.6 (12C, 6xS-CH_2-(*Ar*)C-CH-$\underline{C}$H-C-C$(CH_3)_3$, 6xS-CH_2-(*Ar*)C-CH-$\underline{C}$H-C-C$(CH_3)_3$), 120.3 (6C, 6xS-(*Ar*)C-CH-$\underline{C}$H-C-NH), 107.7 (6C, 6xCH_3-(*Ar*)C-CH-CH-$\underline{C}$), 100.2 (6C, 6xCH_3-(*Ar*)$\underline{C}$-CH-CH-C), 84.3 (6C, 6xCH_3-(*Ar*)C-CH-$\underline{C}$H-C), 83.9 (6C, 6xCH_3-(*Ar*)C-$\underline{C}$H-CH-C), 83.3 (6C, 6xCH_3-(*Ar*)C-CH-$\underline{C}$H-C), 82.3 (6C, 6xCH_3-(*Ar*)C-$\underline{C}$H-CH-C), 62.6 (3C, 3xN-CH_2-$\underline{C}H_2$),

53.3 (3C, 3xN-$\underline{C}H_2$), 39.9 (3C, 3xS-$\underline{C}H_2$-(*Ar*)C-CH-CH-C-C(CH_3)$_3$), 39.3 (3C, 3xS-$\underline{C}H_2$-(*Ar*)C-CH-CH-C-C(CH_3)$_3$), 36.4 (3C, 3xCH_2-O-(C=O)-(CH_2)$_2$-$\underline{C}H_2$), 34.94 (3C, 3xS-CH_2-(*Ar*)C-CH-CH-C-$\underline{C}$(CH_3)$_3$), 34.91 (3C, 3xS-CH_2-(*Ar*)C-CH-CH-C-$\underline{C}$(CH_3)$_3$), 33.9 (3C, 3xCH_2-O-(C=O)-$\underline{C}H_2$), 31.6 (18C, 6xS-CH_2-(*Ar*)C-CH-CH-C-C($\underline{C}H_3$)$_3$), 31.1 (6C, 6x(*Ar*)CH-CH-C-$\underline{C}$H(CH_3)$_2$), 23.2 (6C, 3x(*Ar*)CH-CH-C-CH($\underline{C}H_3$)$_2$), 23.0 (6C, 3x(*Ar*)CH-CH-C-CH($\underline{C}H_3$)$_2$), 21.1 (3C, 3xCH_2-O-(C=O)-CH_2-$\underline{C}H_2$), 18.2 (6C, 6x$\underline{C}H_3$-(*Ar*)C-CH-CH).
R$_f$ (CH_2Cl_2/CH_3OH 9:1 (v/v)) = 0.52.
ESI-MS(+): *m/z* found 1098.9557 [M-3Cl]$^{3+}$, calcd. for $C_{165}H_{218}N_3O_9Ru_6S_9^{3+}$ 1094.9475, the isotopic pattern corresponds well to the caculated one.
Elemental analysis (%): calcd. for $C_{165}H_{219}Cl_3N_4O_9Ru_6S_9 \cdot 2.5CH_2Cl_2$: C 55.63, H 6.24, N 1.55; found C 55.41, H 6.30, N 1.68.

Synthesis of {1,3,5{[(η^6-p-MeC$_6$H$_4$Pr^i)$_2$Ru$_2$(μ_2-SCH$_2$C$_6$H$_4$-p-Bu^t)$_2$(μ_2-SC$_6$H$_4$-p-NH)-(C=O)-(CH$_2$)$_3$-(C=O)-O-CH$_2$)}]$_3$C$_6$H$_3$}Cl$_3$ (24)

To a solution of core unit **22** (0.039 g, 0.077 mmol, 1 equiv.) in CH_2Cl_2 (50 mL) were added at r.t. under inert atmosphere (N_2) HOBt·H_2O (0.037 g, 0.276 mmol, 3.6 equiv.) and DIPEA (0.06 mL, 0.347 mmol, 4.5 equiv.) and the resulting mixture was stirred for 10 min. Then, **4** (0.250 g, 0.253 mmol, 3.3 equiv.), EDCI (0.067 g, 0.347 mmol, 4.5 equiv.) and DIPEA (0.06 mL, 0.347 mmol, 4.5 equiv.) were successively added and the resulting mixture was further stirred for 24 h. The reaction evolution was verified by TLC and ^{1}H-NMR. The solvent was removed under reduced pressure and purification by flash column chromatography using CH_2Cl_2/CH_3OH (10:1, v/v), followed by two purification on analytical TLC plates using CH_2Cl_2/CH_3OH (10:1, v/v) afforded **24** as an orange solid (0.100 g, 0.029 mmol, yield 38%).
^{1}H-NMR ($CDCl_3$) δ_H, ppm: 11.0 (3H, s, 3xS-(*Ar*)C-CH-CH-C-N$\underline{H}$), 8.03 (6H, d, 6xS-(*Ar*)C-CH-C$\underline{H}$-C-NH, $^3J_{H,H}$ = 8.7 Hz), 7.58 (6H, d, 6xS-(*Ar*)C-C$\underline{H}$-CH-C-NH, $^3J_{H,H}$ = 8.7 Hz), 7.37-7.47 (24H, m, 12xS-CH_2-(*Ar*)C-C$\underline{H}$-CH-C-C(CH_3)$_3$, 12xS-CH_2-(*Ar*)C-CH-C$\underline{H}$-C-C(CH_3)$_3$), 7.28 (3H, s, 3x(*Ar*)C-C$\underline{H}$), 5.10 (6H, s, 3x(*Ar*)C-C$\underline{H_2}$-O-(C=O)), 4.98 (6H, d, 6xCH_3-(*Ar*)C-C$\underline{H}$-CH-C, $^3J_{H,H}$ = 5.8 Hz), 4.89 (6H, d, 6xCH_3-(*Ar*)C-C$\underline{H}$-CH-C, $^3J_{H,H}$ = 5.8 Hz), 4.71 (6H, d, 6xCH_3-(*Ar*)C-CH-C$\underline{H}$-C, $^3J_{H,H}$ = 5.7 Hz), 4.60 (6H, d, 6xCH_3-(*Ar*)C-CH-C$\underline{H}$-C, $^3J_{H,H}$ = 5.7 Hz), 3.54 (6H, s, 3xS-C$\underline{H_2}$-(*Ar*)C-CH-CH-C-C(CH_3)$_3$), 3.35 (6H, s, 3xS-C$\underline{H_2}$-(*Ar*)C-CH-CH-C-C(CH_3)$_3$), 2.74 (6H, t, 3xCH_2-O-(C=O)-(CH_2)$_2$-C$\underline{H_2}$, $^3J_{H,H}$ = 7.0 Hz), 2.53 (6H, t, 3xCH_2-O-(C=O)-C$\underline{H_2}$, $^3J_{H,H}$ = 8.1 Hz), 2.07 (6H, t, 3xCH_2-O-(C=O)-CH_2-C$\underline{H_2}$, $^3J_{H,H}$ = 7.9 Hz), 1.97 (6H, sept, 6x(*Ar*)C-CH-CH-C-C$\underline{H}$(CH_3)$_2$, $^3J_{H,H}$ = 6.8 Hz), 1.66 (18H, s, 6xC$\underline{H_3}$-(*Ar*)C-CH-CH-C), 1.34 (27H, s, 3xS-CH_2-(*Ar*)C-CH-CH-C-C(C$\underline{H_3}$)$_3$), 1.32 (27H, s, 3xS-CH_2-(*Ar*)C-CH-CH-C-C(C$\underline{H_3}$)$_3$), 0.96 (12H, d, 3x(*Ar*)C-CH-CH-C-CH(C$\underline{H_3}$)$_2$, $^3J_{H,H}$ = 6.8 Hz), 0.91 (12H, d, 3x(*Ar*)C-CH-CH-C-CH(C$\underline{H_3}$)$_2$, $^3J_{H,H}$ = 6.8 Hz).

^{13}C-NMR ($CDCl_3$) δ_C, ppm: 173.3 (3C, 3xCH_2-O-(C=O)-$(CH_2)_3$-($\underline{C}$=O)-NH), 172.6 (3C, s, 3xCH_2-O-($\underline{C}$=O)-$(CH_2)_3$), 151.9, 151.8 (6C, 3xS-CH_2-(*Ar*)C-CH-CH-$\underline{C}$-$C(CH_3)_3$, 3xS-CH_2-(*Ar*)C-CH-CH-$\underline{C}$-$C(CH_3)_3$), 141.1 (3C, 3xS-(*Ar*)$\underline{C}$-CH-CH-C-NH), 137.0 (3C, 3x(*Ar*)$\underline{C}$-CH_2-O), 136.8, 136.5 (6C, 3xS-CH_2-(*Ar*)$\underline{C}$-CH-CH-C-$C(CH_3)_3$, 3xS-CH_2-(*Ar*)$\underline{C}$-CH-CH-C-$C(CH_3)_3$), 132.8 (6C, 6xS-(*Ar*)C-$\underline{C}$H-CH-C-NH), 129.9 (3C, 3xS-(*Ar*)C-CH-CH-$\underline{C}$-NH), 129.0, 129.3 (12C, 6xS-CH_2-(*Ar*)C-$\underline{C}$H-CH-C-$C(CH_3)_3$, 6xS-CH_2-(*Ar*)C-$\underline{C}$H-CH-C-$C(CH_3)_3$), 127.1 (3C, 3x(*Ar*)C-$\underline{C}$H), 125.6, 125.7 (12C, 6xS-CH_2-(*Ar*)C-CH-$\underline{C}$H-C-$C(CH_3)_3$, 6xS-CH_2-(*Ar*)C-CH-$\underline{C}$H-C-$C(CH_3)_3$), 120.2 (6C, 6xS-(*Ar*)C-CH-$\underline{C}$H-C-NH), 107.6 (6C, 6xCH_3-(*Ar*)C-CH-CH-$\underline{C}$), 100.2 (6C, 6xCH_3-(*Ar*)$\underline{C}$-CH-CH-C), 84.2 (6C, 6xCH_3-(*Ar*)C-CH-$\underline{C}$H-C), 83.8 (6C, 6xCH_3-(*Ar*)C-$\underline{C}$H-CH-C), 83.3 (6C, 6xCH_3-(*Ar*)C-CH-$\underline{C}$H-C), 82.2 (6C, 6xCH_3-(*Ar*)C-$\underline{C}$H-CH-C), 65.6 (3C, 3x(*Ar*)C-$\underline{C}H_2$-O-(C=O)), 39.9 (3C, 3xS-$\underline{C}H_2$-(*Ar*)C-CH-CH-C-$C(CH_3)_3$), 39.3 (3C, 3xS-$\underline{C}H_2$-(*Ar*)C-CH-CH-C-$C(CH_3)_3$), 36.2 (3C, 3xCH_2-O-(C=O)-$(CH_2)_2$-$\underline{C}H_2$-(C=O)-NH), 34.88 (3C, 3xS-CH_2-(*Ar*)C-CH-CH-C-$\underline{C}(CH_3)_3$), 34.85 (3C, 3xS-CH_2-(*Ar*)C-CH-CH-C-$\underline{C}(CH_3)_3$), 33.8 (3C, 3xCH_2-O-(C=O)-$\underline{C}H_2$), 31.52 (9C, 3xS-CH_2-(*Ar*)C-CH-CH-C-$C(\underline{C}H_3)_3$), 31.51 (9C, 3xS-CH_2-(*Ar*)C-CH-CH-C-$C(\underline{C}H_3)_3$), 31.1 (6C, 6x(*Ar*)CH-CH-C-$\underline{C}H(CH_3)_2$), 23.1 (6C, 3x(*Ar*)CH-CH-C-$CH(\underline{C}H_3)_2$), 22.9 (6C, 3x(*Ar*)CH-CH-C-$CH(\underline{C}H_3)_2$), 21.1 (3C, 3xCH_2-O-(C=O)-CH_2-$\underline{C}H_2$), 18.1 (6C, 6x$\underline{C}H_3$-(*Ar*)C-CH-CH).

R_f (CH_2Cl_2/CH_3OH 9:1 (v/v)) = 0.54.

ESI-MS(+): *m/z* found 1105.9443 $[M-3Cl]^{3+}$, calcd. for $C_{168}H_{216}N_3O_9Ru_6S_9^{3+}$ 1106.2756.

Elemental analysis (%): calcd. for $C_{168}H_{216}Cl_3N_3O_9Ru_6S_9 \cdot 1.3CH_3OH \cdot H_2O$: C 58.39, H 6.46, N 1.21; found C 58.37, H 6.61, N 1.16.

11. Stability in DMSO-*d*$_6$

The complexes are well soluble in DMSO, solvent used to prepare standard solutions for biological assays. To assess their stability, the compounds were dissolved in DMSO-*d*$_6$, and two ^{1}H-NMR spectra were recorded at 25°C 5 min and 28 days after sample preparation. Between the two experiments, the samples were stored at 0°C. For all complexes, there are no significant changes between the spectrum recorded after 5 min and the spectrum recorded after more than 30 days at 0°C, which indicates a very good stability of the complexes, and makes them suitable for further biological tests.

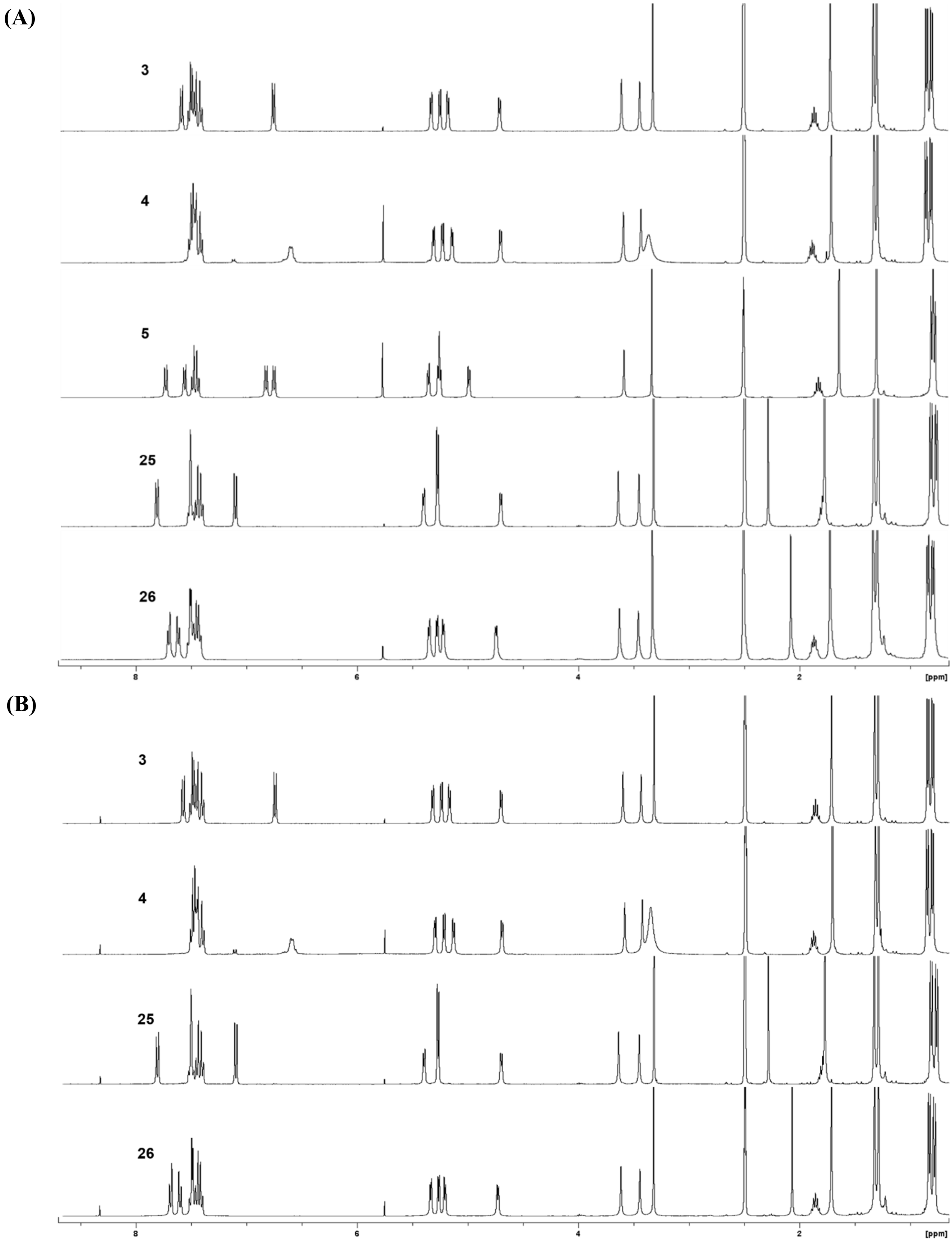

Figure S4. ^{1}H NMR Spectra of **3-5**, **25** and **26** recorded in DMSO-d$_6$ at 25°C; (A) recorded 5 min after sample preparation, and (B) sample after >30 days storage at 0-5°C in the dark.

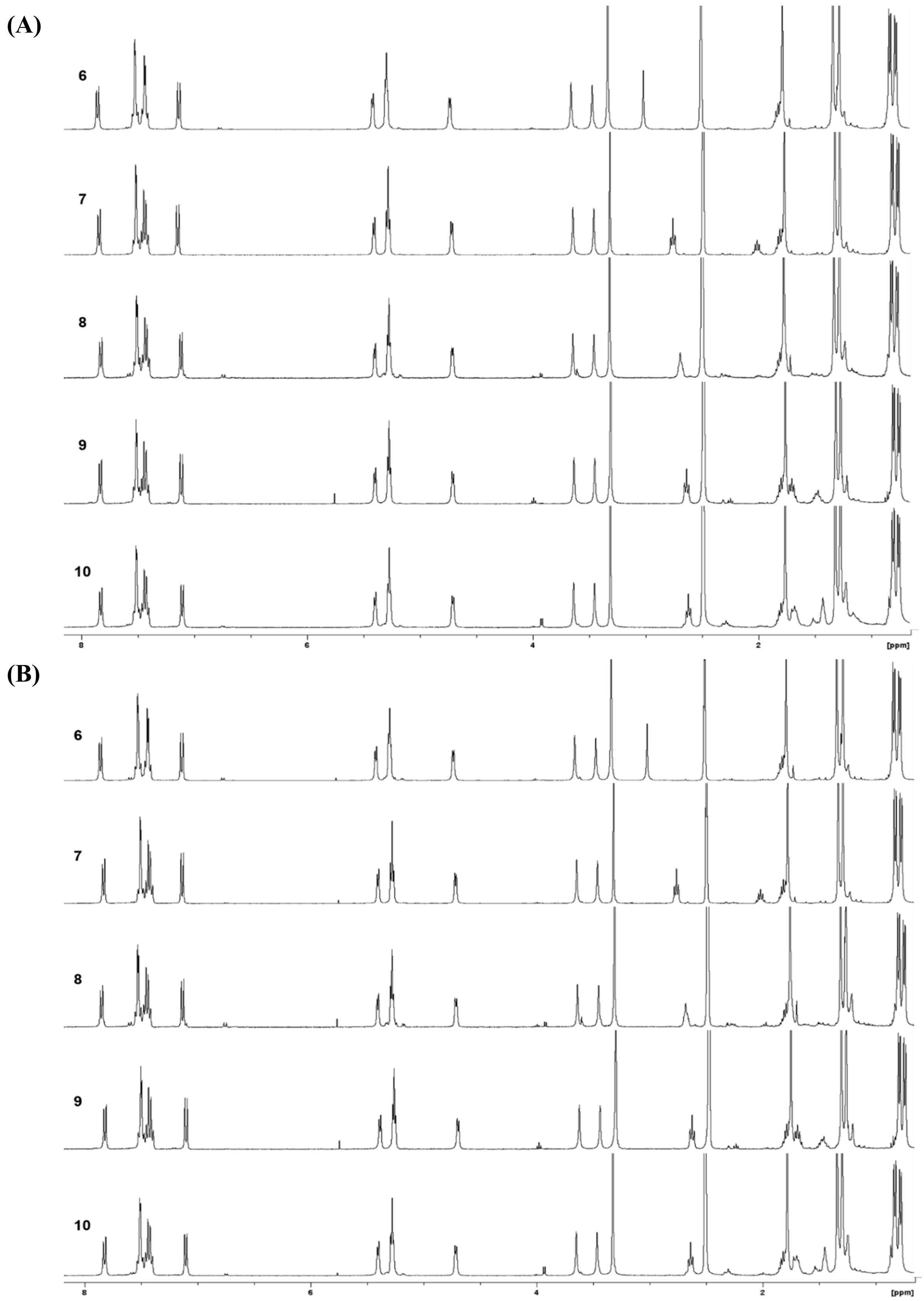

Figure S5. ^{1}H NMR spectra of **6-10** recorded in DMSO-d_6 at 25°C; (A) recorded 5 min after sample preparation, and (B) sample after >30 days storage at 0-5°C in the dark.

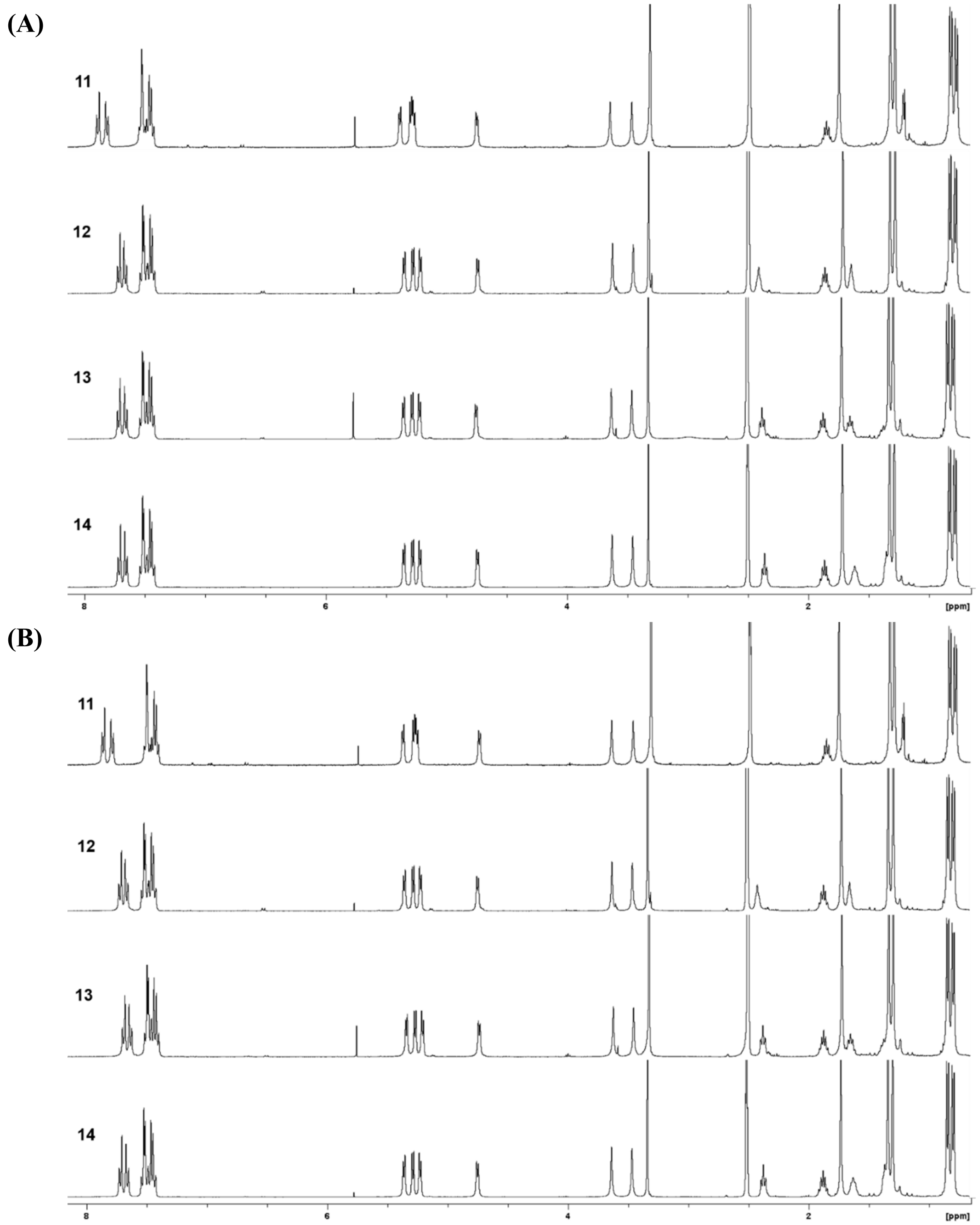

Figure S6. ^{1}H NMR spectra of **11-14** recorded in DMSO-d_6 at 25°C; (A) recorded 5 min after sample preparation, and (B) sample after >30 days storage at 0-5°C in the dark.

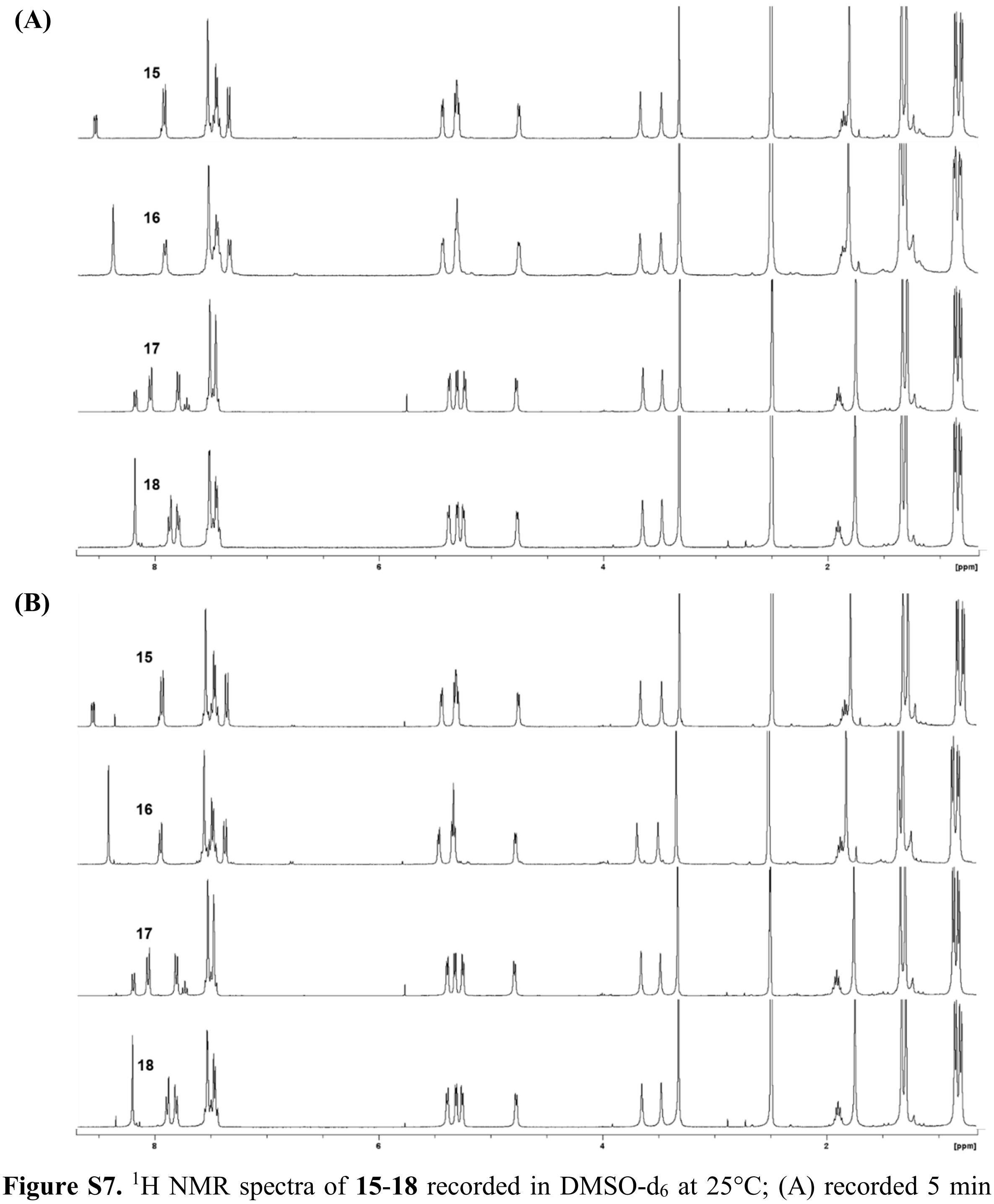

Figure S7. ^{1}H NMR spectra of **15-18** recorded in DMSO-d_6 at 25°C; (A) recorded 5 min after sample preparation, and (B) sample after >30 days storage at 0-5°C in the dark.

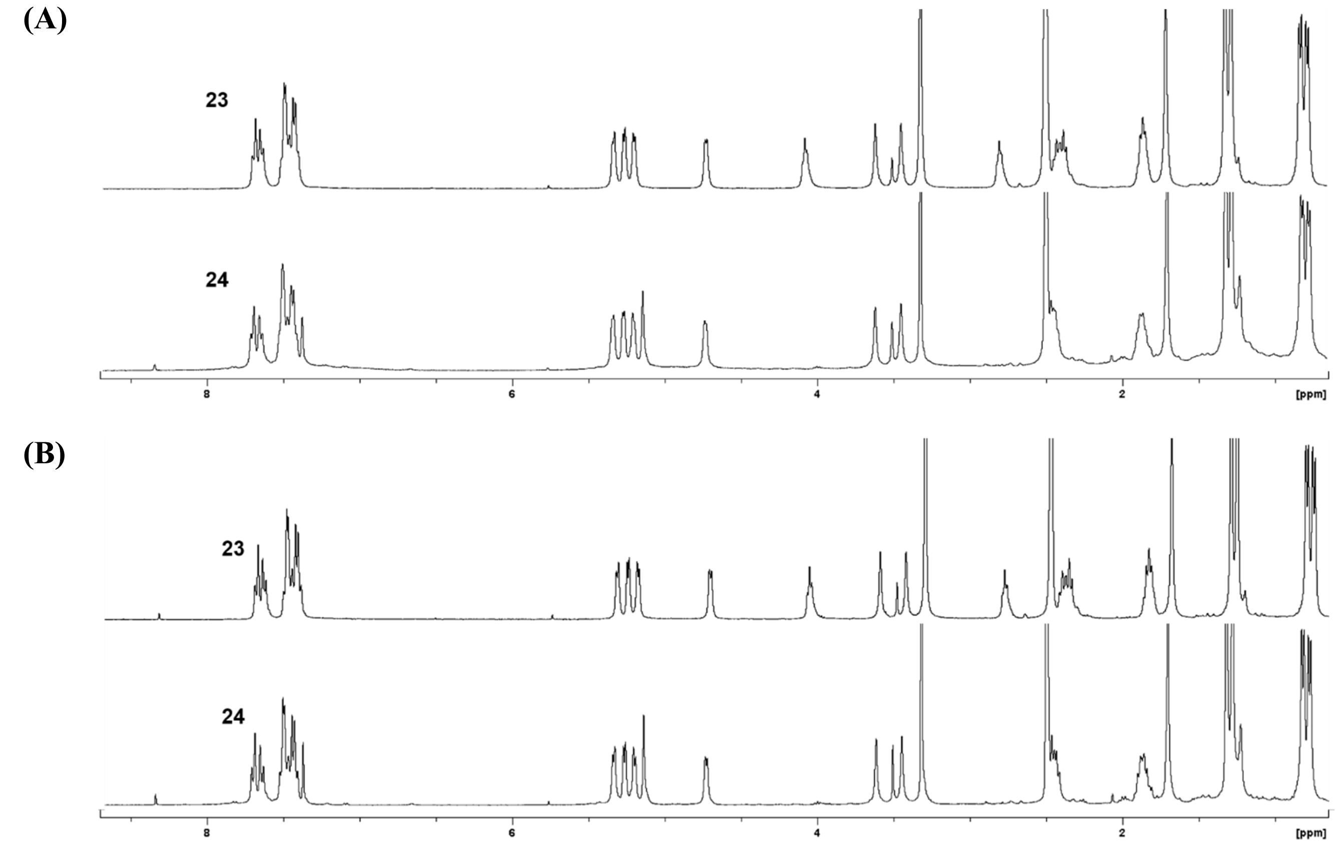

Figure S8. ^{1}H NMR spectra of **23** and **24** recorded in DMSO-d$_6$ at 25°C; (A) recorded 5 min after sample preparation, and (B) sample after >30 days storage at 0-5°C in the dark.

References

1. Păunescu, E.; Desiatkina, O.; Boubaker, G.; Anghel, N.; Amdouni, Y.; Hemphill, A.; Furrer, J., The quest of the best – A SAR study of trithiolato-bridged dinuclear ruthenium(II)-arene compounds presenting antiparasitic properties. *Manuscript in preparation* **2020**.
2. Desiatkina, O.; Paunescu, E.; Mosching, M.; Anghel, N.; Boubaker, G.; Amdouni, Y.; Hemphill, A.; Furrer, J., Coumarin-tagged dinuclear trithiolato-bridged ruthenium(II)arene complexes: photophysical properties and antiparasitic activity. *Chembiochem* **2020**, *21* (19), 2818-2835.
3. Bennett, M. A.; Smith, A. K., Arene ruthenium(II) complexes formed by dehydrogenation of cyclohexadienes with ruthenium(III) trichloride. *J. Chem. Soc. Dalton Trans.* **1974**, (2), 233-241.
4. Fulmer, G. R. M., A. J. M.; Sherden, N. H.; Gottlieb, H. E.; Nudelman, A.; Stoltz, B. M.; Bercaw, J. E.; Goldberg, K. I., , NMR Chemical shifts of trace impurities: common laboratory solvents, organics, and gases in deuterated solvents relevant to the organometallic chemist. *Organometallics* **2010**, *29* (9), 2176-2179.
5. Ibao, A.-F.; Gras, M.; Therrien, B.; Süss-Fink, G.; Zava, O.; Dyson, P. J., Thiolato-bridged arene ruthenium complexes: synthesis, molecular structure, reactivity, and anticancer activity of the dinuclear complexes [(arene)2Ru2(SR)2Cl2]. *Eur. J. Inorg. Chem.* **2012**, 1531-1535.

6. Giannini, F.; Furrer, J.; Süss-Fink, G.; Clavel, C. M.; Dyson, P. J., Synthesis, characterization and *in vitro* anticancer activity of highly cytotoxic trithiolato diruthenium complexes of the type [(h6-p-MeC6H4iPr)2Ru2(m2-SR1)2(m2-SR2)]+ containing different thiolato bridges. *J. Organomet. Chem.* **2013**, *744* 41-48.

© 2020 by the authors. Licensee MDPI, Basel, Switzerland. This article is an open access article distributed under the terms and conditions of the Creative Commons Attribution (CC BY) license (http://creativecommons.org/licenses/by/4.0/).
